# Porphyrin-based systems containing polyaromatic fragments: decoupling the synergistic effects in aromatic-porphyrin-fullerene systems†

**DOI:** 10.1039/d0ra07407a

**Published:** 2020-10-01

**Authors:** Sergio Ferrero, Héctor Barbero, Daniel Miguel, Raúl García-Rodríguez, Celedonio M. Álvarez

**Affiliations:** GIR MIOMeT, IU CINQUIMA/Química Inorgánica, Facultad de Ciencias, Universidad de Valladolid E-47011 Valladolid Spain celedonio.alvarez@uva.es raul.garcia.rodriguez@uva.es

## Abstract

In this work, we report a two-step synthesis that allows the introduction of four pyrene or corannulene fragments at the *para* position of *meso*-tetraarylporphyrins using a microwave-assisted quadruple Suzuki–Miyaura reaction. Placing the PAHs at this position, further from the porphyrin core, avoids the participation of the porphyrin core in binding with fullerenes. The fullerene hosting ability of the four new molecular receptors was investigated by NMR titrations and DFT studies. Despite having two potential binding sites, the pyrene derivatives did not associate with C_60_ or C_70_. In contrast, the tetracorannulene derivatives bound C_60_ and C_70_, although with modest binding constants. In these novel *para*-substituted systems, the porphyrin core acts as a simple linker that does not participate in the binding process, which allows the system to be considered as two independent molecular tweezers; *i.e.*, the first binding event is not transmitted to the second binding site. This behavior can be considered a direct consequence of the decoupling of the porphyrin core from the binding event.

## Abstract 2

Designed molecule for decoupling the synergistic supramolecular effects in aromatic-porphyrin-fullerene host–guest systems.

## Graphical abstract

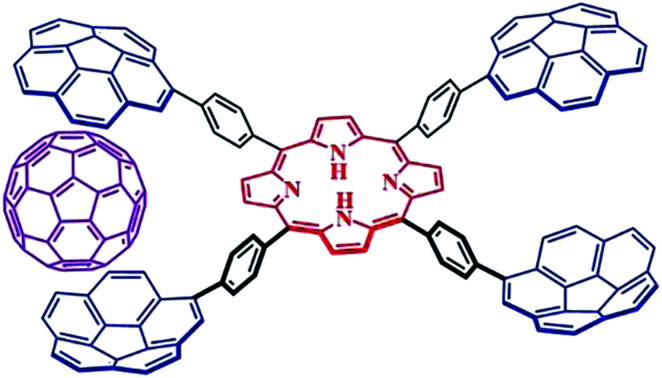

## Introduction

The rational design and development of functional complex architectures capable of interacting with nanometer-sized carbon allotropes,^1^ especially fullerenes, in a supramolecular fashion is a challenging current topic.^2^ However, the rational design of efficient systems of this type is not always straightforward, since it depends on a delicate balance of favorable and unfavorable interactions, and requires an understanding of the different subtle factors at play.^3^

Among the fullerenes, buckminsterfullerene (C_60_), and to a lesser extent, C_70_, have attracted the most attention. The iconic C_60_ fullerene is constructed of 12 pentagonal rings and 20 hexagonal ones, which give rise to its unique hollow spherical symmetry^4^ and electronic and magnetic proprieties.^5^ However, as a result of its non-planar π-surface, engineering supramolecular interaction of C_60_ with typical planar benzenoid rings is difficult. Therefore, in order to achieve efficient supramolecular interactions with C_60_, a host must exhibit both effective preorganization and a wide complementary surface in order to maximise the area of the convex–concave interaction.^3*a*,3*f*,6^ Among the curved polyaromatic hydrocarbons (PAHs),^6*a*,7^ corannulene has played a central role in fullerene recognition due to its excellent structural complementarity.^8^ However, pristine corannulene does not associate with C_60_ strongly enough for the adduct to be observed in solution.^9^ Therefore, the incorporation of corannulenes in different supramolecular frameworks is critical to achieve interactions with fullerene.^10^ Sygula's buckycatcher I, in which a cyclooctatetraene tether links two corannulene moieties, represented a breakthrough in the area.^11^ This molecular pincer was able to establish strong interactions with C_60_, sparking a search for other corannulene-based supramolecular architectures able to interact with C_60_. One obvious way to improve the interaction of a host with C_60_ is the introduction of more corannulene units. However, the seminal work by Sygula demonstrated that the introduction of more corannulene units might not necessarily improve the interaction with C_60_.^10*a*,12^ This and subsequent studies highlighted the importance of not only the preorganization of the molecular pincer, but also more subtle factors such as the flexibility of the scaffold and the interplay of different binding events.^13^

**Fig. 1:**
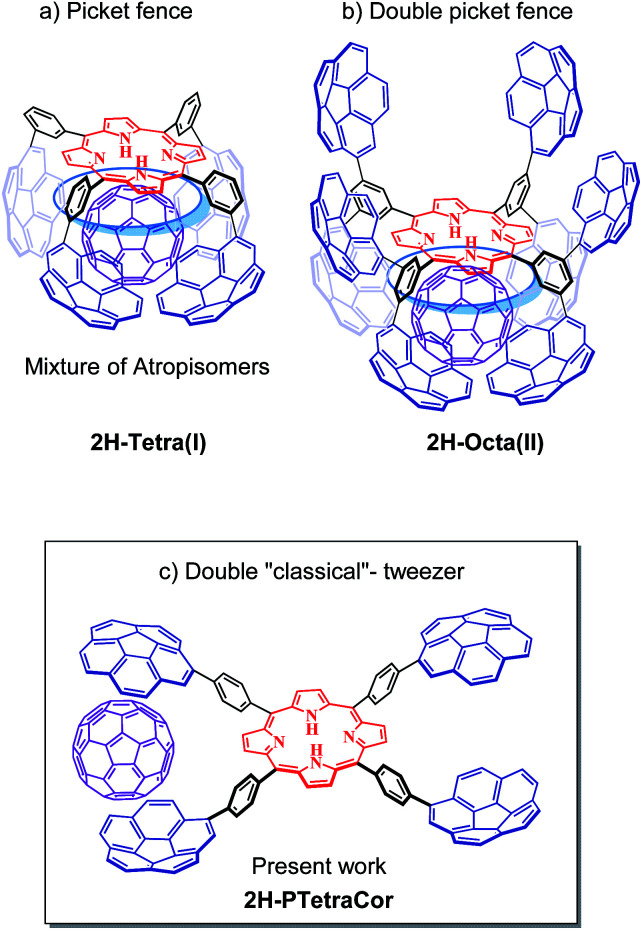
Corannulene tetraaryl porphyrin-based systems. (a and b) Previously reported “picket fence” and double “picket fence” systems resulting from the introduction of four or eight corannulene arms at the *meta* positions. (c) Tetracorannulene-based double tweezer system *via* substitution at the *para* position described in the present work. Note: the blue circle represents an idealized interaction of the guest with the porphyrin core in the *meta* substituted systems.

We have turned our attention to porphyrin-based systems as ideal scaffolds for the formation of corannulene-based receptors for fullerenes. Porphyrins and fullerenes are known to interact; however, the interactions between a single porphyrin core and fullerenes are usually too weak to be detected in solution.^14^ We have shown that the introduction of four corannulene arms at the *meta* positions of a tetraaryl *meso*-substituted porphyrin leads to a “picket fence” molecular clip able to bind C_60_ (Fig. 1a).^15^ However, the formation of four inseparable atropoisomers diminished the binding ability of the system and resulted in a difficult to analyse system. Nonetheless, this study highlighted the importance of the participation of the porphyrin core in the recognition process and inspired us to conduct subsequent studies based on this *meta*-substituted tetraaryl porphyrin scaffold.

In order to prevent the formation of atropisomers, we first introduced eight pyrene units (*i.e.*, in all *meta* positions),^16^ and, very recently eight corannulene units,^17^ at the *meta* positions of the tetraaryl porphyrin. Fig. 1b shows the resulting octapodal corannulene-based system, which features two binding sites as a result of the 4 + 4 arrangement of the corannulene arms. Although the compound was obtained in very low yield, this new supramolecular platform displayed very high affinity for C_60_ due to the involvement of the four corannulene units, and, importantly, the participation of the porphyrin core. However, the active participation of the porphyrin core led to the expansion of the second active site, which in turn led to its deactivation, as indicated by DFT calculations. In other words, while beneficial in maximizing the interaction with the first C_60_, the active participation of the porphyrin core led to a deactivating conformational change in the second binding site.

Herein, we explore the introduction of corannulene units at the *para*-position of a *meso*-tetraarylporphyrin with the simple idea of preparing a double “classical”-tweezer-based supra-molecular system with two potential binding sites (Fig. 1c). Introduction of the PAHs at the more remote *para* position in this new supramolecular scaffold should prevent cooperativity between the porphyrin core and the PAH pincers, and therefore provide a means to decouple the effect of porphyrin core in fullerene hosting.

## Results and discussion

The preparation of 2H-PTetraCor requires a porphyrin core that allows the easy introduction of four corannulene units at the *para* position of the tetraaryl *meso*-substituted porphyrin. We reasoned that the formation of such novel tetra-substituted systems could be accomplished *via* a tetra Suzuki reaction. In order to explore the feasibility of this synthetic scheme, we initially explored the formation of an analogous tetrapyrene-based scaffold (2H-PTetraPyr). Pyrene (Pyr) is less synthetically costly than corannulene and therefore offered a good starting point to test and optimize the synthetic route towards this new family of *para*-tetrasubstituted porphyrins. More importantly, 2H-PTetraPyr itself represents an additional framework-scaffold for supramolecular studies on fullerene hosting.

**Scheme 1:**
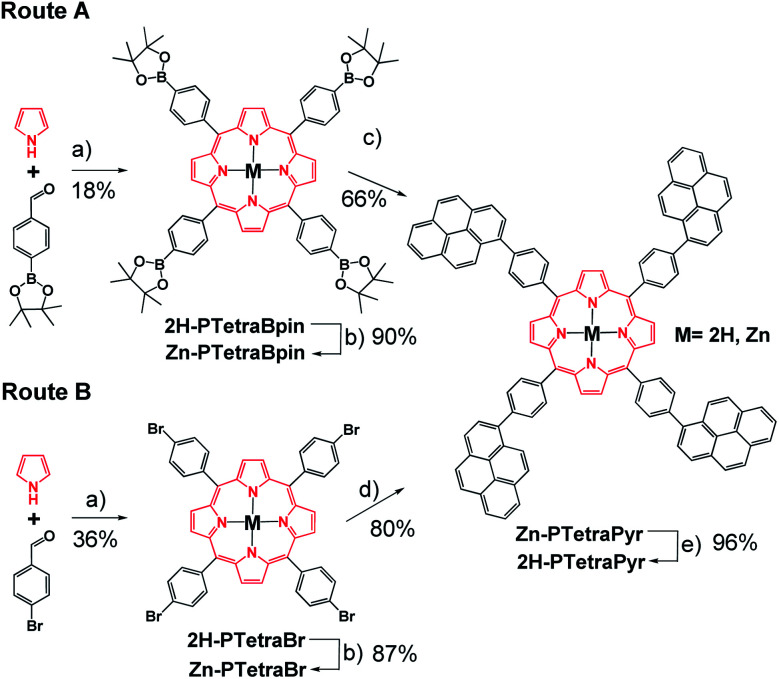
Overview of the two synthetic routes evaluated for 2H-PTetraPyr and Zn-PTetraPyr. Reagents and conditions: (a) propionic acid, nitrobenzene, MW, 200 °C; (b) Zn(OAc)_2_·2H_2_O, CHCl_3_, MW, 120 °C; (c) 1-bromopyrene, [PdCl_2_(dppf)], ^*t*^BuONa, toluene, MW, 130 °C; (d) 1-pinacol pyreneboronate (Pyr-Bpin), [PdCl_2_(dppf)], ^*t*^BuONa, toluene, MW, 130 °C; (e) CF_3_CO_2_H, CHCl_3_, RT.

A porphyrin with suitable reactive points in the *para* position is required for the construction of a more complex system *via* Suzuki reactions. Two alternatives were evaluated, based on the cross-coupling partners chosen for the Suzuki reaction. Route A involves the preparation of 2H-PTetraBpin with 1-bromopyrene as the cross-coupling partner, while route B utilises 2H-PTetraBr and 1-pinacol pyreneboronate (Pyr-Bpin) (Scheme 1). The synthesis of both porphyrin precursors was easily achieved by a microwave-assisted method starting from commercially available reagents. 2H-PTetraBr and 2H-PTetraBpin precipitated out from the reaction mixture and were obtained in 36% and 18% yield, respectively.^18^ Initial attempts to introduce four pyrene substituents into the porphyrin framework using both routes A and B led to a mixture of products that was complex to analyse and separate, and included homocoupling by-products and products with less than four Pyr substituents. However, metallation of the porphyrins with Zn(OAc)_2_·2H_2_O to give Zn-PTetraBr and Zn-PTetraBpin (step b in Scheme 1, which can be easily accomplished in excellent yields, 90%, see Experimental part) dramatically improved the introduction of the Pyr groups. In this fashion, the pyrene-tetrasubstituted porphyrin Zn-PTetraPyr was easily accessible in remarkably good yield *via* route B (80%; 66% *via* route A); this compound can be subsequently demetallated in virtually quantitative yield to produce 2H-PTetraPyr, thus providing two pyrene-based scaffolds.

**Scheme 2:**
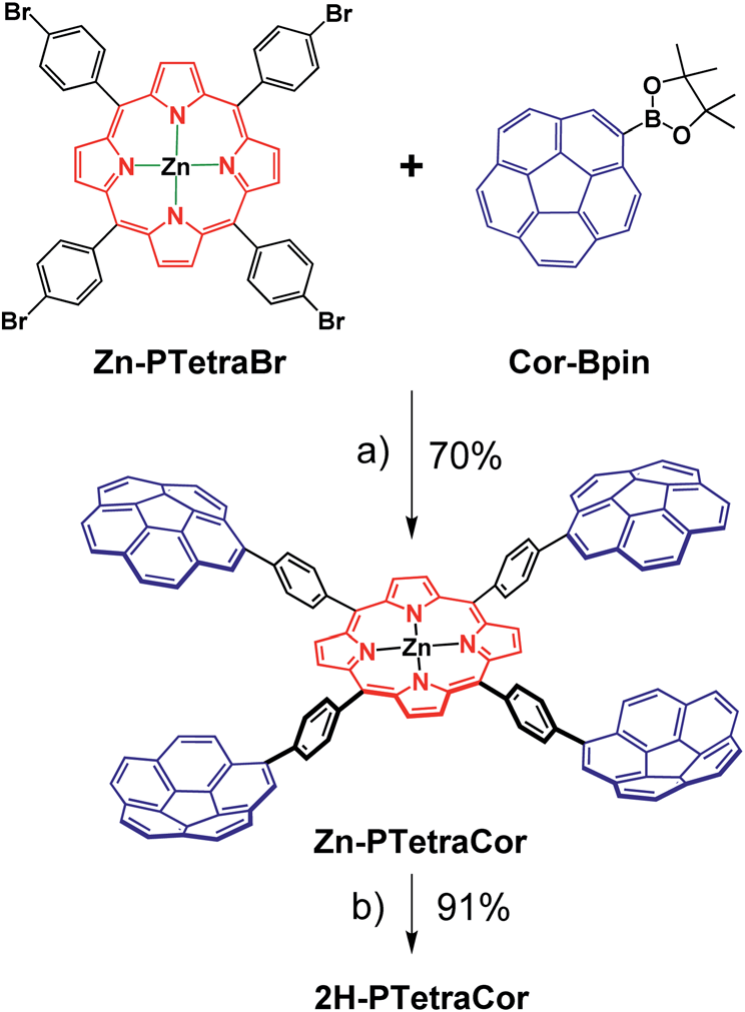
Optimized synthetic route for the tetracorannulene porphyrin systems 2H-PTetraCor and Zn-PTetraCor. Reagents and conditions: (a) [PdCl_2_(dppf)], ^*t*^BuONa, toluene, MW, 130 °C; (b) CF_3_CO_2_H, CHCl_3_, RT.

Having established and optimized the synthetic route for 2H-PTetraPyr, we turned our attention to introducing four corannulene units at the *para* positions of the porphyrin system using a similar microwave-assisted methodology. A method analogous to route B employing Cor-Bpin and Zn-PTetraBr was therefore selected. Zn-PTetraCor was obtained *via* this quadruple Suzuki reaction in 70% yield (Scheme 2). Quantitative demetallation furnished 2H-PTetraCor, thus providing an additional supramolecular scaffold. This yield is particularly good within the context of such systems and bearing in mind that four corannulene arms are introduced (*cf.* with 2H-Octa(II) (Fig. 1b), which was obtained in 15%). The new tetracorannulene derivatives were characterized using UV-vis, NMR, and HR-MS. The latter confirmed the formation of these compounds and showed the expected [M]^+^ peak at *m*/*z* 1606.4994 (calcd 1606.4969) and 1668.4085 (calcd 1668.4104), respectively, for 2H-PTetraCor and Zn-PTetraCor (see ESI Fig. S62 and S64†). In both cases, the ^1^H NMR spectra in CDCl_3_ showed the equivalence of the four corannulene units and the presence of only one β-pyrrole proton, as expected for the effective *D*_4h_ symmetry in solution. In the case of 2H-PTetraCor, a characteristic upfield-shifted broad signal was observed at −2.52 ppm for the NH groups. Remarkably, 2H-PTetraCor and Zn-PTetraCor exhibited sharp signals over a range of concentrations. This is in contrast to 2H-Octa(II), for which extensive broadening was observed due to intramolecular π–π interactions between pairs of corannulene units (leading to the potential formation of conformers), and thus suggests that these interactions are not present in 2H-PTetraCor and Zn-PTetraCor.

**Fig. 2:**
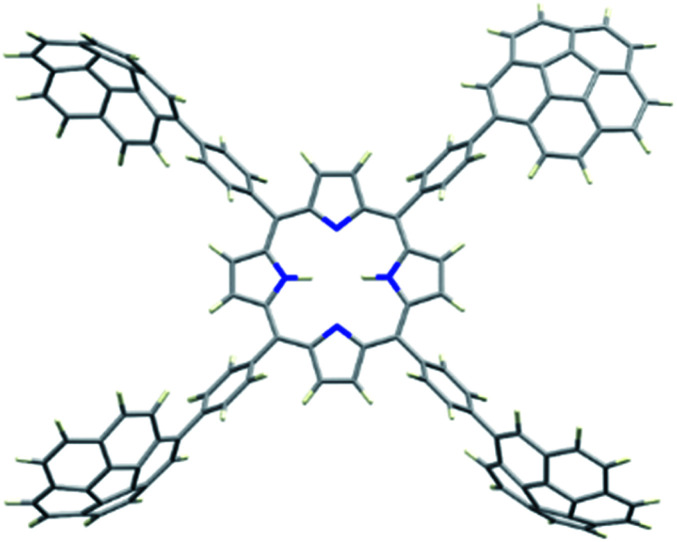
Optimized structure of compound 2H-PTetraCor. *α* angles are close to 90° (*α* = 90.85°). Note: *α* is defined as the angle formed by the corannulene-derived *meso* substituents with the centroid of the porphyrin as the vertex (see later discussion and Fig. 4).

2H-PTetraCor was investigated using DFT calculations, and Fig. 2 shows the most stable conformer (see ESI† for details). In this structure, the four tetraaryl moieties are approximately perpendicular to the planar porphyrin core, which is consistent with previously reported X-ray structures for similar *para*-substituted tetraaryl porphyrin systems.^19^ No π–π intramolecular interactions between corannulene moieties are observed. This is in contrast to 2H-Octa(II), in which the corannulene units could establish intramolecular interactions that had to be overcome prior to the inclusion of C_60_ and that were possible due to the bending of the porphyrin core, revealing its relatively flexible nature.^17^ In the case of 2H-PTetraCor, the two corannulene moieties on each side of the porphyrin are preorganized for C_60_ interaction and adopt an approximately concave–concave conformation with two potential binding sites, as shown in Fig. 2. The cleft formed by the two corannulene units is large enough (*ca.* 16 Å) to accommodate C_60_, suggesting that only a minor deformation of the system would be necessary to maximize its interaction with C_60_ (see later). It is also clear from the optimized structure that as expected, the porphyrin core is now far from the corannulene units, and therefore would not be expected to participate in the recognition process. Our new system is therefore predicted to behave like a “classic molecular tweezer”, in which the porphyrin is a simple linker, in contrast to our previous reported *meta*-substituted “picket fence” systems, in which the active participation of the porphyrin resulted in large binding constants due to its synergetic effect (Fig. 1).

In order to test these ideas and evaluate the decoupling of the porphyrin from the binding event, we next moved to study whether the new *para*-tetrasubstituted porphyrins could serve as supramolecular receptors for fullerenes, as suggested from DFT (Fig. 2). The pyrene-based systems Zn-PTetraPyr and 2H-PTetraPyr did not show any evidence of interaction with C_60_ or C_70_. Indeed, no change in their ^1^H NMR chemical shifts was observed in toluene-d_8_ after the addition of an excess of either of the fullerenes (10 equivalents). Although at first glance, this result is not unexpected, since planar PAHs are known to not exhibit supramolecular association with C_60_ due to their poor shape complementarity, it also suggests that the porphyrin core is not involved in the supramolecular recognition process in these *para* systems. In fact, in our previous work, *meta*-pyrene-substituted porphyrins^15,16^ showed host–guest formation, which was partially attributed to the participation of the porphyrin core, resulting in a cooperative effect with the PAH units.

Moving to the tetracorannulene systems, Zn-PTetraCor and 2H-PTetraCor exhibited changes in their chemical shifts upon the addition of C_60_ or C_70_. This was indicative of supramolecular association with the fullerene, which was now favoured by the presence of corannulene units instead of pyrene, since the corannulene moieties are ideally placed for effective complementary concave–convex π–π interactions with the C_60_ surface.

**Fig. 3:**
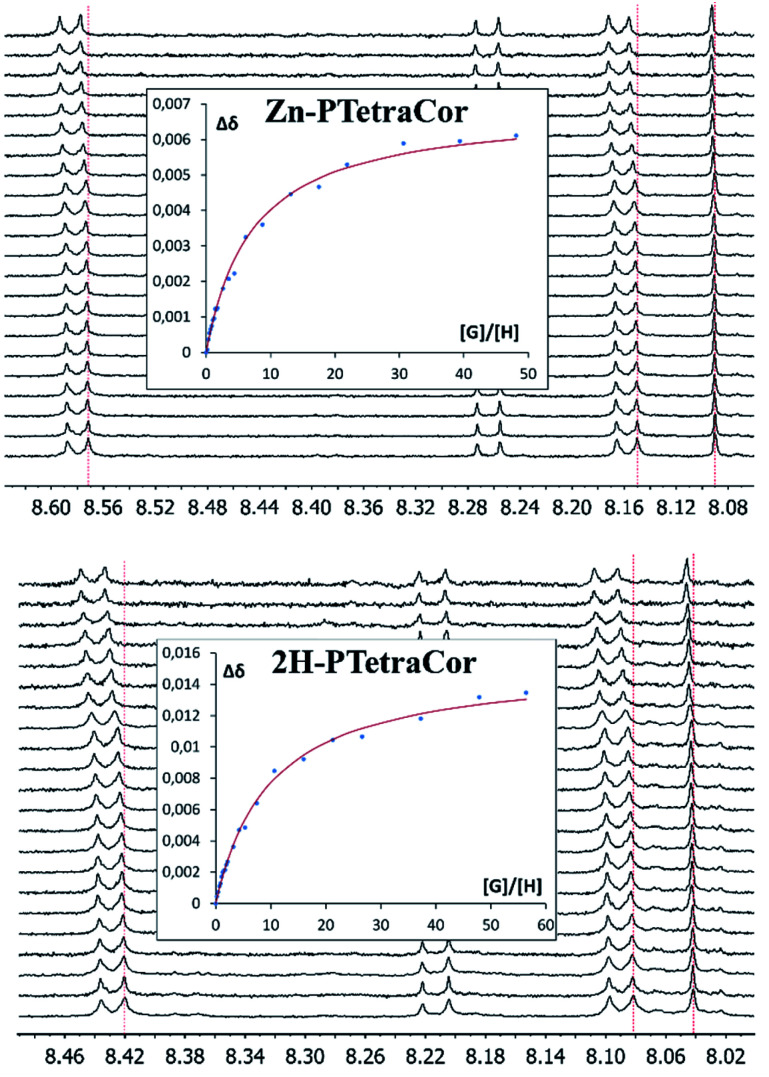
^1^H NMR spectra (500 MHz, toluene-d_8_) showing the H_6_, H_7_, and H_10_ chemical shifts (see ESI† for labelling scheme) of Zn-PTetraCor (top) and 2H-PTetraCor (bottom) upon the addition of aliquots of C_60_. Insets: plots of the changes in chemical shift against [G]/[H], where G (guest) is C_60_ and H (host) is Zn-PTetraCor or 2H-PTetraCor. The red line corresponds to the nonlinear fitting of Δ*δ* for H_6_ to a 1 : 1 binding isotherm. [H] = 10^−4^ M. The titration was carried out by adding known portions of a stock solution of C_60_ or C_70_ ([G] = 10^−3^ M). Note: the dotted red line over the spectrum is a guide for the eye.

To further explore the supramolecular behaviour of these new molecular receptors, titration experiments were carried out between Zn-PTetraCor or 2H-PTetraCor and C_60_ in toluene-d_8_. In both cases, the addition of aliquots of C_60_ resulted in a clear change in their ^1^H NMR chemical shifts, and several signals could be followed during the course of the titration, as shown in Fig. 3. The obtained nonlinear binding isotherms in the titrations of both receptors could be fitted to a 1 : 1 model (see insets in Fig. 3), yielding binding constants of *K*_1_ = 2.73 ± 0.02 × 10^2^ and *K*_1_ = 8.73 ± 0.05 × 10^1^, respectively, for Zn-PTetraCor and 2H-PTetraCor.^20^ The results of titrations with C_70_ paralleled those obtained with C_60_, and Table 1 summarizes the estimated binding constants obtained by fitting to a 1 : 1 system (however, the actual binding behaviour of these systems is believed to be more complex; see later discussion and ESI† for details). While both Zn-PTetraCor and 2H-PTetraCor exhibited association with C_70_, the association constants were very similar to those obtained for C_60_ (*i.e.*, these *para*-tetraaryl-porphyrins did not show discrimination between C_70_ or C_60_). Importantly, these constants are substantially smaller than those obtained for our 2H-Tetra(I) or 2H-Octa(II), in which the porphyrin core actively participated in the recognition process. In particular, they are approximately two orders of magnitude smaller in the case of binding to C_60_ (*cf.* 8.73 ± 0.05 × 10^1^ for 2H-PTetraCor*vs.* 5.4 ± 0.2 × 10^3^ (averaged value from atropisomers) for 2H-tetra(I) and 2.71 ± 0.08 × 10^4^ for 2H-Octa(II)).^15,17^ The relative difference is even greater for the association of C_70_ (*cf.* 5.24 ± 0.02 × 10^1^ for 2H-PTetraCor*vs.* 2.13 ± 0.10 × 10^5^ for 2H-Octa(II)).^17^

**Table 1:** Summary of apparent binding constants *K*_a_ (M^−1^) of 2H-PTetraCor and Zn-PTetraCor for different fullerenes obtained by fitting data from NMR titration in toluene-d_8_ at 298 K to a 1 : 1 model

	C_60_	C_70_
2H-PTetraCor	8.73 ± 0.05 × 10^1^	5.24 ± 0.02 × 10^1^
Zn-PTetraCor	2.73 ± 0.02 × 10^2^	2.16 ± 0.01 × 10^2^

Three main points can be extracted from these studies. (a) Although metal coordination is known to greatly affect the conformation of the porphyrin ring, the binding constants for 2H-PTetraCor and Zn-PTetraCor are similar (Table 1), and, more importantly, relatively small compared to those of our previously reported *meta*-substituted porphyrin systems. These facts suggest that the porphyrin core has little-to-no involvement in the recognition process. (b) Connected to this, the lack of discrimination between C_70_ and C_60_ in the present PTetraCor systems could also be traced, at least in part, to the lack of participation of the porphyrin core in the association event. (c) Despite the presence of two potential binding sites in these tetracorannulene systems (see Fig. 2), the titration experiments indicate 1 : 1 association and provide little evidence of 1 : 2 adducts (*i.e.*, both 2H-PTetraCor and Zn-PTetraCor apparently behave as single-tweezer molecular receptors for fullerenes).

**Fig. 4:**
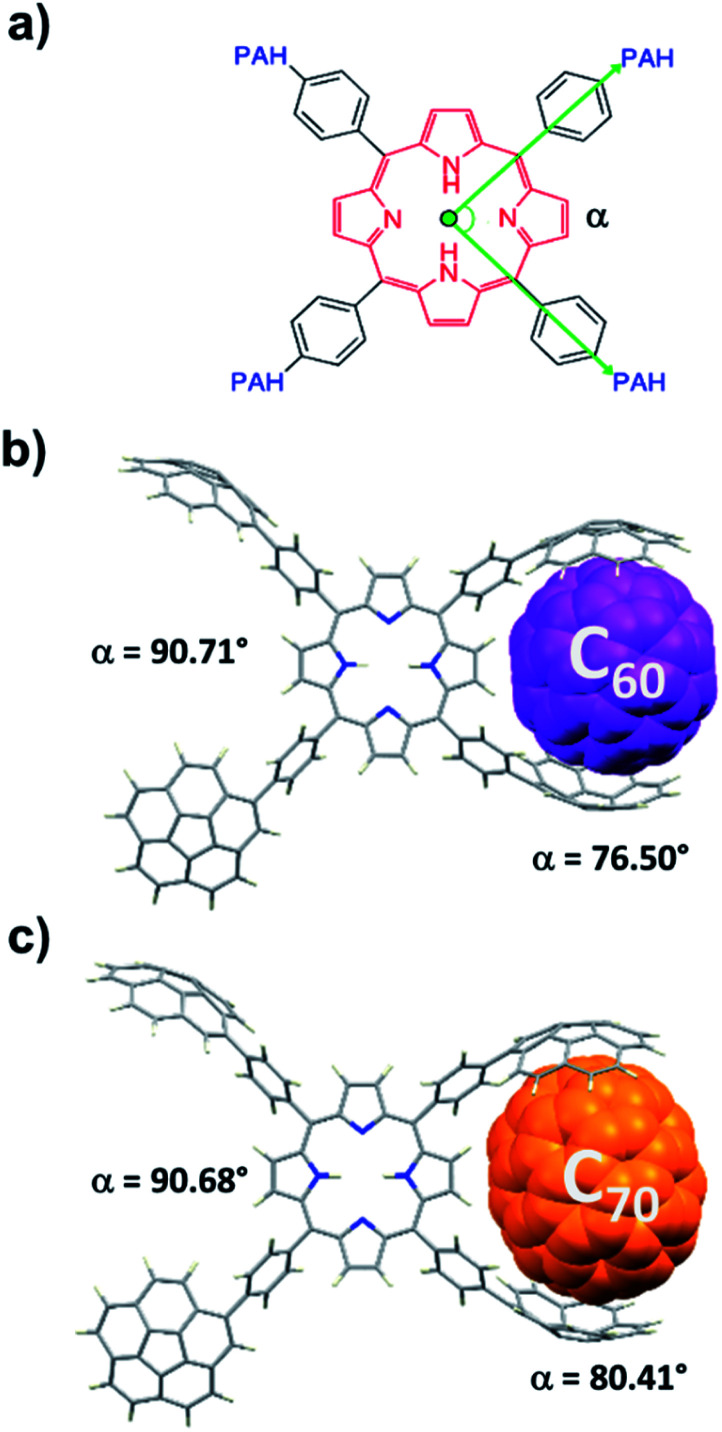
(a) Depiction of the angle (*α*) between the two *meso* substituents in the computed structures, where the green dot corresponds to the centroid of the porphyrin core and acts as the vertex. Computationally modelled structures of C_60_@2H-PTetraCor (b) and C_70_@2H-PTetraCor (c). Fullerenes are represented in spacefill style and colorized in purple and orange for C_60_ and C_70_, respectively.

To obtain further insight into the details of the recognition process of these novel *para*-substituted molecular receptors, we carried out computational DFT studies at the B97D3/6-31+G(d,p) level of theory.^21^ The optimized geometries of C_60_@2H-PTetraCor and C_70_@2H-PTetraCor are depicted in Fig. 4. The average distances between the inner concave surface of the corannulene fragments and outer convex surface of the fullerenes in the computed structures of the adducts C_60_@2H-PTetraCor and C_70_@2H-PTetraCor fell within the usual range for favourable dispersion interactions (≤3.5 Å).^22^ The calculated interaction energies are 43.75 kcal mol^−1^ and 44.25 kcal mol^−1^, respectively (see ESI† for details of the calculations). The very small difference between these energies (0.50 kcal mol^−1^) indicates that 2H-PTetraCor interacts almost identically with both fullerenes, which is in agreement with the experimentally observed lack of selectivity between C_60_ and C_70_ (see Table 1). In order to form the inclusion complexes, the two corannulene arms of one side of the molecule must reorganize slightly *via* C–C bond rotation to align with the convex outer surface of the guest, and the fullerene must come into close enough proximity with the host to establish significant attractive interactions in a tweezer-like fashion. A slightly more open tweezer would be expected in the case of C_70_ to account for its eccentricity (Fig. 4c). However, little deformation of the system is required to accommodate either C_60_ or C_70_. More specifically, the angle (*α*) formed by the corannulene-derived *meso* substituents with the centroid of the porphyrin as the vertex (see Fig. 4a) is 90.85° for 2H-PTetraCor.^19^ In order to interact with the fullerene, the two corannulene units move toward one another in a pincer fashion, resulting in a slight decrease in *α* (76.50° and 80.41° for C_60_@2H-PTetraCor and C_70_@2H-PTetraCor, Fig. 4b and c, respectively), whereas the angle *α* formed by the two corannulene units not engaged in supramolecular binding remains almost unaltered (90.71° and 90.68°, respectively, for C_60_@2H-PTetraCor and C_70_@2H-PTetraCor). That is, the first binding event (*i.e.*, the small compression of one pincer) is not conformationally transmitted to the second binding site of the molecule. The deformation energy, *i.e.*, the energy required for the host to adapt its conformation for fullerene binding, was very accessible and was estimated to be 5.55 kcal mol^−1^ and 3.78 kcal mol^−1^ for the C_60_@2H-PTetraCor and C_70_@2H-PTetraCor assemblies, respectively (see ESI† for further information).

We also studied the effect of the interaction with a second fullerene and optimized the adducts (C_60_)_2_@2H-PTetraCor and (C_70_)_2_@2H-PTetraCor, which were not observed experimentally, in order to estimate the deformation energies for the second binding event (see ESI† for details). The calculated values of 9.04 kcal mol^−1^ and 7.41 kcal mol^−1^ are only slightly larger than those estimated for the association of one C_60_ or one C_70_, respectively. In other words, the association of the first fullerene does not substantially affect the second binding site, and they can be considered as two approximately independent binding sites. The small increase in deformation energy for the second event, along with the very modest binding constants (see Table 1 for *K*_1_, and note that on purely statistical grounds, *K*_2_ would be 4 times lower than *K*_1_ for two completely independent sites) could help to rationalize the observed “apparent” 1 : 1 binding association by NMR titrations despite the fact that the two binding sites act approximately independently (*i.e.*, not in an inhibitory fashion).^23^

Along with the previous discussion, DFT calculations revealed the effects of the lack of participation of the porphyrin core in the binding to fullerene. Decoupling the influence of the porphyrin tether from the molecular pincer formed by the corannulenes has profound implications for the current *para*-tetrasubstituted porphyrin system. (1) The porphyrin core now acts merely as a linker and is not involved in the recognition process, resulting in lower binding energy and the loss of cooperativity between the porphyrin and the polyaromatic fragment. This is reflected in the comparatively lower binding constants observed experimentally. (2) A secondary effect connected to this is that the porphyrin does not transmit the information of the first binding event to the second binding site, and the system can be considered to consist of two independent pincers. This is in contrast to the large inhibitory effect observed for 2H-Octa(II), in which the active participation of the porphyrin in C_60_ or C_70_ binding, although beneficial for attaining high affinities towards C_60_ and C_70_, strongly inhibited the binding of a second fullerene. In the 2H-Octa(II) system, the involvement of the porphyrin resulted in the compression of one face of the porphyrin concomitant with the expansion of the second binding site (Fig. 1b). Finally, the lack of discrimination between C_60_ and C_70_ in the present systems most likely stems, at least in part, from the lack of participation of the porphyrin core in the binding, thus revealing more subtle implications than would be anticipated for the simple decoupling of the porphyrin from the binding event.

## Conclusions

By using a synthetic strategy based on a quadruple Suzuki–Miyaura reaction, we were able to access *meso*-tetraaryl substituted porphyrins in which four pyrene or corannulene fragments were installed relatively distant from the porphyrin core in the *para* positions. Both NMR titration experiments and density functional theory (DFT) calculations indicate that the porphyrin core is not involved in the binding event. While the tetrapyrene-porphyrin-based systems do not host fullerenes (either C_60_ or C_70_), the corannulene-based ones 2H-PTetraCor and Zn-PTetraCor can act as “classical molecular tweezers” with two approximately independent pincers, as supported by DFT studies. The fact that this system exhibits apparent 1 : 1 association is attributed mainly to the very low binding constants rather than a strong inhibitory effect, in contrast to our previously reported *meta*-substituted “picket fence” system. Therefore, although detrimental in terms of fullerene binding ability, this “decoupling” of the porphyrin core can also present benefits, such as the prevention of large structural changes that inhibit the potential second binding event. These findings highlight the important role of the tether in the construction of complex supramolecular systems, along with the interplay of different binding events in the fullerene recognition process. We hope these results will lead to a better understanding of the complexity in porphyrin-based supramolecular systems and help to pave the way for the rational design of new and functional complex supramolecular platforms.

## Experimental

### General methods

All reagents were purchased from commercial sources and used without further purification. Solvents were either used as received or dried according to procedures described elsewhere.^24^ Microwave reactions were carried out using an Anton Paar Monowave 300 Reactor. Column chromatography was carried out using silica gel 60 (particle size 0.040–0.063 mm; 230–400 mesh) as the stationary phase, and TLC was performed on precoated silica gel plates (0.25 mm thick, 60 F254) and visualized under UV light and/or by immersion in anisaldehyde. NMR spectra were recorded on a 400 MHz MR Agilent or 500 MHz Agilent DD2 instrument equipped with a OneNMR probe. NMR titrations were recorded on 500 MHz Agilent DD2 instruments equipped with a cold probe in the Laboratory of Instrumental Techniques (LTI) Research Facilities, University of Valladolid. ^1^H and ^13^C NMR chemical shifts (*δ*) are reported in parts per million (ppm) and are referenced to tetramethylsilane (TMS) using the solvent residual peak as an internal reference. Coupling constants (*J*) are reported in Hz. Standard abbreviations are used to indicate multiplicity: br = broad, s = singlet, d = doublet, t = triplet, m = multiplet. ^1^H and ^13^C peak assignments were performed using 2D NMR methods (^1^H–^1^H COSY, ^1^H–^1^H DQFCOSY, band-selective ^1^H–^1^H-ROESY, band-selective ^1^H–^13^C HSQC, band selective ^1^H–^13^C HMBC). Due to low solubility, some carbon signals were detected indirectly *via*^1^H–^13^C-HSQC/HMBC experiments; in these cases, the abbreviation *in* is used. High-resolution mass spectra were recorded at the mass spectrometry service of the Laboratory of Instrumental Techniques of the University of Valladolid using a Bruker Autoflex Speed MALDI-TOF system (N_2_ laser: 337 nm, pulse energy 100 μJ, 1 ns; acceleration voltage: 19 kV, reflector positive mode). *Trans*-2-[3-(4-*tert*-butylphenyl)-2-methyl-2-propenylidene]malonitrile (DCTB), 1,8,9-anthra-cenetriol, and 1,8-dihydroxy-9(10*H*)-anthracenone (dithranol) were used as matrixes. UV/vis spectra were recorded using a Shimadzu UV-1603 with spectrophotometric grade solvents. UV/vis absorption spectral wavelengths (*λ*) are reported in nanometers (nm), and molar absorption coefficients (*ε*) are reported in M^−1^ cm^−1^. Corannulene, 1-pinacol corannulene-boronate (Cor-Bpin) and 1-pinacol pyreneboronate (Pyr-Bpin) were obtained following literature procedures.^16,17,25^

### 2H-PTetraBr

Pyrrole (278 μL, 4 mmol), *p*-bromobenzaldehyde (740 mg, 4 mmol), propionic acid (13 mL, 173 mmol), and nitrobenzene (7 mL, 68 mmol) were mixed in a sealed vessel specifically designed for microwave irradiation. The mixture was stirred inside a microwave reactor at 200 °C for 15 min. The resulting dark crude was stored in a refrigerator for two days, and then filtered using a Büchner funnel and washed with MeOH. The obtained solid was placed in an oven under reduced pressure (190 °C, 2 h, 50 mbar) to remove residual nitrobenzene. Finally, the dark purple solid 2H-PTetraBr was obtained (339 mg, 36% yield). The spectroscopic data agreed with those reported in the literature.^18*b*^^1^H NMR (500 MHz, chloroform-*d*): *δ* 8.84 (s, 8H, H_2_), 8.07 (d, *J* = 8.3 Hz, 8H, H_6_), 7.91 (d, *J* = 8.3 Hz, 8H, H_7_), −2.87 (br, 2H, H_1_). ^13^C{^1^H} NMR (101 MHz, chloroform-*d*): *δ* 140.79 (C_5_), 135.78 (C_6_), 131.14 (C_2_), 129.95 (C_7_), 122.61 (C_8_), 118.95 (C_4_). HR-MS (MALDI-TOF): *m*/*z* for C_44_H_26_Br_4_N_4_ [M]^+^ calcd: 929.8883, found: 929.8851 (3.2 ppm error). UV/vis (toluene): *λ* 401 (*ε* = 76 900), 421 (*ε* = 384 900), 515 (*ε* = 20 100), 550 (*ε* = 9300), 592 (*ε* = 6300), 648 (*ε* = 4200).

### Zn-PTetraBr

2H-PTetraBr (114 mg, 0.12 mmol) and Zn(AcO)_2_·2H_2_O (39 mg, 0.18 mmol) were mixed in a sealed vessel specifically designed for microwave irradiation, and 5.5 mL of CHCl_3_ was added. The mixture was stirred inside the microwave reactor at 120 °C for 150 min. Subsequently, the crude was dissolved in 40 mL of CHCl_3_, placed in a separatory funnel, and washed with 20 mL of H_2_O. The organic layer was removed under low pressure to give the purple solid Zn-PTetraBr (105 mg, 87% yield). The spectroscopic data agreed with those reported in the literature.^18*b*^^1^H NMR (500 MHz, chloroform-*d*): *δ* 8.94 (s, 8H, H_2_), 8.07 (d, *J* = 8.3 Hz, 8H, H_6_), 7.90 (d, *J* = 8.3 Hz, 8H, H_7_). ^13^C{^1^H} NMR (101 MHz, chloroform-*d*): *δ* 149.99 (C_3_), 141.43 (C_5_), 135.66 (C_6_), 132.04 (C_2_), 129.80 (C_7_), 122.35 (C_8_), 119.93 (C_4_). HR-MS (MALDI-TOF): *m*/*z* for C_44_H_24_Br_4_N_4_Zn [M]^+^ calcd: 993.7964, found: 993.7985 (2.1 ppm error). UV/vis (toluene): *λ* 401 (*ε* = 32 500), 426 (*ε* = 162 600), 551 (*ε* = 21 500), 591 (*ε* = 5300).

### 2H-PTetraBpin

Pyrrole (173 μL, 2.5 mmol), 4-formylphenylboronic acid-pinacol ester (580 mg, 2.5 mmol), propionic acid (7 mL, 93 mmol), and nitrobenzene (13 mL, 126 mmol) were mixed in a sealed vessel specifically designed for microwave irradiation. The mixture was stirred inside a microwave reactor at 200 °C for 15 min. The dark crude was stored in a refrigerator for two days, and then filtered using a Büchner funnel and washed with hexane. The obtained solid was placed in an oven under reduced pressure (180 °C, 2 h, 50 mbar) to remove residual nitrobenzene. Finally, the dark purple solid 2H-PTetraBpin was obtained (127 mg, 18% yield). The spectroscopic data agreed with those reported in the literature.^18*a*^^1^H NMR (500 MHz, chloroform-*d*): *δ* 8.82 (s, 8H, H_2_), 8.22 (d, *J* = 8.0 Hz, 8H, H_6_), 8.18 (d, *J* = 8.0 Hz, 8H, H_7_), 1.50 (s, 48H, H_10_), −2.81 (br, 2H, H_1_). ^13^C{^1^H} NMR (101 MHz, chloroform-*d*): *δ* 145.03 (C_5_), 134.03 (C_6_), 132.97 (C_7_), 131.06 (C_2_), 127.96 (C_8_), 120.06 (C_4_), 84.07 (C_9_), 25.03 (C_10_). HR-MS (MALDI-TOF): *m*/*z* for C_68_H_74_B_4_N_4_O_8_ [M]^+^ calcd: 1118.5921, found: 1118.5958 (3.7 ppm error). UV/vis (toluene): *λ* 401 (*ε* = 74 000), 422 (*ε* = 426 600), 516 (*ε* = 20 700), 551 (*ε* = 9600), 592 (*ε* = 5500), 648 (*ε* = 3800).

### Zn-PTetraBpin

2H-PTetraBpin (35 mg, 0.031 mmol) and Zn(AcO)_2_·2H_2_O (55 mg, 0.25 mmol) were mixed in a sealed vessel specifically designed for microwave irradiation, and 2 mL of CHCl_3_ was added. The mixture was stirred inside the microwave reactor at 137 °C for 55 min. Subsequently, the crude was dissolved in 12 mL of CHCl_3_, placed in a separatory funnel, and washed with 10 mL of H_2_O. The organic layer was removed under low pressure to give the purple solid Zn-PTetraBpin (33 mg, 90% yield). ^1^H NMR (500 MHz, chloroform-*d*): *δ* 8.92 (s, 8H, H_2_), 8.23 (d, *J* = 8.0 Hz, 8H, H_6_), 8.19 (d, *J* = 8.0 Hz, 8H, H_7_), 1.50 (s, 48H, H_10_). ^13^C{^1^H} NMR (101 MHz, chloroform-*d*): *δ* 149.95 (C_3_), 145.68 (C_5_), 133.93 (C_6_), 132.86 (C_7_), 131.98 (C_2_), 127.74 (C_8_-*in*), 121.07 (C_4_), 84.05 (C_9_), 25.03 (C_10_). HR-MS (MALDI-TOF): *m*/*z* for C_68_H_72_B_4_N_4_O_8_Zn [M]^+^ calcd: 1180.5058, found: 1180.5036 (−2.2 ppm error). UV/vis (toluene): *λ* 401 (*ε* = 29 800), 425 (*ε* = 399 100), 551 (*ε* = 17 400), 591 (*ε* = 4300).

### Zn-PTetraPyr

Zn-PTetraBr (13 mg, 0.013 mmol), 1-pinacol pyreneboronate (Pyr-Bpin) (19 mg, 0.058 mmol), [PdCl_2_(dppf)] (7.6 mg, 0.010 mmol), and ^*t*^BuONa (15 mg, 0.15 mmol) were placed in a sealed vessel specifically designed for microwave irradiation, which was placed inside a two-necked round-bottom flask in order to keep the system under an inert atmosphere. 1.4 mL of dry toluene and 36 μL of pyridine were added. The mixture was stirred inside the microwave reactor at 130 °C for 60 min. After cooling, the solvent was removed under vacuum, and the crude was purified by column chromatography on silica gel using hexane/AcOEt gradient elution (3 : 1–2 : 1–1 : 1) and CHCl_3_ to finally give Zn-PTetraPyr as a purple solid (15 mg, 80% yield). ^1^H NMR (500 MHz, chloroform-*d*): *δ* 9.33 (s, 8H, H_2_), 8.70 (d, *J* = 9.2 Hz, 4H, H_10_), 8.54 (d, *J* = 7.7 Hz, 8H, H_6_), 8.44 (d, *J* = 7.7 Hz, 4H, H_17_), 8.42 (d, *J* = 7.7 Hz, 4H, H_18_), 8.318.26 (m, 12H, H_11_ + H_12_ + H_14_), 8.24 (d, *J* = 8.9 Hz, 4H, H_16_), 8.20 (d, *J* = 8.9 Hz, 4H, H_15_), 8.14–8.08 (m, 12H, H_7_ + H_13_). ^13^C{^1^H} NMR (126 MHz, chloroform-*d*): *δ* 150.36 (C_3_), 142.12 (C_5_), 140.22 (C_8_), 137.61 (C_9_), 134.70 (C_6_), 132.19 (C_2_), 131.56 (C_21_), 131.43 (C_q_), 131.08 (C_20_), 130.79 (C_22_), 128.76 (C_7_), 128.05 (C_19_), 127.98 (C_18_), 127.75 (C_11_), 127.52 (C_15_ + C_16_), 126.07 (C_13_), 125.46 (C_10_), 125.20 (C_12_ or C_14_), 125.03 (C_23_), 124.93 (C_12_ or C_14_), 124.88 (C_17_), 120.90 (C_4_). HR-MS (MALDI-TOF): *m*/*z* for C_108_H_60_N_4_Zn [M]^+^ calcd: 1476.4115, found: 1476.4116 (0.1 ppm error). UV/vis (toluene): *λ* 345 (*ε* = 72 400), 404 (*ε* = 28 100), 430 (*ε* = 379 300), 552 (*ε* = 17 600), 592 (*ε* = 6400).

### 2H-PTetraPyr

Zn-PTetraPyr (12 mg, 0.0081 mmol) was dissolved in 1.5 mL of CHCl_3_, and 1 mL of CF_3_COOH was then added. The initial deep red solution turned green after the addition of the acid. The mixture was stirred at room temperature for 4 h. Subsequently, it was diluted with 10 mL of CHCl_3_, and a saturated solution of Na_2_CO_3_ in water was added portion-wise with vigorous stirring until the evolution of gas ceased and the organic layer had become deep red again. This layer was separated from the aqueous phase and washed three times with water (10 mL). The organic layer was removed under low pressure to give the purple solid 2H-PTetraPyr (11 mg, 96% yield). ^1^H NMR (500 MHz, chloroform-*d*): *δ* 9.23 (s, 8H, H_2_), 8.68 (d, *J* = 9.2 Hz, 4H, H_10_), 8.54 (d, *J* = 7.7 Hz, 8H, H_6_), 8.44 (d, *J* = 7.7 Hz, 4H, H_17_), 8.41 (d, *J* = 7.7 Hz, 4H, H_18_), 8.32–8.25 (m, 12H, H_11_ + H_12_ + H_14_), 8.24 (d, *J* = 8.8 Hz, 4H, H_16_), 8.20 (d, *J* = 8.8 Hz, 4H, H_15_), 8.15–8.08 (m, 12H, H_7_ + H_13_), −2.48 (br, 2H, H_1_). ^13^C{^1^H} NMR (126 MHz, chloroform-*d*): *δ* 141.18 (C_5_), 140.66 (C_8_), 137.44 (C_9_), 134.84 (C_6_), 131.59 (C_21_), 131.32 (C_q_), 131.11 (C_20_), 130.88 (C_22_), 129.03 (C_7_), 128.76 (C_19_), 127.96 (C_18_), 127.83 (C_11_), 127.63 (C_15_), 127.53 (C_16_), 126.13 (C_13_), 125.40 (C_10_), 125.29 (C_12_ or C_14_), 125.23 (C_23_), 125.05 (C_24_), 125.01 (C_12_ or C_14_), 124.91 (C_17_), 120.15 (C_4_). HR-MS (MALDI-TOF): *m*/*z* for C_108_H_62_N_4_ [M]^+^ calcd: 1414.4980, found: 1414.5022 (4.2 ppm error). UV/vis (toluene): *λ* 349 (*ε* = 16 900), 411 (*ε* = 16 000), 426 (*ε* = 53 100), 518 (*ε* = 8600), 552 (*ε* = 8000), 595 (*ε* = 7000), 656 (*ε* = 6500).

### Zn-PTetraCor

Zn-PTetraBr (9 mg, 0.0090 mmol), 1-pinacol corannuleneboronate (Cor-Bpin) (17 mg, 0.045 mmol), [PdCl_2_(dppf)] (5.8 mg, 0.0079 mmol), and ^*t*^BuONa (11 mg, 0.11 mmol) were mixed in a sealed vessel specifically designed for microwave irradiation, which was placed inside a two-necked round-bottom flask in order to keep the system under an inert atmosphere. 1.4 mL of dry toluene and 36 μL of pyridine were added. The mixture was stirred inside the microwave reactor at 130 °C for 60 min. After cooling, the solvent was removed under vacuum and the crude was purified by column chromatography on silica gel using hexane/AcOEt gradient elution (3 : 1–2 : 1–1 : 1) and CHCl_3_ to finally give the purple solid Zn-PTetraCor (10 mg, 70% yield). ^1^H NMR (500 MHz, chloroform-*d*): *δ* 9.26 (s, 8H, H_2_), 8.50 (d, *J* = 8.0 Hz, 8H, H_6_), 8.29 (s, 4H, H_10_), 8.26 (d, *J* = 8.9 Hz, 4H, H_18_), 8.25 (d, *J* = 8.0 Hz, 8H, H_7_), 8.01 (d, *J* = 8.6 Hz, 4H, H_11_), 7.98 (d, *J* = 8.9 Hz, 4H, H_17_), 7.95 (d, *J* = 8.6 Hz, 4H, H_12_), 7.93–7.88 (m, 16H, H_13_ + H_14_ + H_15_ + H_16_). ^13^C{^1^H} NMR (126 MHz, chloroform-*d*) *δ*: 150.35 (C_3_), 142.20 (C_q_-*in*), 141.51 (C_5_-*in*), 138.92 (C_8_-*in*), 136.47 (C_q_), 136.31 (C_q_), 136.00 (C_q_), 135.50 (C_26_), 134.99 (C_6_), 132.27 (C_2_), 130.97 (C_q_), 129.83 (C_19_-*in*), 128.20 (C_7_), 127.63 (C_17_), 127.43 (C_12_), 127.36 (C_*x*_), 127.18 (C_*x*_ + C_18_), 127.09 (C_*x*_ + C_11_), 127.06 (C_*x*_), 126.27 (C_10_). Being C_*x*_ = C_13_ or C_14_ or C_15_ or C_16_. HR-MS (MALDI-TOF): *m*/*z* for C_124_H_60_N_4_Zn [M]^+^ calcd: 1668.4104, found: 1668.4085 (−1.9 ppm error). UV/vis (toluene): *λ* 404 (*ε* = 13 400), 430 (*ε* = 89 300), 553 (*ε* = 9800), 591 (*ε* = 7700).

Note: Zn-PTetraCor could also be prepared, albeit in lower yield (56%), using Zn-PTetraBpin and Cor-Br as the coupling partners (*i.e.*, by a synthetic route similar to route A in Scheme 1).

### 2H-PTetraCor

Zn-PTetraCor (8 mg, 0.0048 mmol) was dissolved in 2.0 mL of CHCl_3_, and 1 mL of CF_3_COOH was then added. The initial deep red solution turned green after the addition of the acid. The mixture was stirred at room temperature for 4 h. Subsequently, it was diluted with 10 mL of CHCl_3_, and a saturated solution of Na_2_CO_3_ in water was added portion-wise with vigorous stirring until the evolution of gas ceased and the organic layer had become deep red again. This layer was separated from the aqueous phase and washed three times with water (10 mL). The organic layer was removed under low pressure to give the purple solid 2H-PTetraCor (7 mg, 91% yield).^1^H NMR (500 MHz, chloroform-*d*): *δ* 9.15 (s, 8H, H_2_), 8.50 (d, *J* = 8.0 Hz, 8H, H_6_), 8.28 (s, 4H, H_10_), 8.26 (d, *J* = 8.0 Hz, 8H, H_7_), 8.25 (d, *J* = 8.8 Hz, 4H, H_18_), 8.01 (d, *J* = 8.6 Hz, 4H, H_11_), 7.98 (d, *J* = 8.8 Hz, 4H, H_17_), 7.95 (d, *J* = 8.6 Hz, 4H, H_12_), 7.93–7.88 (m, 16H, H_13_ + H_14_ + H_15_ + H_16_), −2.52 (br, 2H, H_1_). ^13^C{^1^H} NMR (126 MHz, chloroform-*d*): *δ* 141.45 (C_5_), 139.2 (C_8_-*in*), 136.50 (C_23_ or C_25_), 136.33 (C_q_), 136.00 (C_q_-*in*), 135.90 (C_q_-*in*), 135.53 (C_26_), 135.16 (C_6_), 130.97 (C_q_), 130.88 (C_q_), 129.75 (C_19_), 128.35 (C_7_), 127.67 (C_17_), 127.53 (C_12_), 127.40 (C_*x*_), 127.20 (C_*x*_ + C_18_), 127.12 (C_*x*_ + C_11_), 127.02 (C_*x*_), 126.34 (C_10_), 120.00 (C_4_-*in*). Being C_*x*_ = C_13_ or C_14_ or C_15_ or C_16_. HR-MS (MALDI-TOF): *m*/*z* for C_124_H_62_N_4_ [M]^+^ calcd: 1606.4969, found: 1606.4994 (2.5 ppm error). UV/vis (toluene): *λ* 404 (*ε* = 12 200), 426 (*ε* = 38 500), 518 (*ε* = 8100), 555 (*ε* = 7600), 595 (*ε* = 7100), 651 (*ε* = 7100).

## Conflicts of interest

There are no conflicts to declare.

## Supplementary Material

RA-010-D0RA07407A-s001
