# Supplementary material for: Porphyrin-based systems containing polyaromatic fragments: decoupling the synergistic effects in aromatic-porphyrin-fullerene systems
Source: RSC Adv. 2020 Oct 1;10(59):36164–73. doi: 10.1039/d0ra07407a (PMC9056955; doi:10.1039/d0ra07407a)
Supplement: RA-010-D0RA07407A-s001 [file RA-010-D0RA07407A-s001.pdf]

Electronic Supplementary Material (ESI) for RSC Advances.
This journal is © The Royal Society of Chemistry 2020

Supporting Information

Decoupling the synergistic effects in aromatic-porphyrin-fullerene systems

Sergio Ferrero, Héctor Barbero, Daniel Miguel, Raúl García-Rodríguez* and Celedonio M. Álvarez*

Table of Contents

General atom numbering 2
NMR spectra 3
  2H-PTetraBr 3
  Zn-PTetraBr 5
  2H-PTetraBpin 7
  Zn-PTetraBpin 10
  Zn-PTetraPyr 13
  2H-PTetraPyr 16
  Zn-PTetraCor 19
  2H-PTetraCor 22
UV/Vis spectra 25
MS spectra 27
Complexation measurements 34
Computational methods 40
References 48

General atom numbering

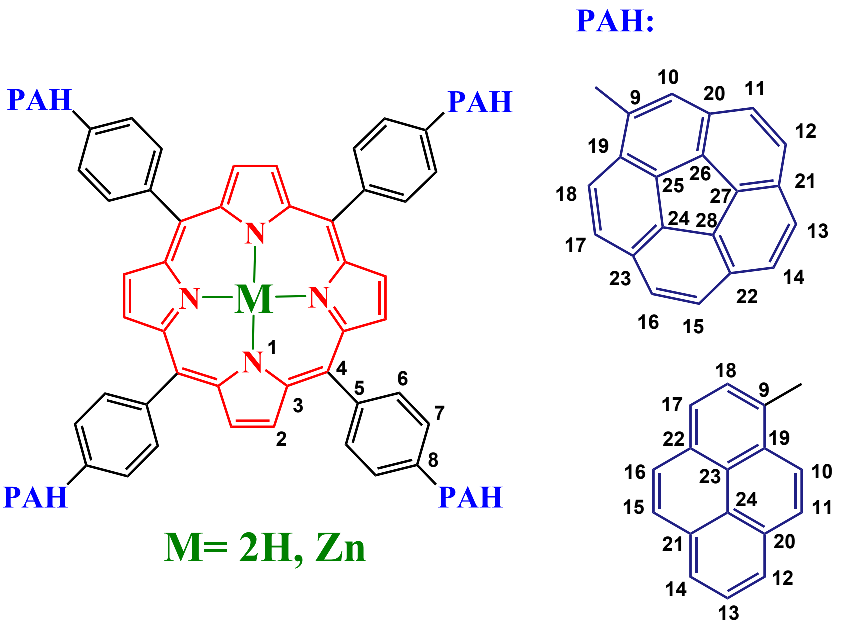

NMR spectra

2H-PTetraBr

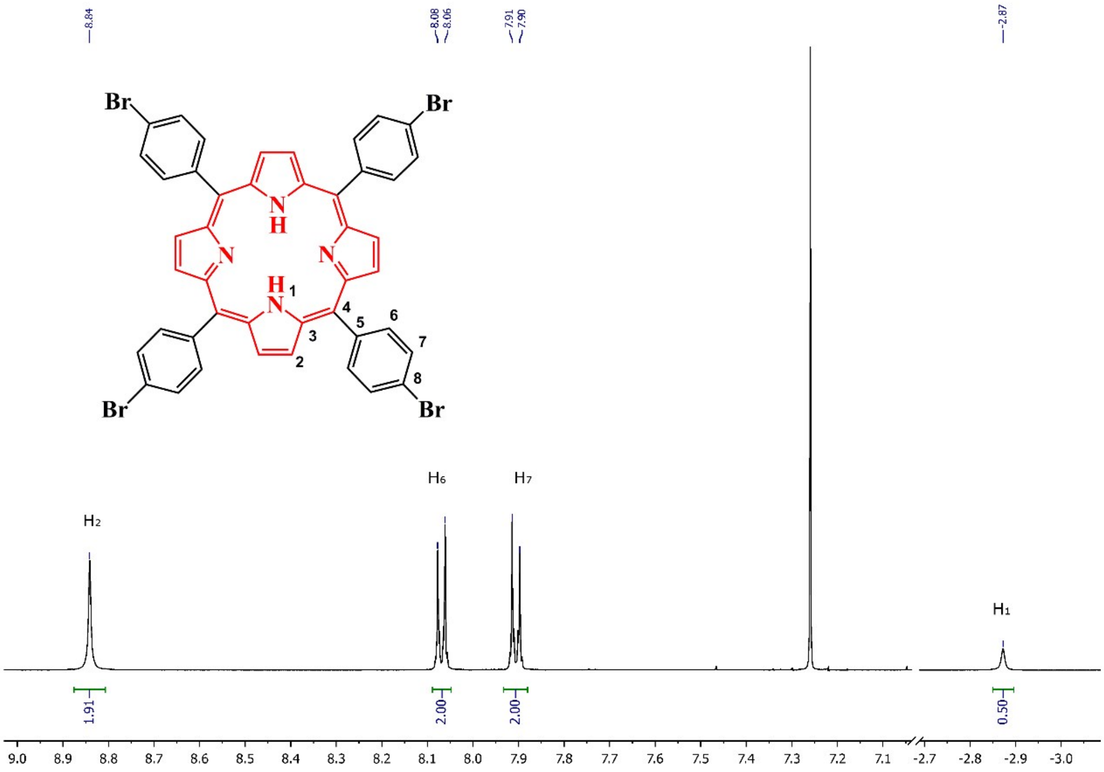

Figure S1: ^{1}H NMR (500 MHz, $CDCl_3$) spectrum of **2H-PTetraBr**.

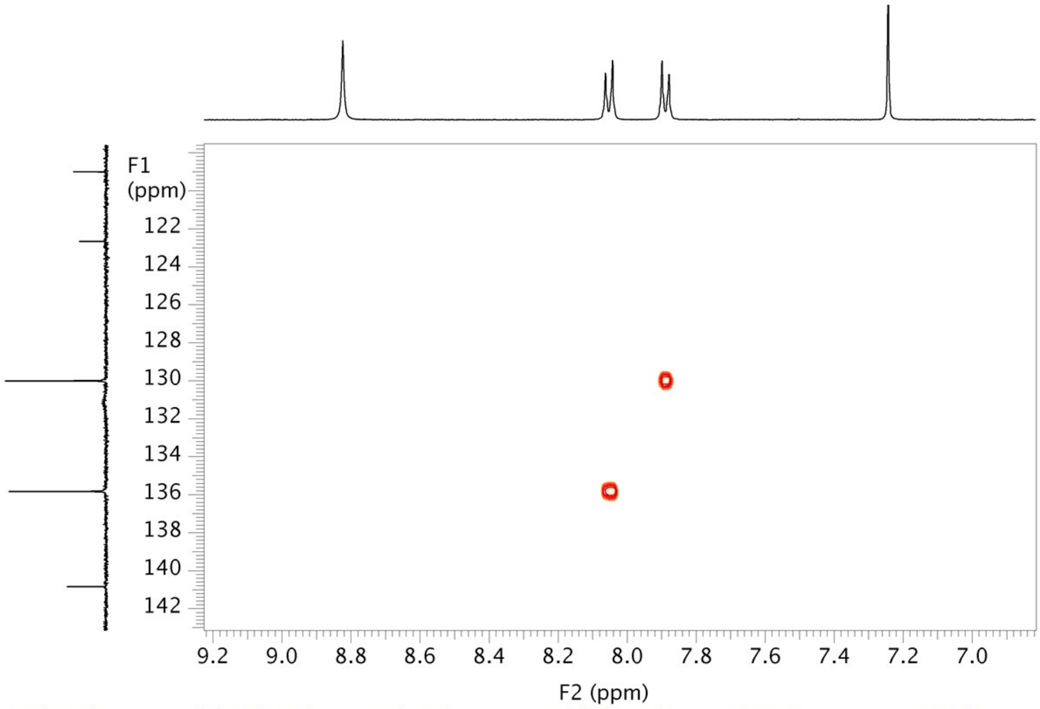

Figure S2: ^{1}H-^{13}C band selective HSQC ($CDCl_3$) of **2H-PTetraBr**.

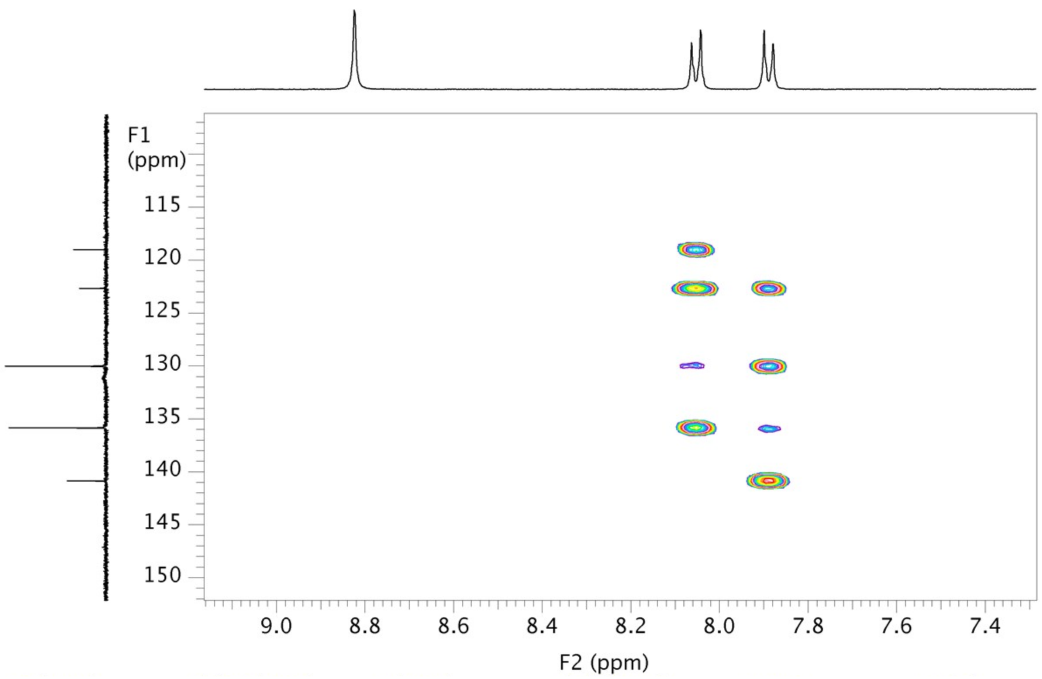

Figure S3: ^{1}H-^{13}C band selective HMBC ($CDCl_3$) of **2H-PTetraBr**.

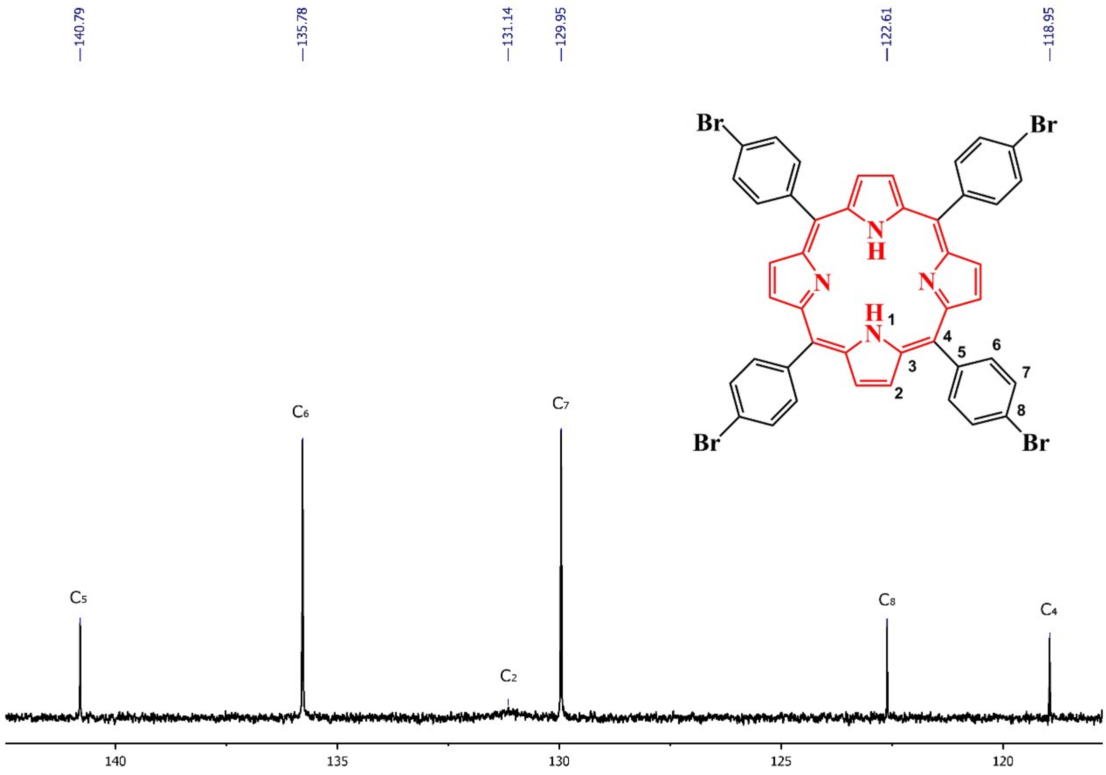

Figure S4: ^{13}C {^{1}H} NMR (101 MHz, $CDCl_3$) spectrum of **2H-PTetraBr**.

Zn-PTetraBr

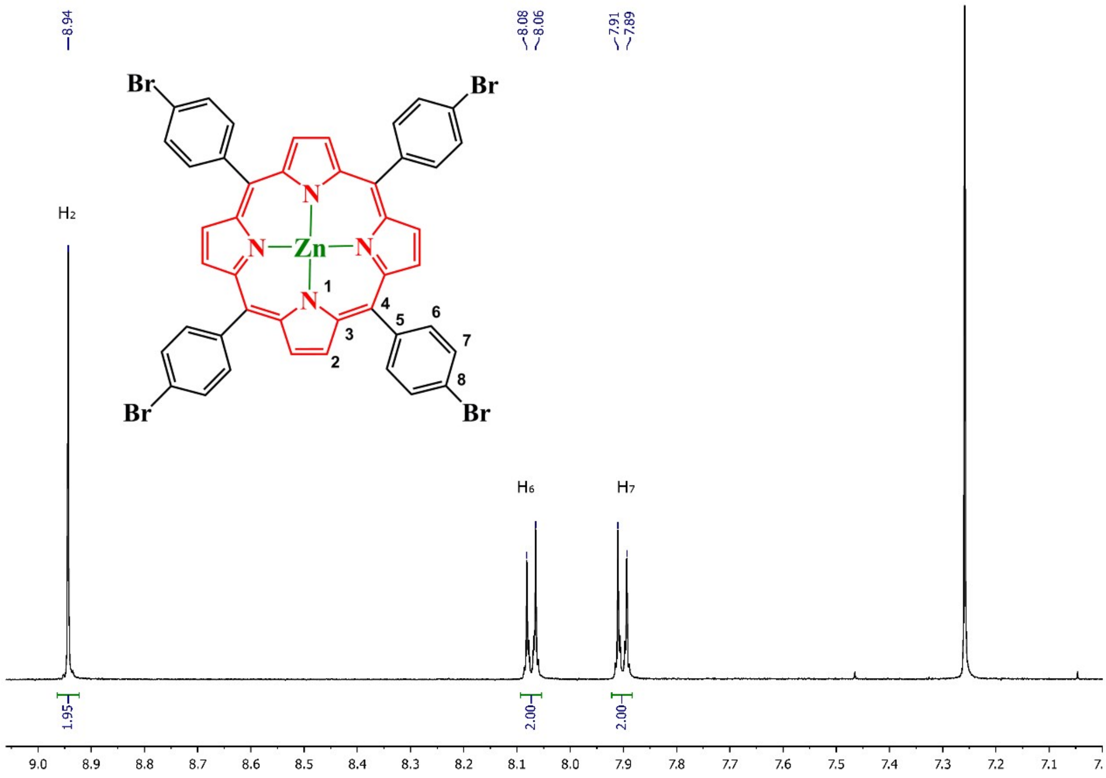

Figure S5: ^{1}H NMR (500 MHz, $CDCl_3$) spectrum of **Zn-PTetraBr**.

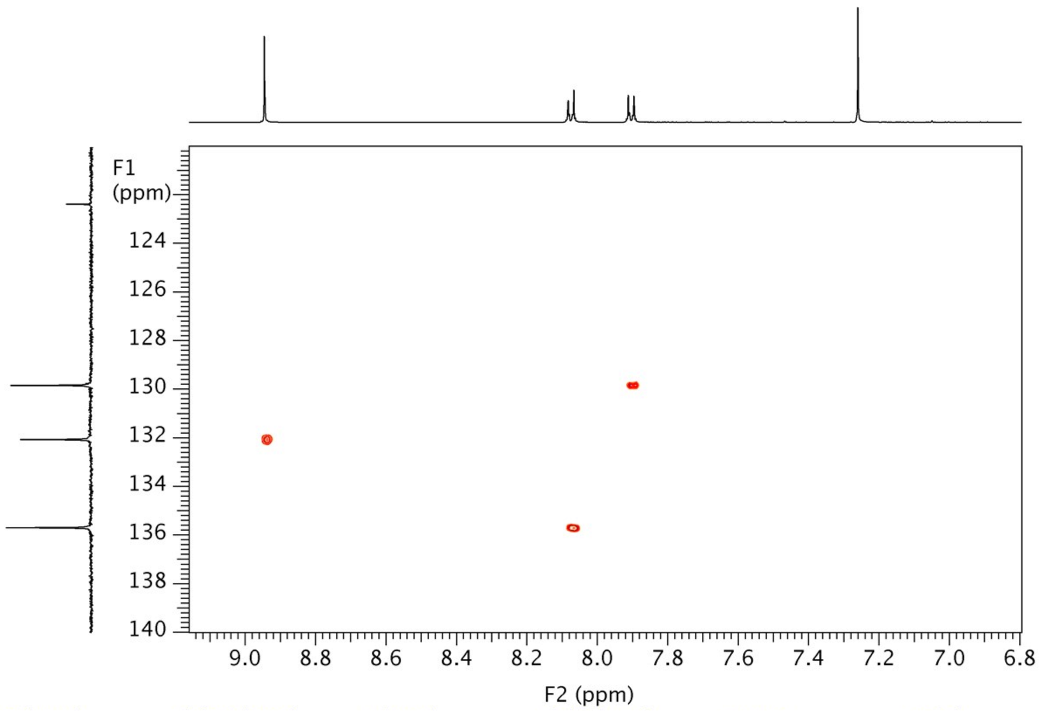

Figure S6: ^{1}H-^{13}C band selective HSQC ($CDCl_3$) of **Zn-PTetraBr**.

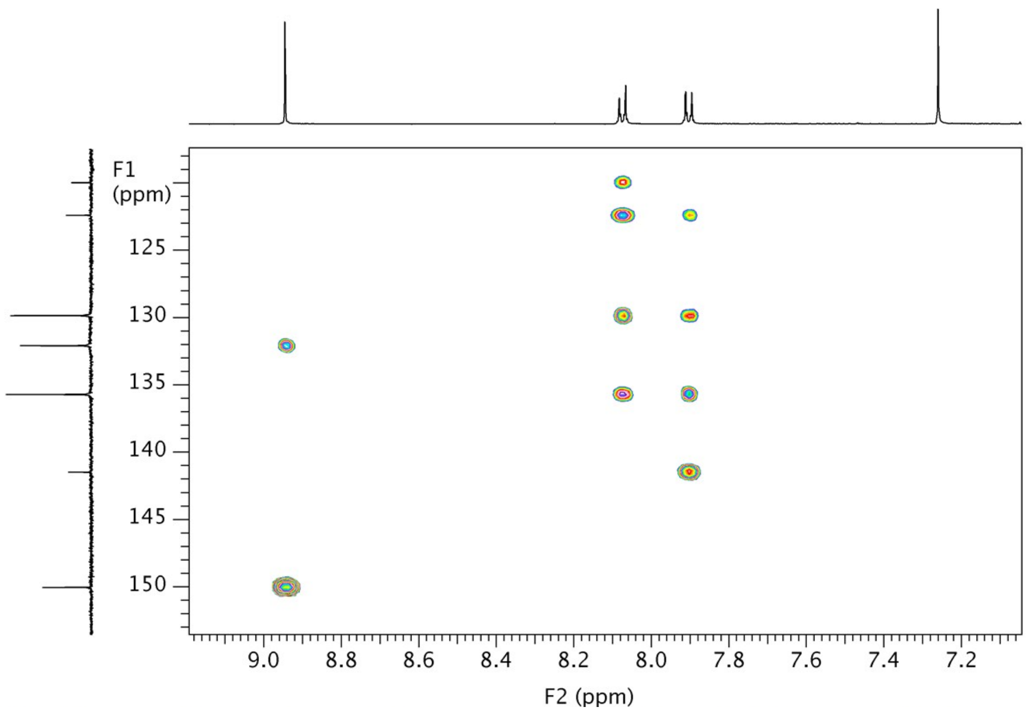

Figure S7: ^{1}H-^{13}C band selective HMBC ($CDCl_3$) of **Zn-PTetraBr**.

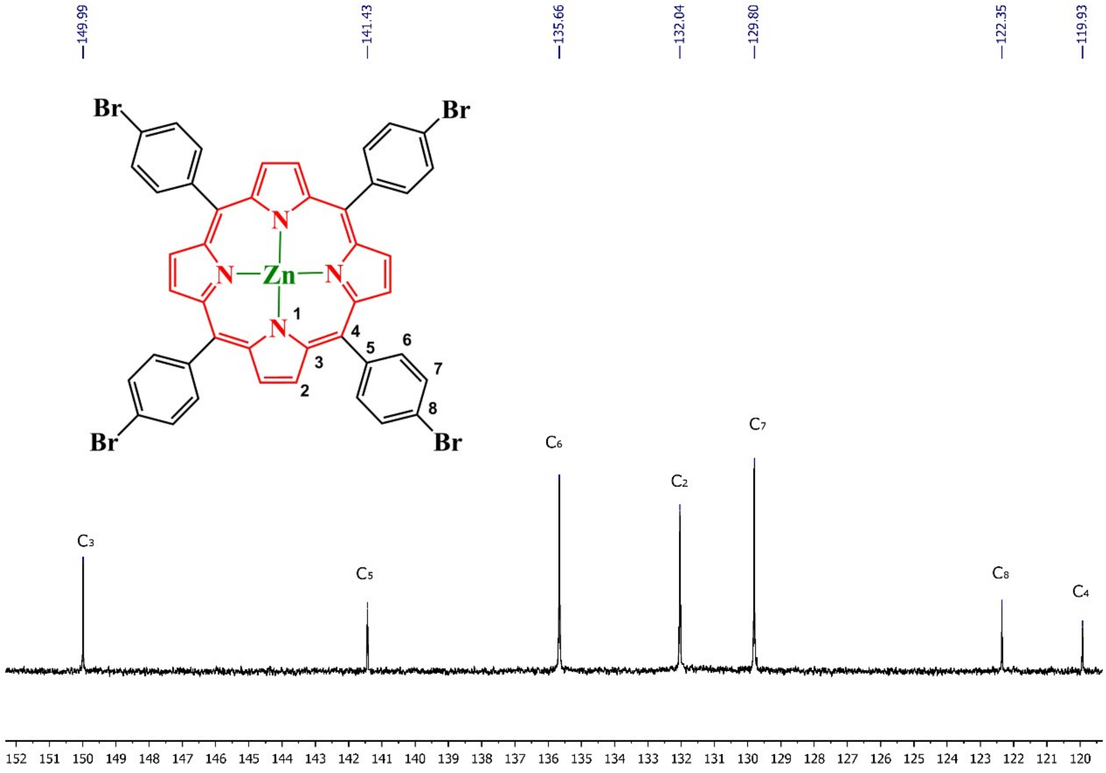

Figure S8: ^{13}C {^{1}H} NMR (101 MHz, $CDCl_3$) spectrum of **Zn-PTetraBr**.

2H-PTetraBpin

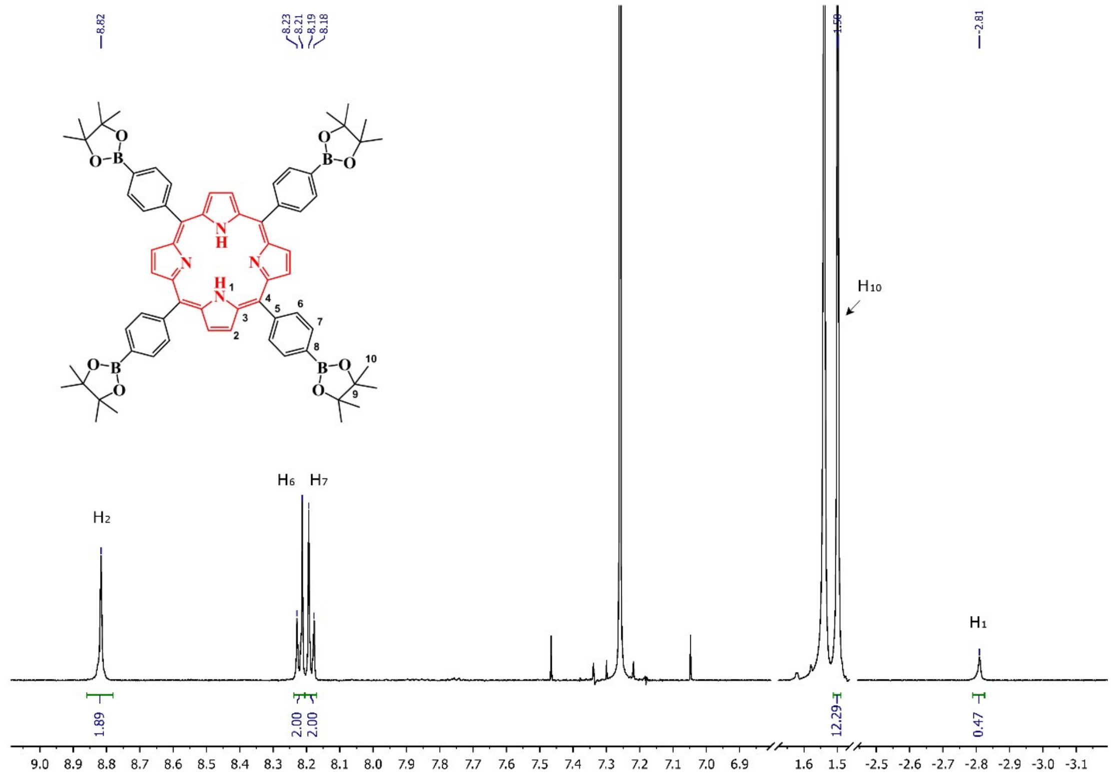

Figure S9: ^{1}H NMR (500 MHz, $CDCl_3$) spectrum of **2H-PTetraBpin**.

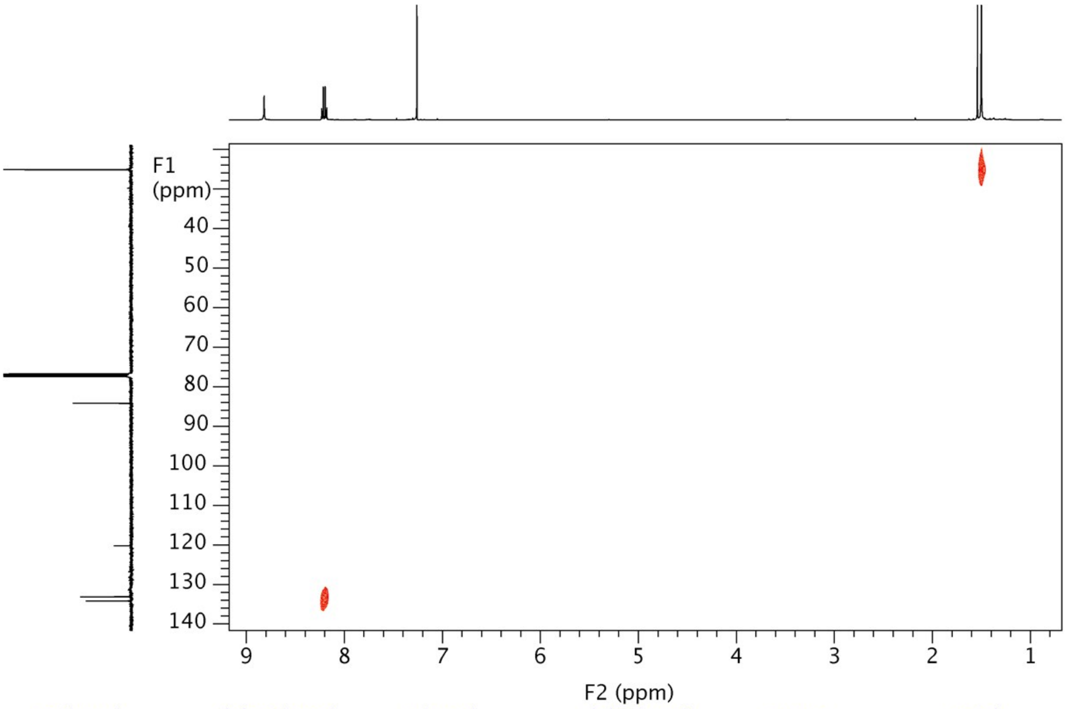

Figure S10: Full ^{1}H-^{13}C HSQC ($CDCl_3$) of **2H-PTetraBpin**.

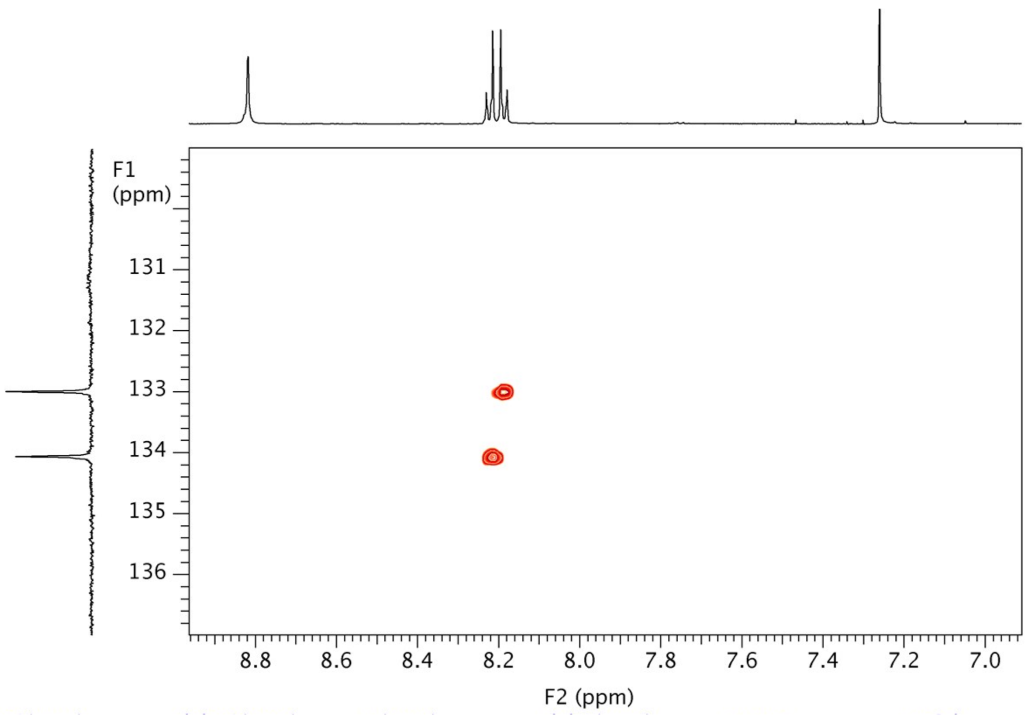

Figure S11: ^{1}H-^{13}C band selective HSQC (expansion of the aromatic region) ($CDCl_3$) of **2H-PTetraBpin**.

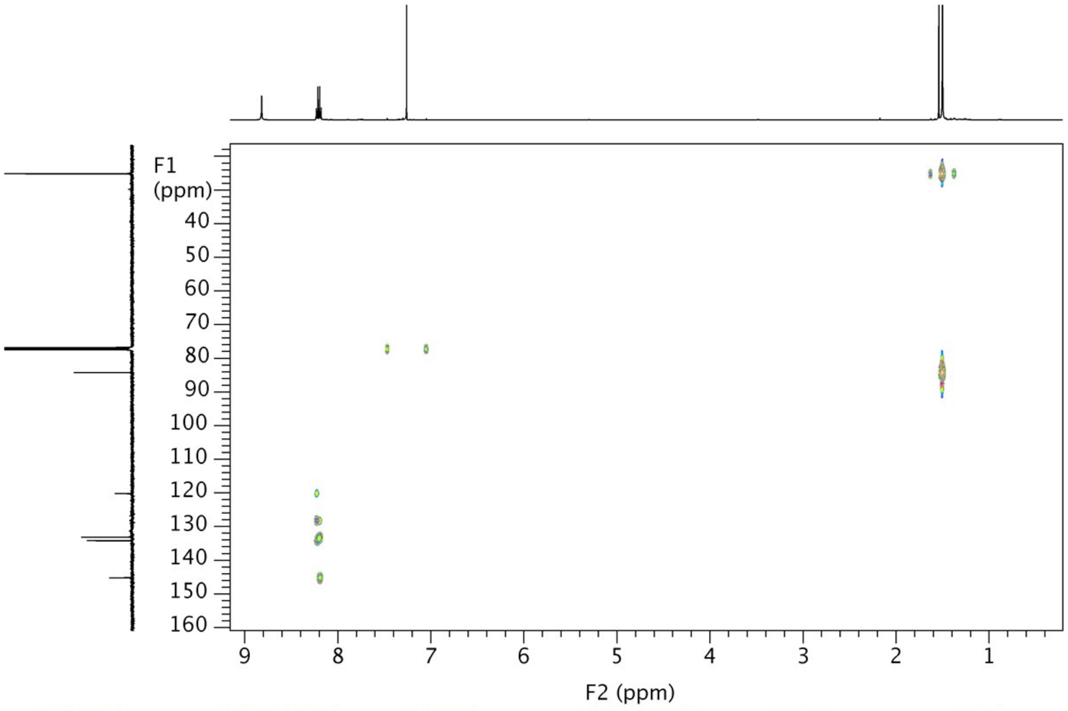

Figure S12: Full ^{1}H-^{13}C HMBC ($CDCl_3$) of **2H-PTetraBpin**.

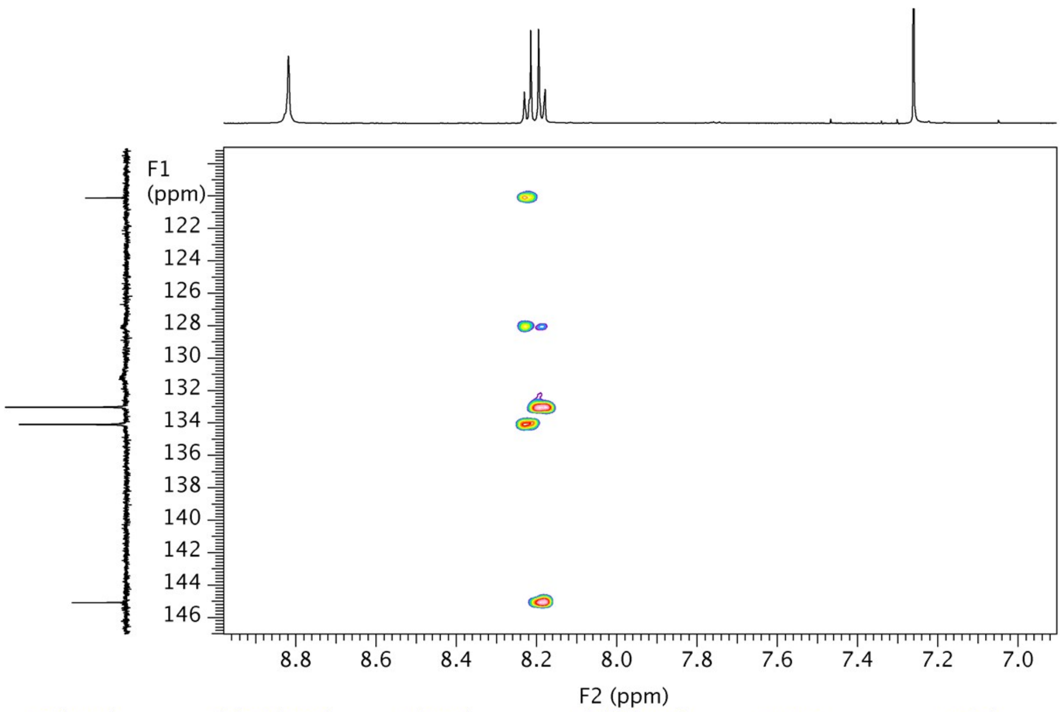

Figure S13: ^{1}H-^{13}C band selective HMBC (expansion of the aromatic region) ($CDCl_3$) of **2H-PTetraBpin**.

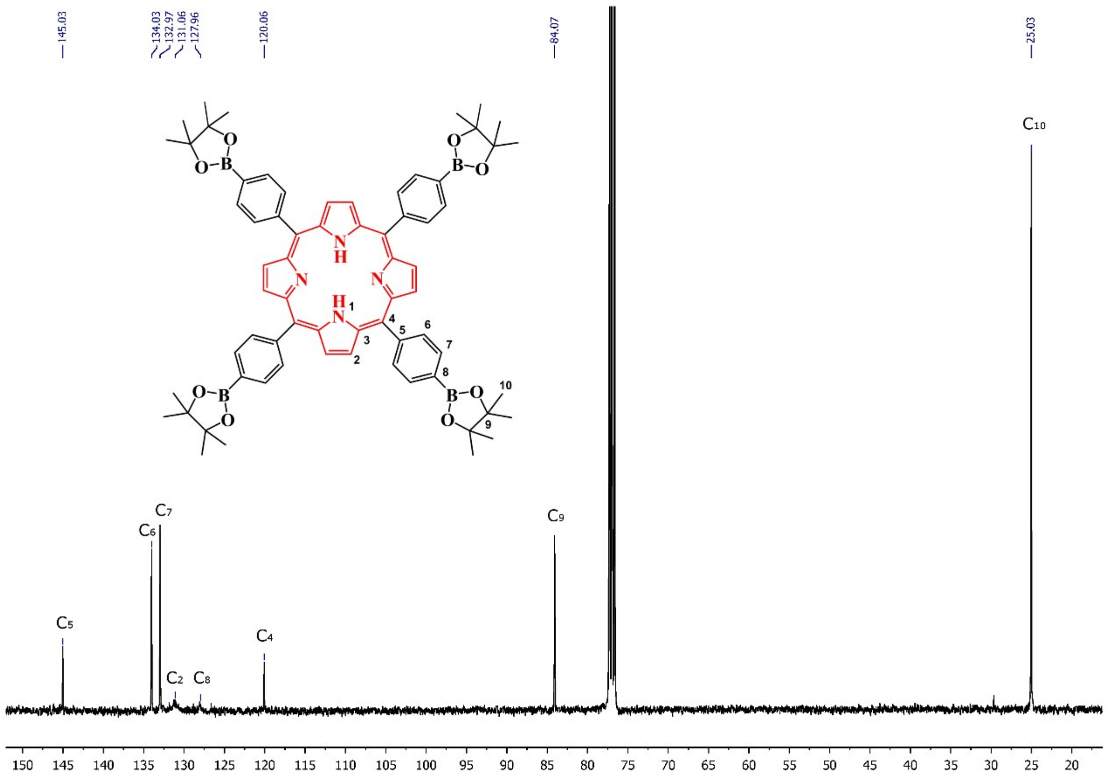

Figure S14: ^{13}C {^{1}H} NMR (101 MHz, $CDCl_3$) spectrum of **2H-PTetraBpin**.

Zn-PTetraBpin

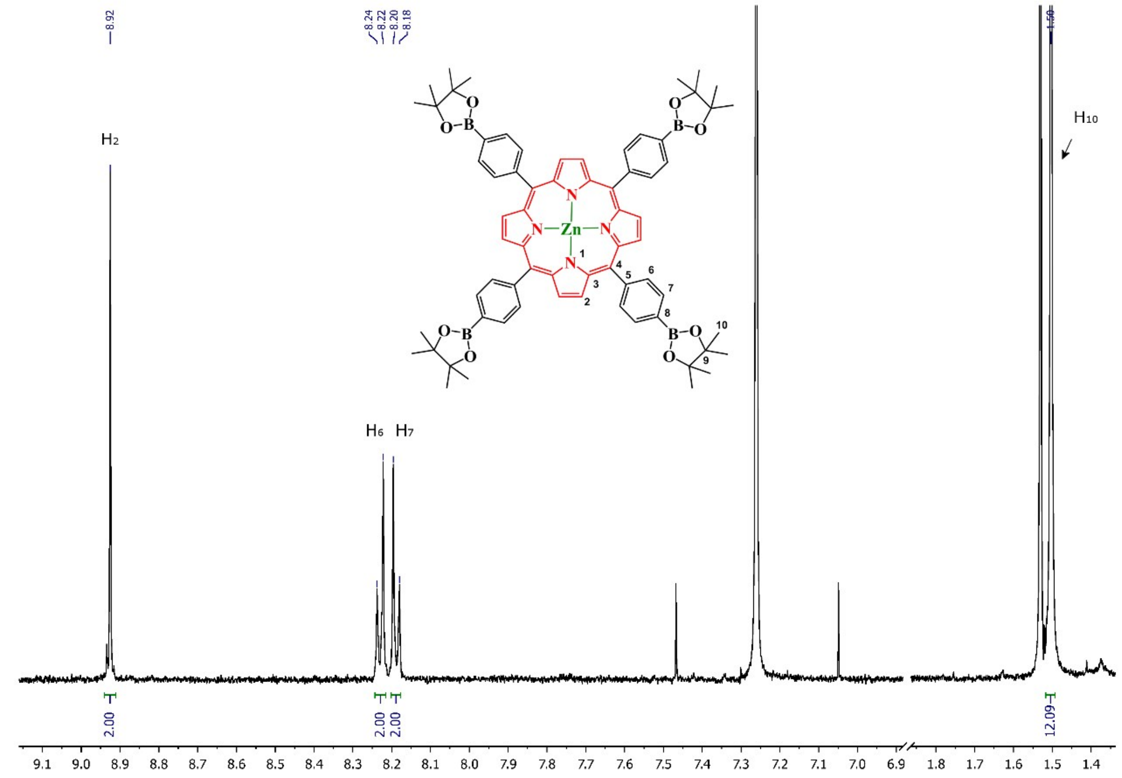

Figure S15: ^{1}H NMR (500 MHz, $CDCl_3$) spectrum of **Zn-PTetraBpin**.

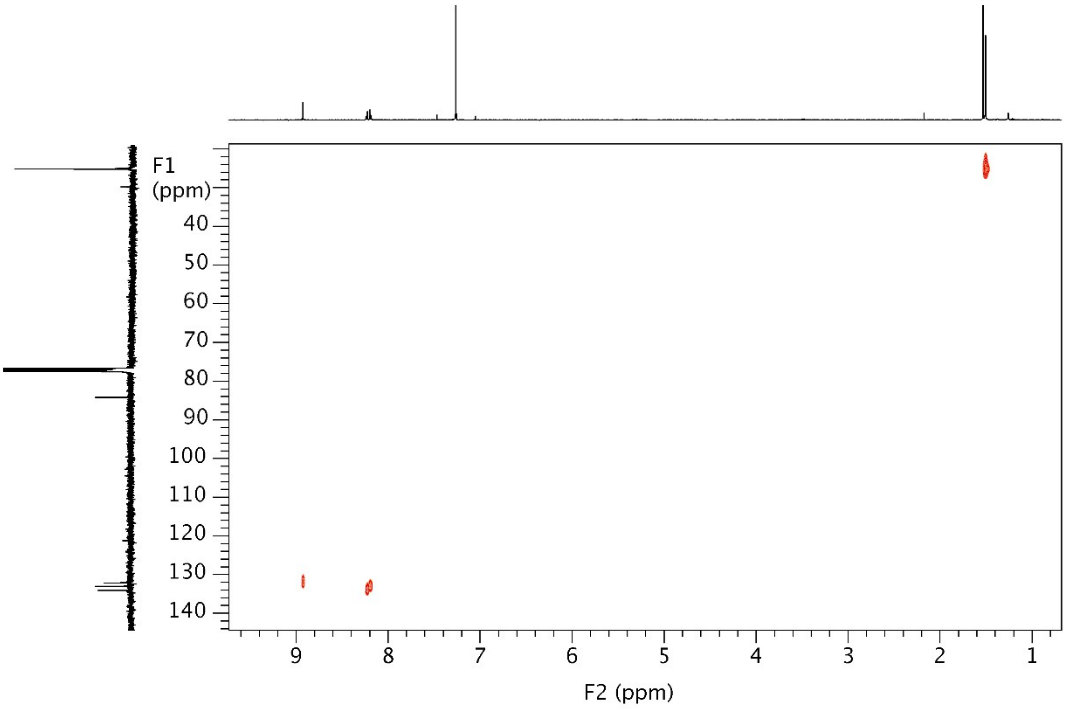

Figure S16: Full ^{1}H-^{13}C HSQC ($CDCl_3$) of **Zn-PTetraBpin**.

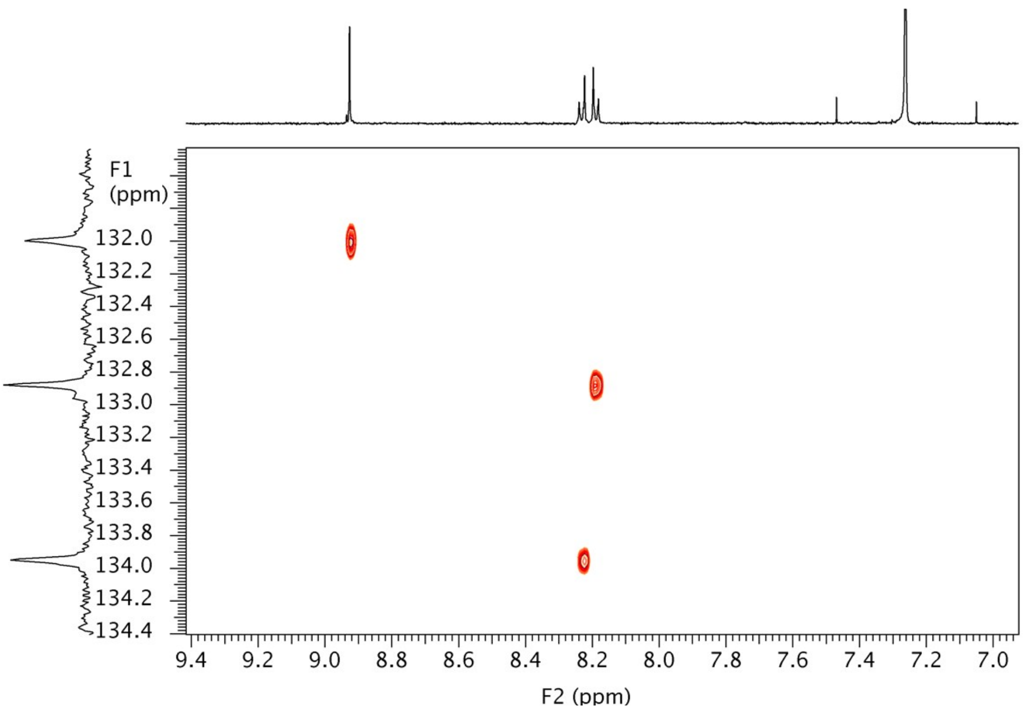

Figure S17: ^{1}H-^{13}C band selective HSQC (expansion of the aromatic region) ($CDCl_3$) of **Zn-PTetraBpin**.

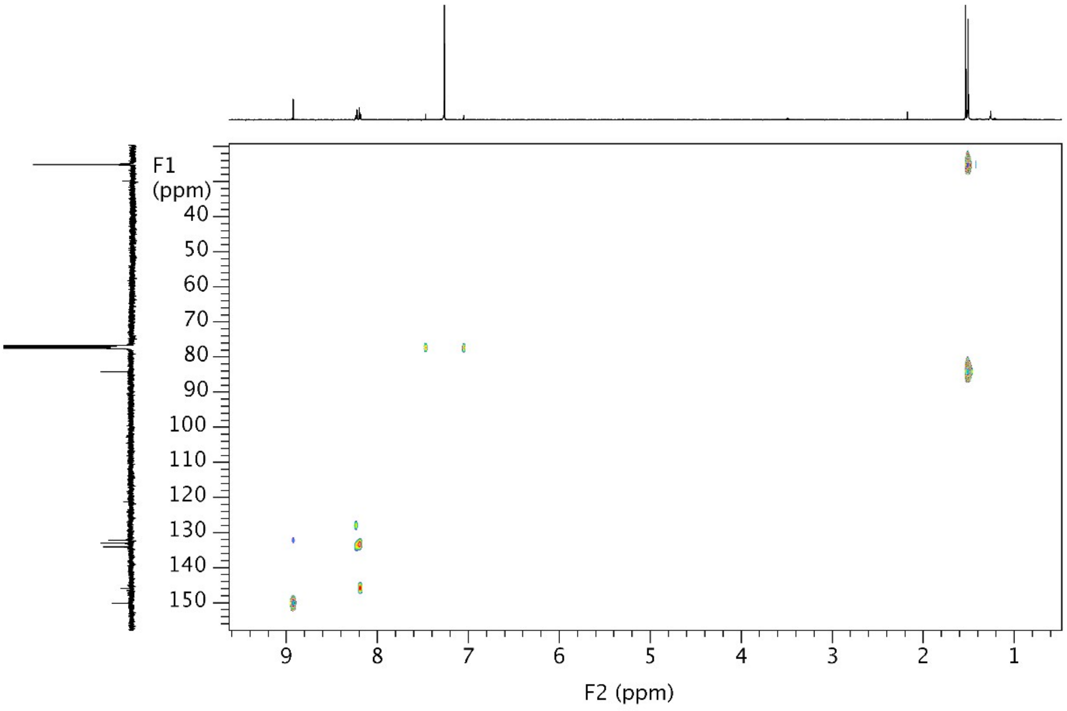

Figure S18: ^{1}H-^{13}C full HMBC ($CDCl_3$) of **Zn-PTetraBpin**.

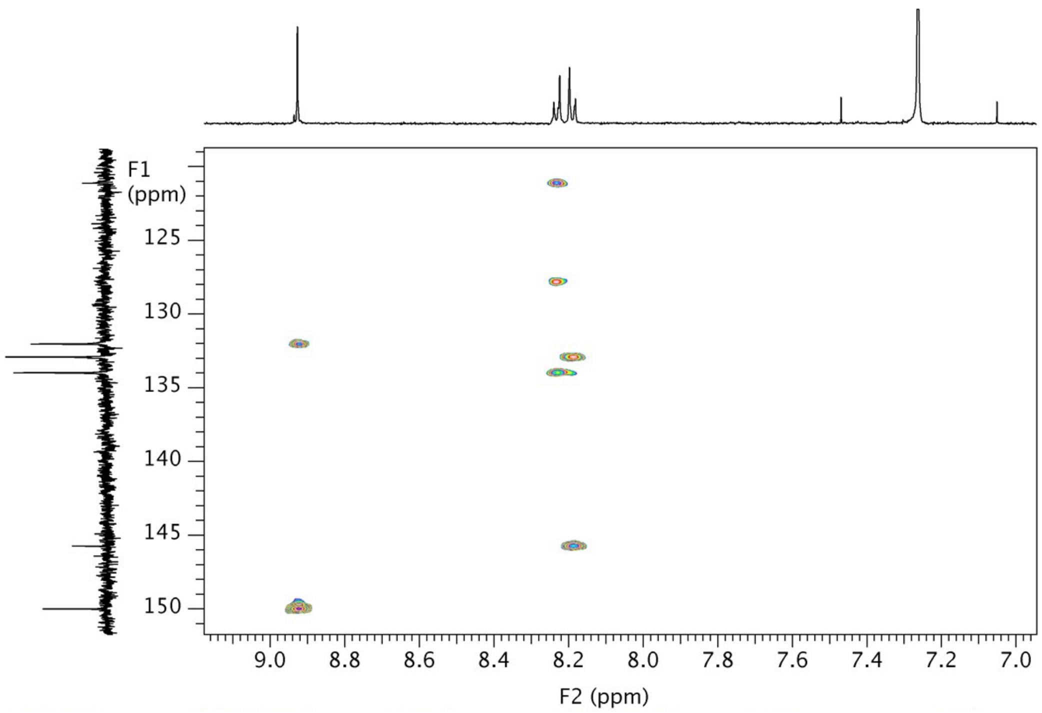

Figure S19: ^{1}H-^{13}C band selective HMBC (expansion of the aromatic region) ($CDCl_3$) of **Zn-PTetraBpin**.

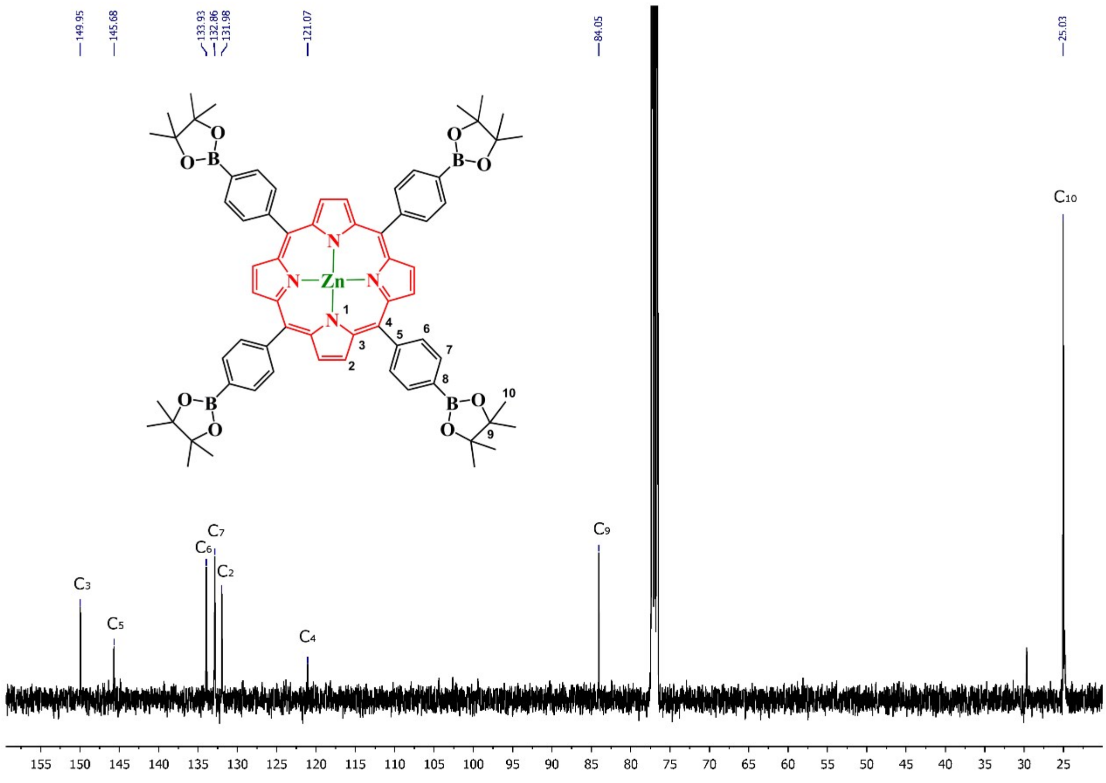

Figure S20: ^{13}C {^{1}H} NMR (101 MHz, $CDCl_3$) spectrum of **Zn-PTetraBpin**.

Zn-PTetraPyr

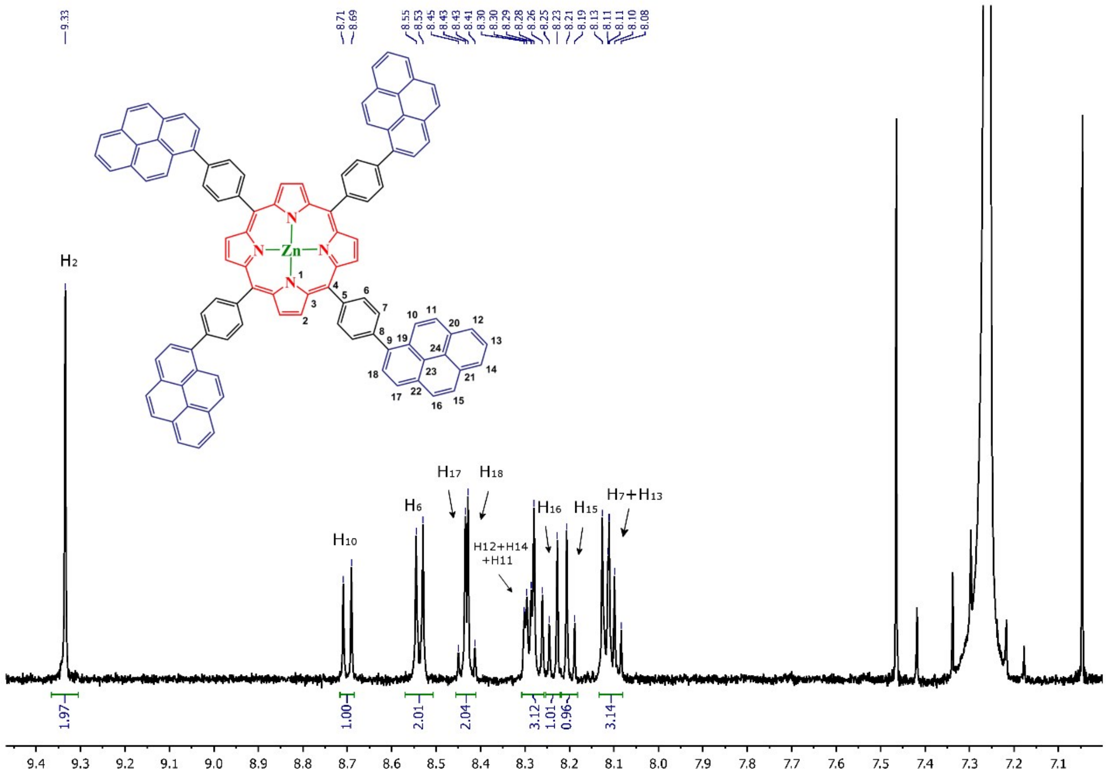

Figure S21: ^{1}H NMR (500 MHz, $CDCl_3$) spectrum of **Zn-PTetraPyr**.

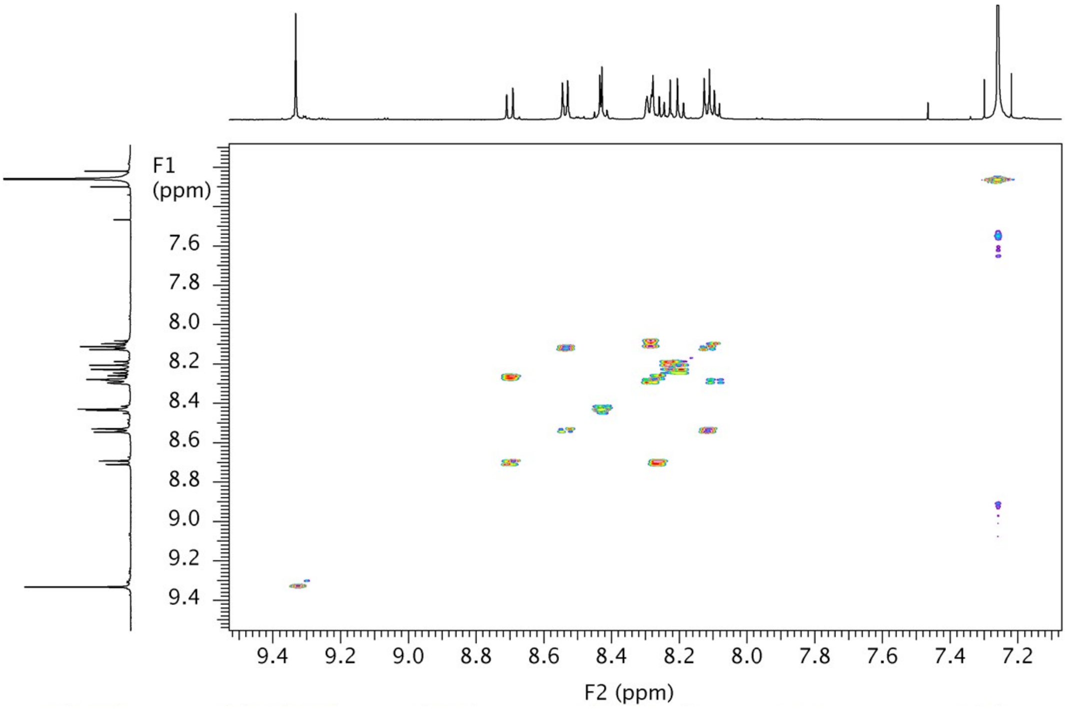

Figure S22: ^{1}H-^{1}H COSY ($CDCl_3$) of **Zn-PTetraPyr**.

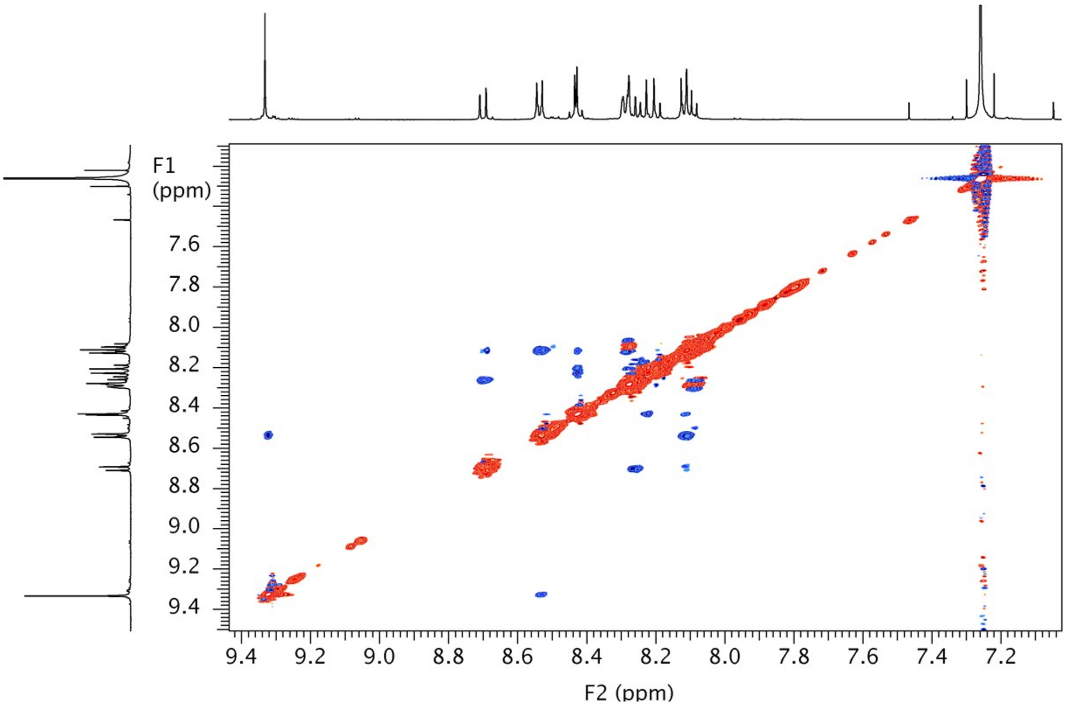

Figure S23: ^{1}H-^{1}H band selective ROESY ($CDCl_3$) of **Zn-PTetraPyr**.

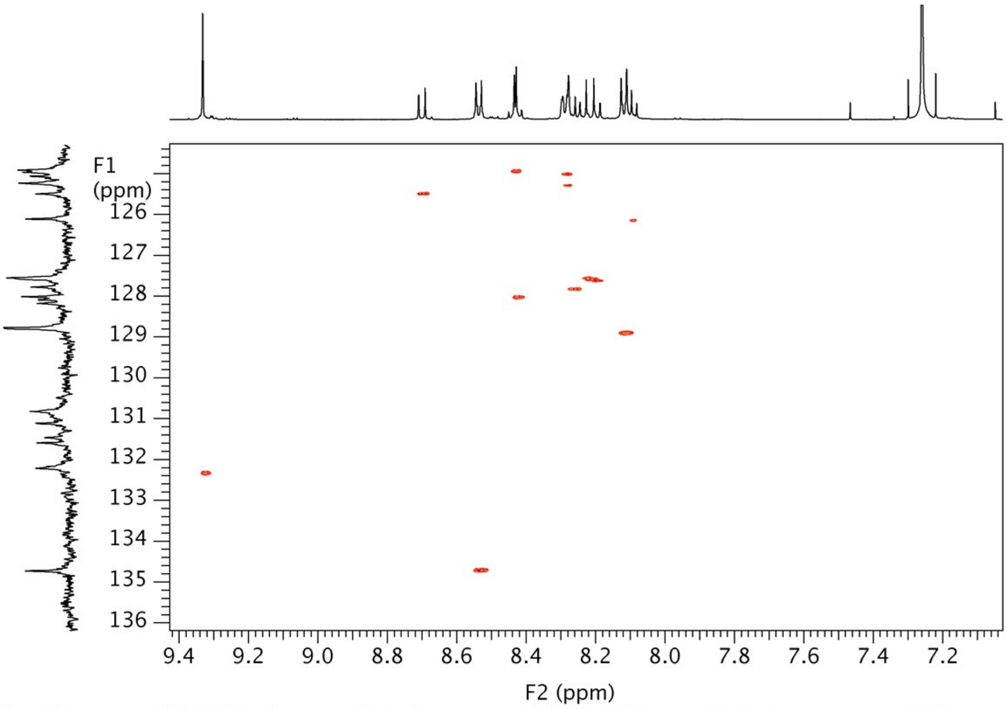

Figure S24: ^{1}H-^{13}C band selective HSQC ($CDCl_3$) of **Zn-PTetraPyr**.

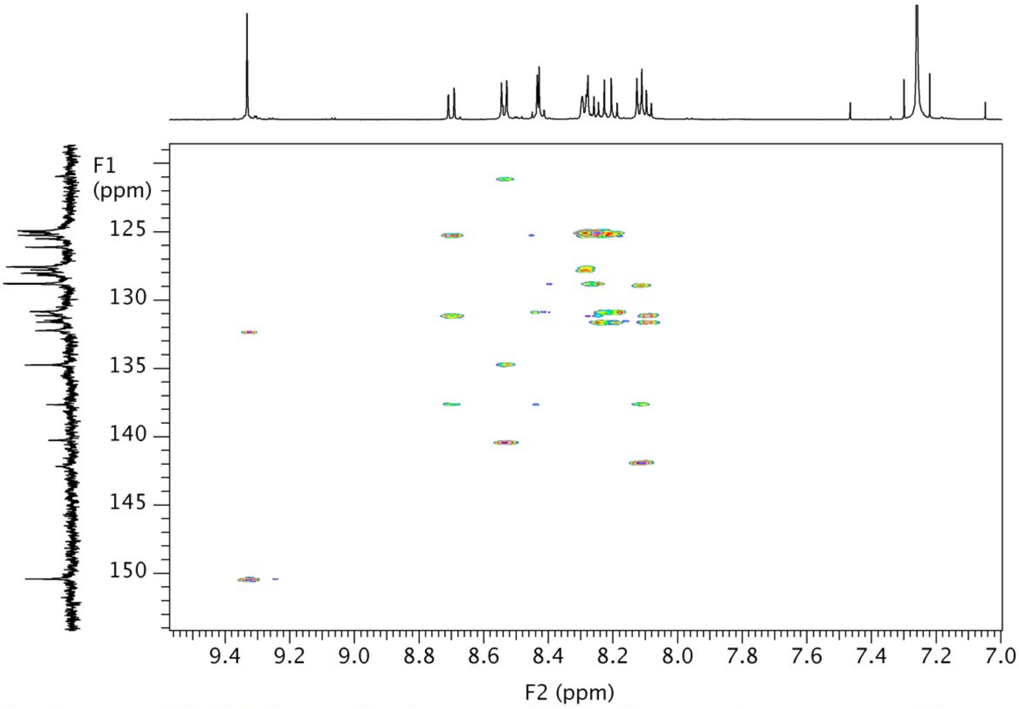

Figure S25: ^{1}H-^{13}C band selective HMBC ($CDCl_3$) of **Zn-PTetraPyr**.

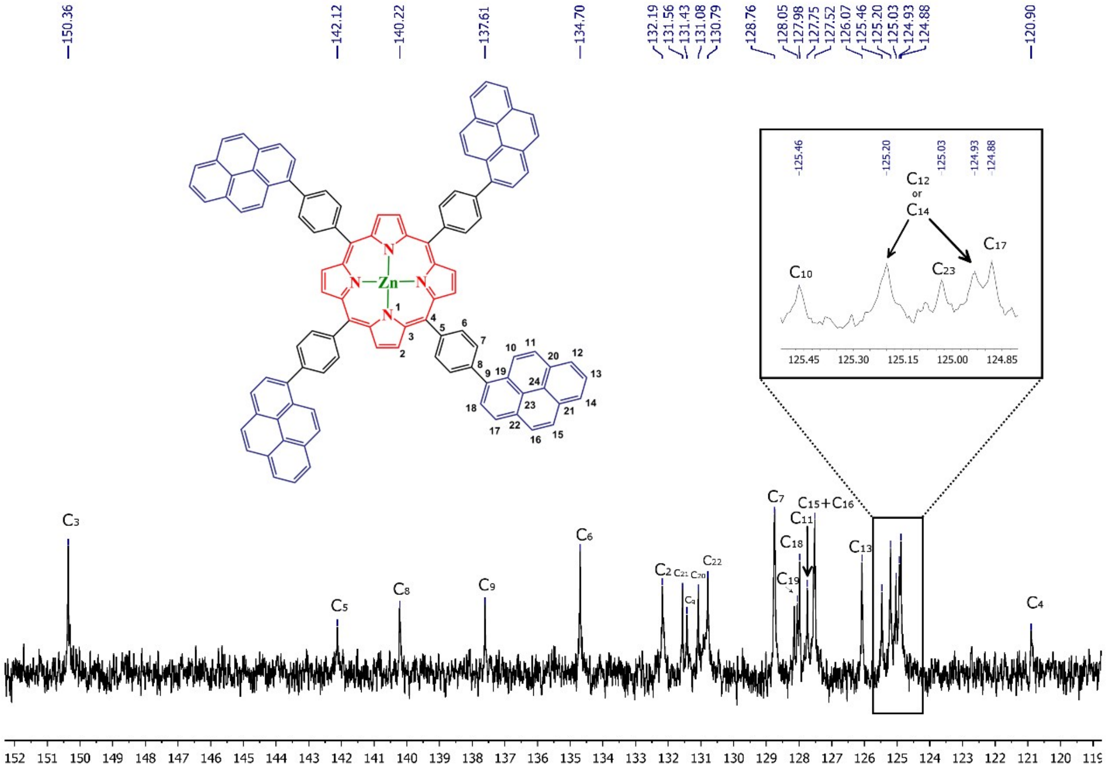

Figure S26: ^{13}C {^{1}H} NMR (126 MHz, $CDCl_3$) spectrum of **Zn-PTetraPyr**.

2H-PTetraPyr

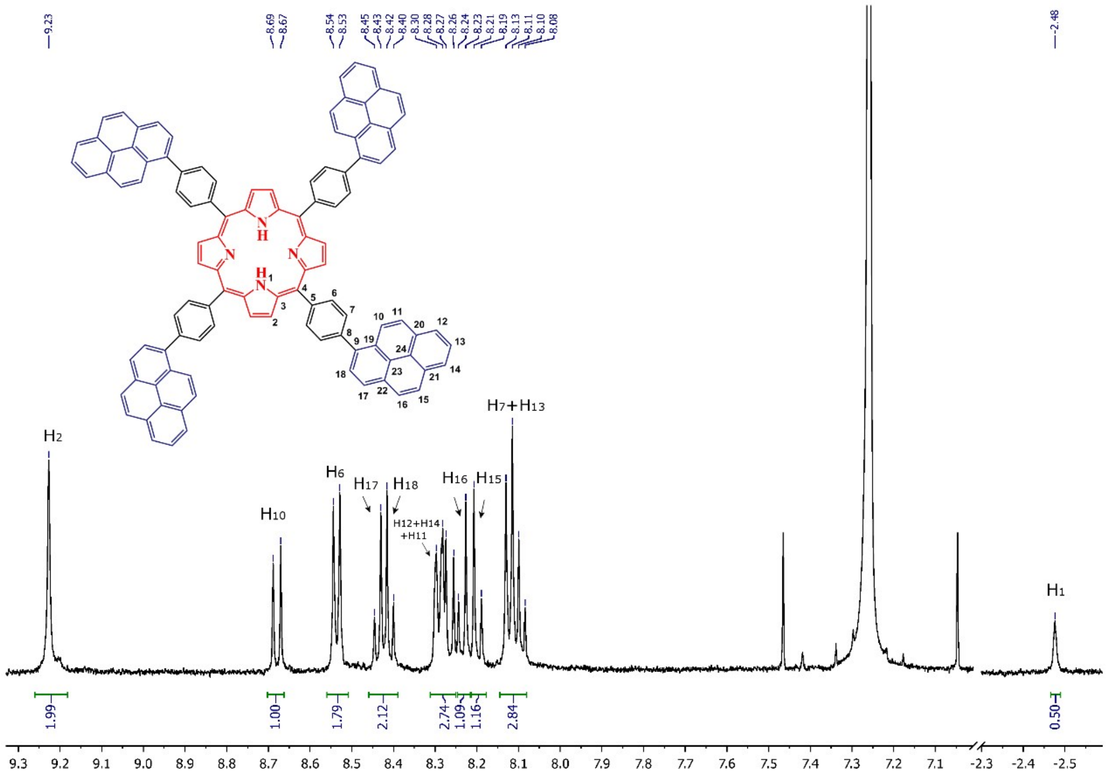

Figure S27: ^{1}H NMR (500 MHz, $CDCl_3$) spectrum of **2H-PTetraPyr**.

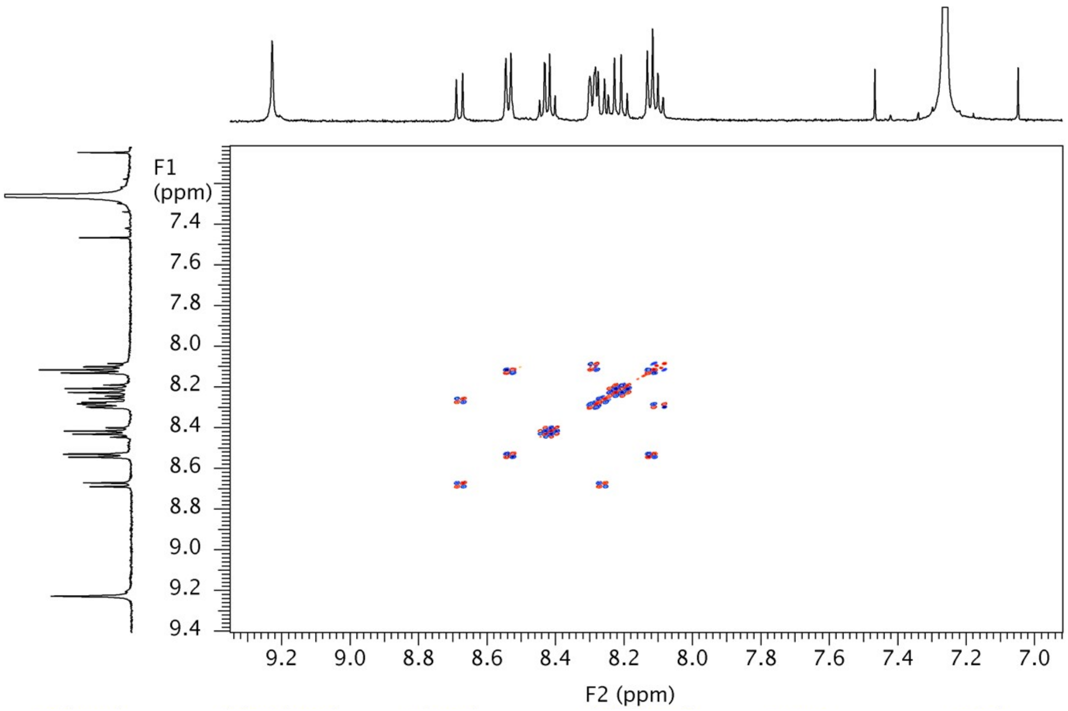

Figure S28: ^{1}H-^{1}H DQFCOSY ($CDCl_3$) of **2H-PTetraPyr**.

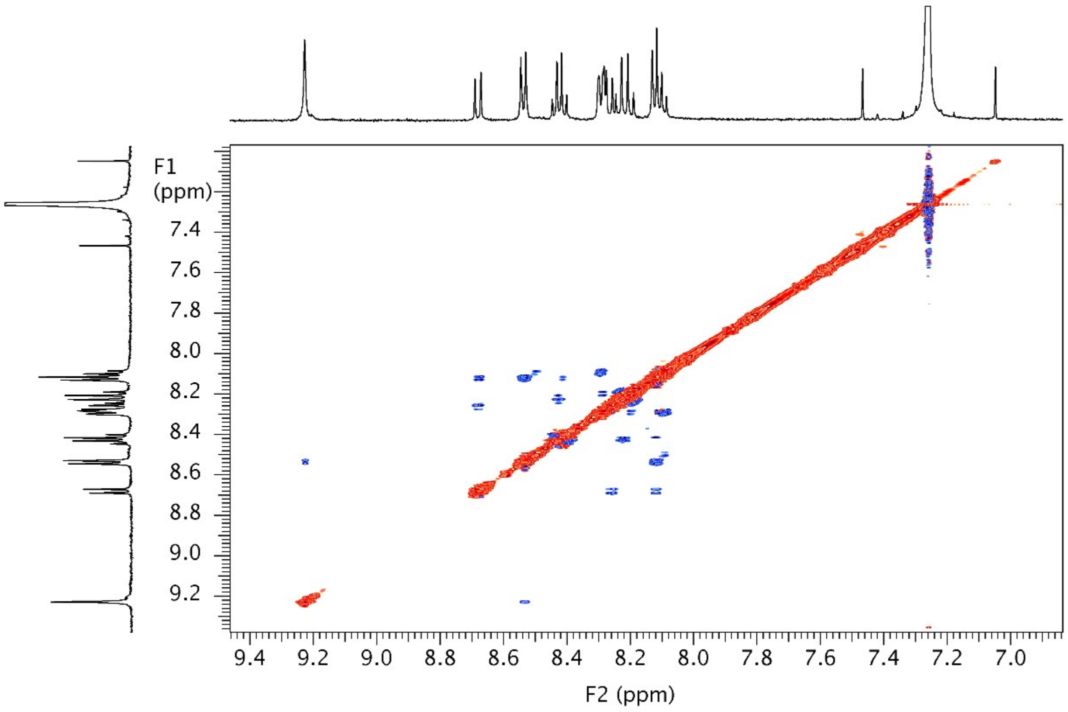

Figure S29: ^{1}H-^{1}H band selective ROESY ($CDCl_3$) of **2H-PTetraPyr**.

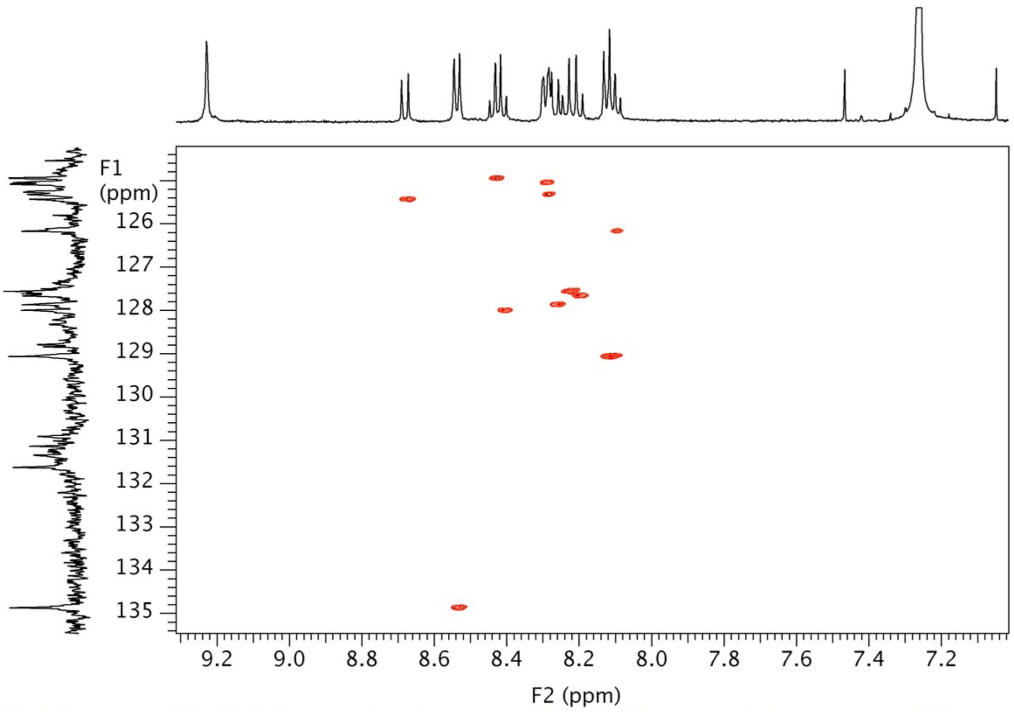

Figure S30: ^{1}H-^{13}C band selective HSQC ($CDCl_3$) of **2H-PTetraPyr**.

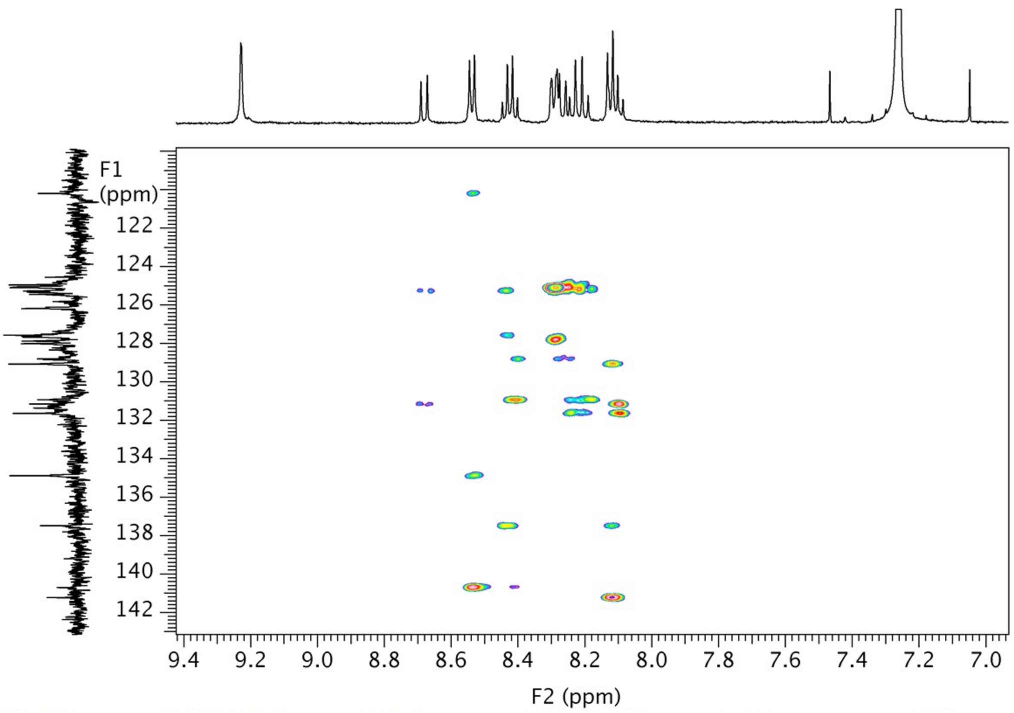

Figure S31: ^{1}H-^{13}C band selective HMBC ($CDCl_3$) of **2H-PTetraPyr**.

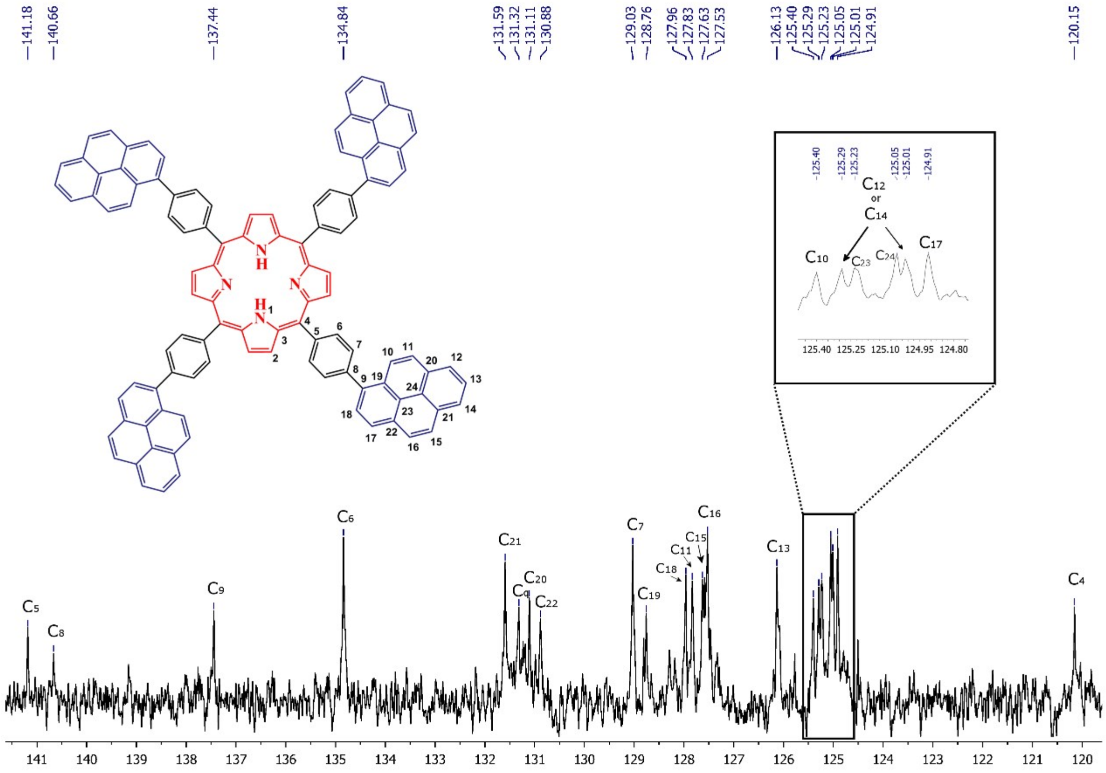

Figure S32: ^{13}C {^{1}H} NMR (126 MHz, $CDCl_3$) spectrum of **2H-PTetraPyr**.

Zn-PTetraCor

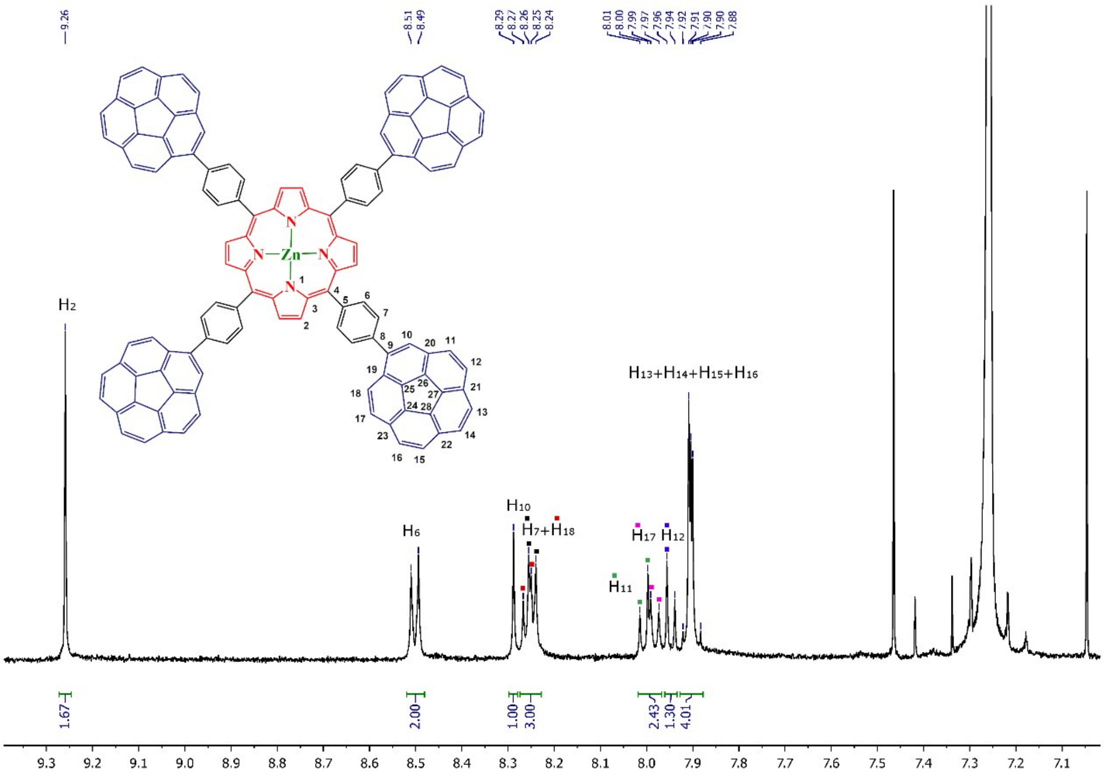

Figure S33: ^{1}H NMR (500 MHz, $CDCl_3$) spectrum of **Zn-PTetraCor**.

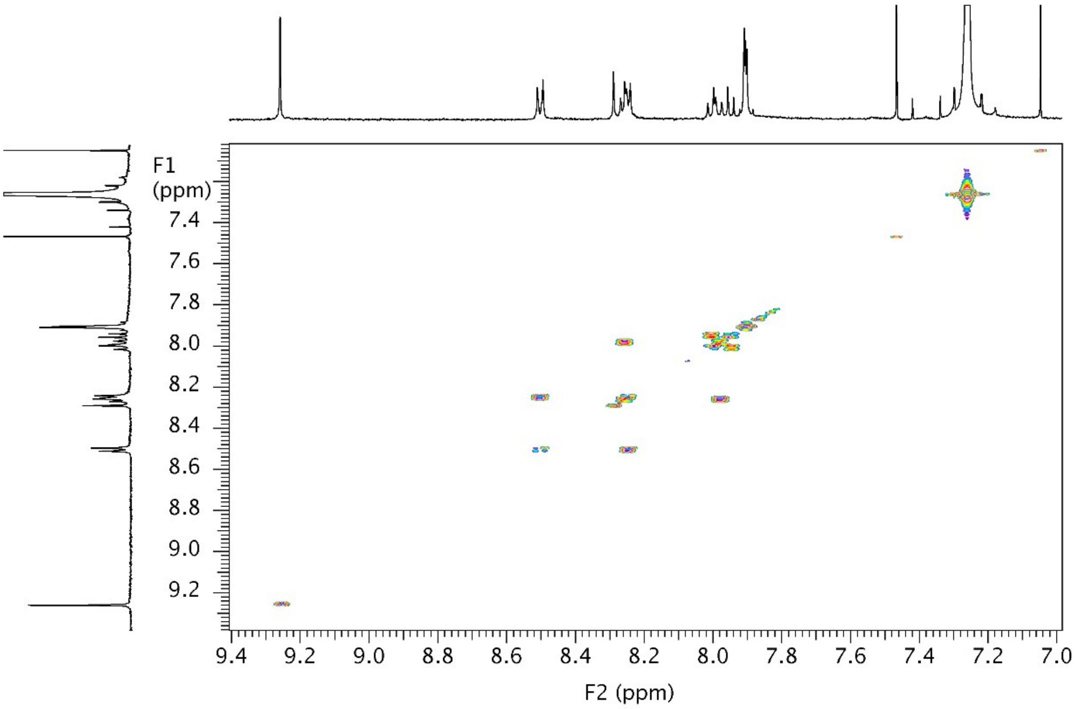

Figure S34: ^{1}H-^{1}H COSY ($CDCl_3$) of **Zn-PTetraCor**.

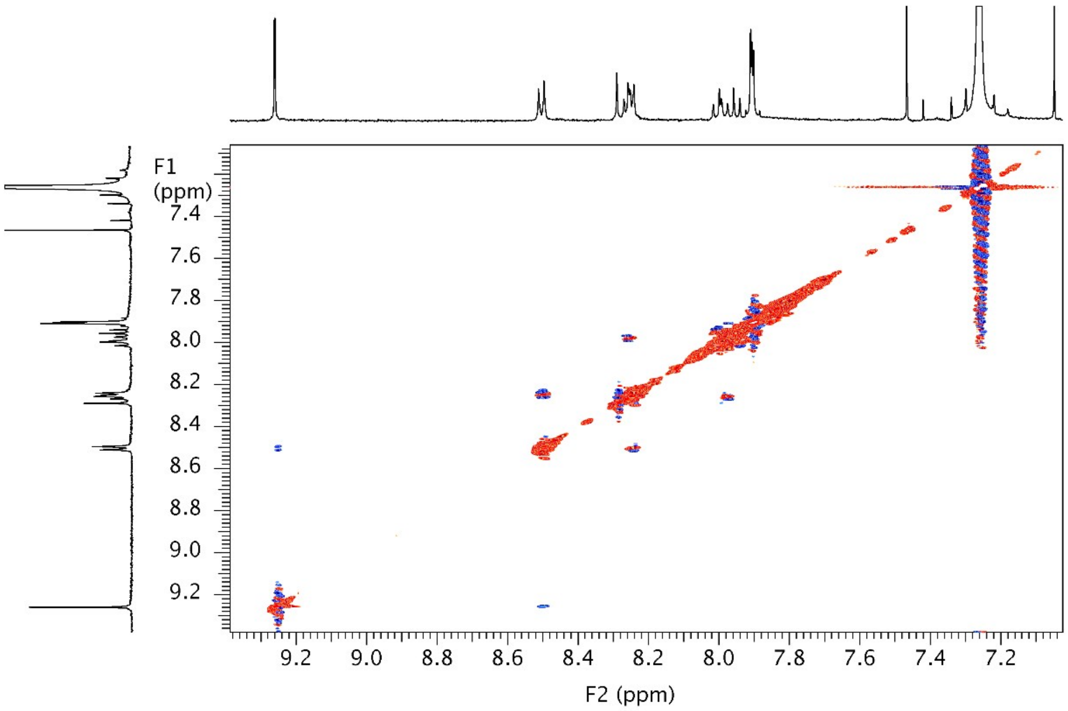

Figure S35: ^{1}H-^{1}H band selective ROESY ($CDCl_3$) of **Zn-PTetraCor**.

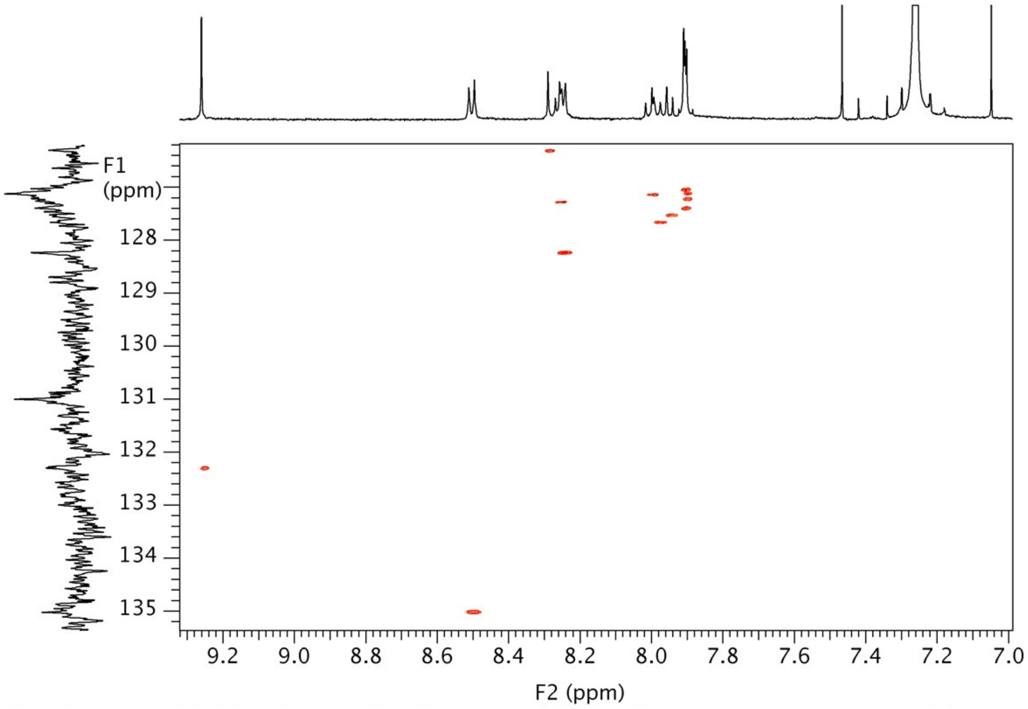

Figure S36: ^{1}H-^{13}C band selective HSQC ($CDCl_3$) of **Zn-PTetraCor**.

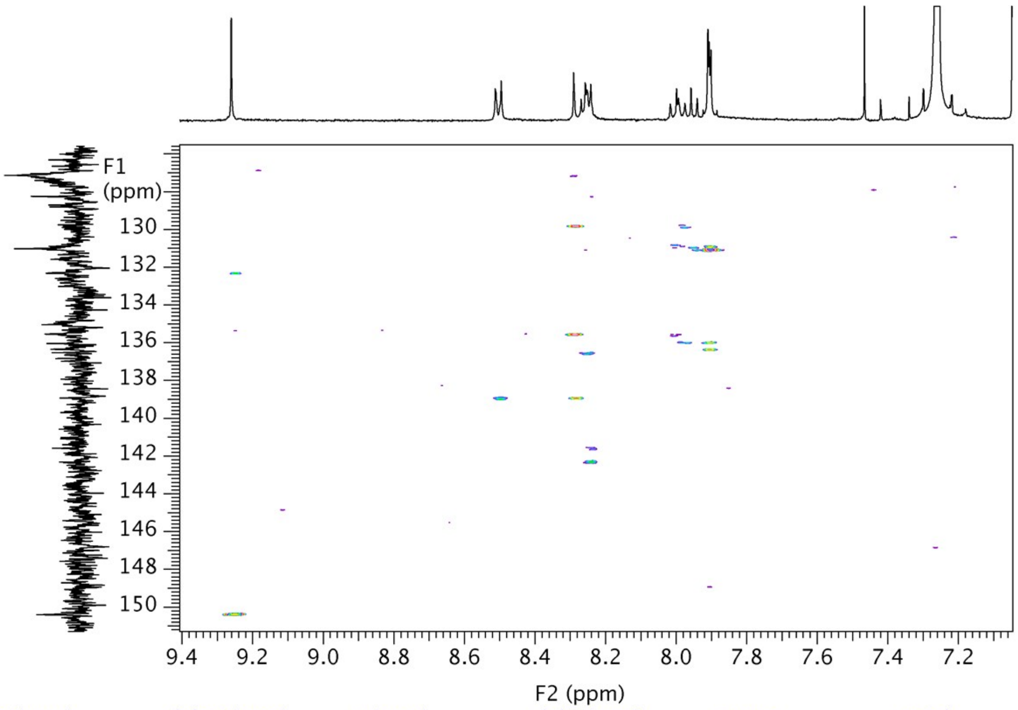

Figure S37: ^{1}H-^{13}C band selective HMBC ($CDCl_3$) of **Zn-PTetraCor**.

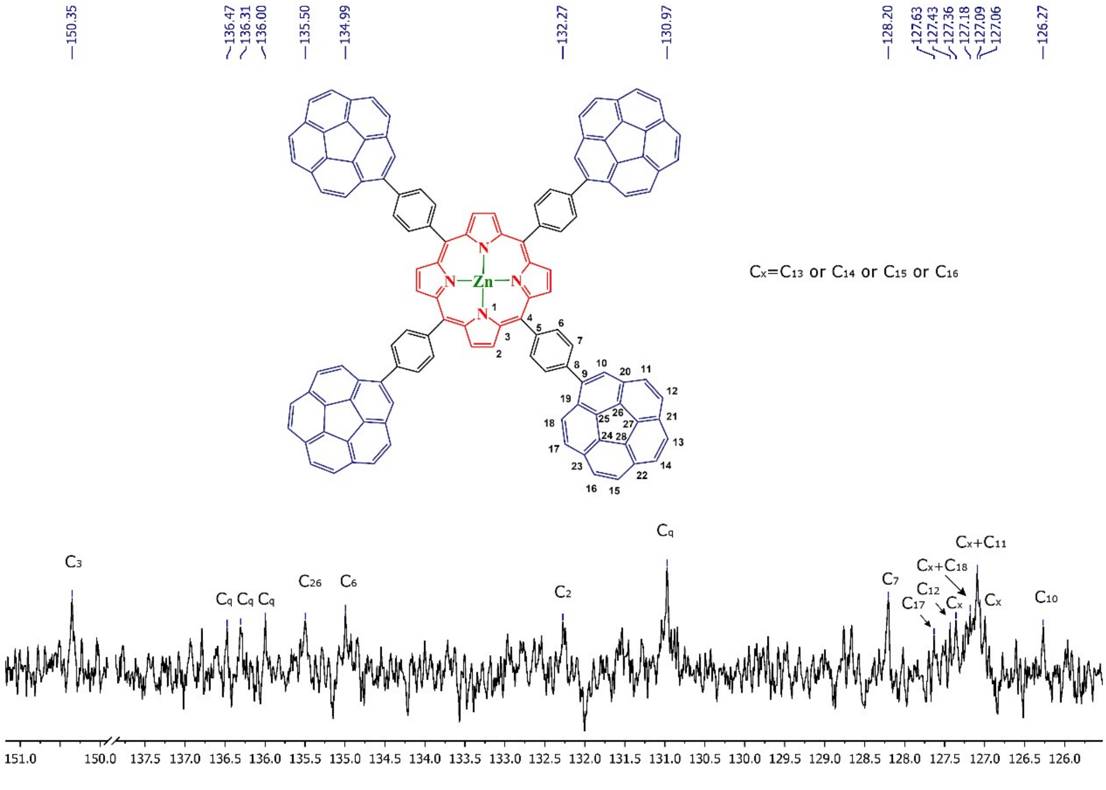

Figure S38: ^{13}C {^{1}H} NMR (126 MHz, $CDCl_3$) spectrum of **Zn-PTetraCor**.

2H-PTetraCor

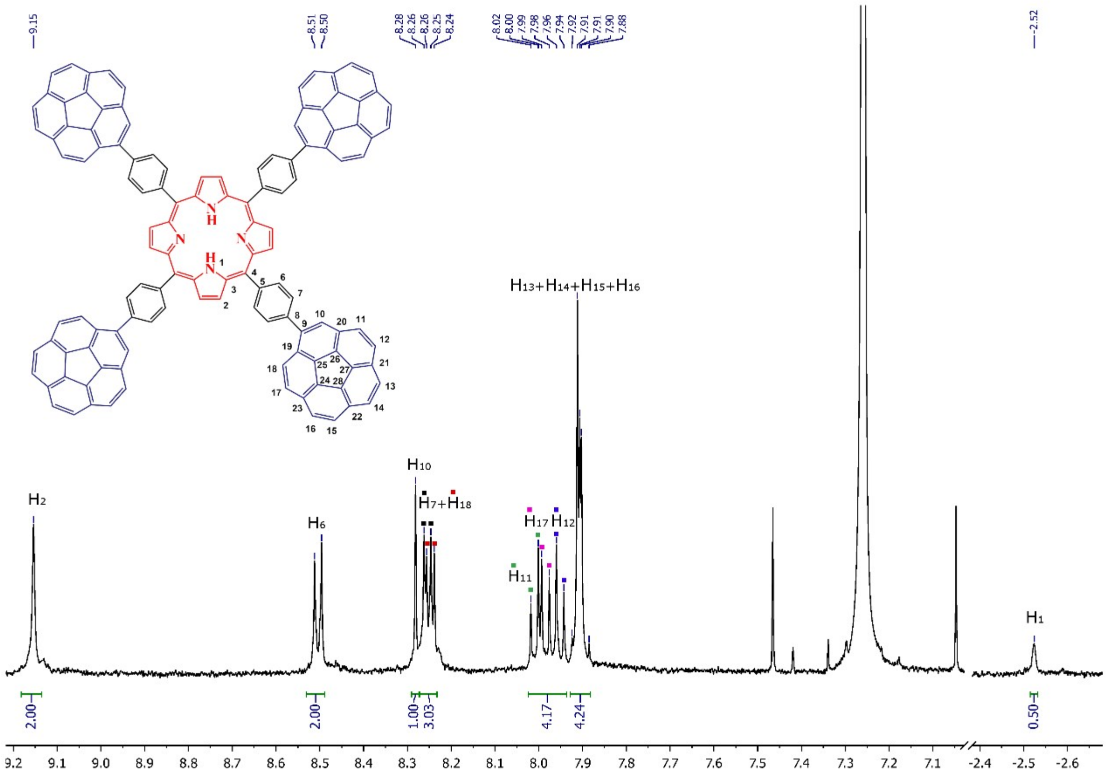

Figure S39: ^{1}H NMR (500 MHz, $CDCl_3$) spectrum of **2H-PTetraCor**.

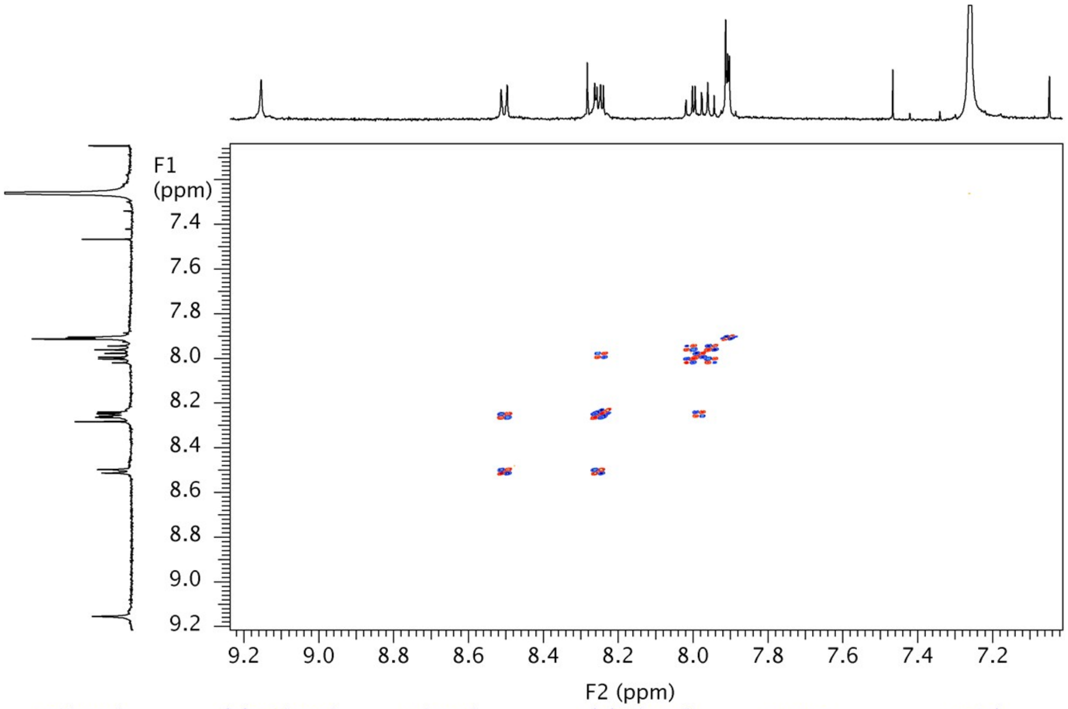

Figure S40: ^{1}H-^{1}H DQFCOSY ($CDCl_3$) of **2H-PTetraCor**.

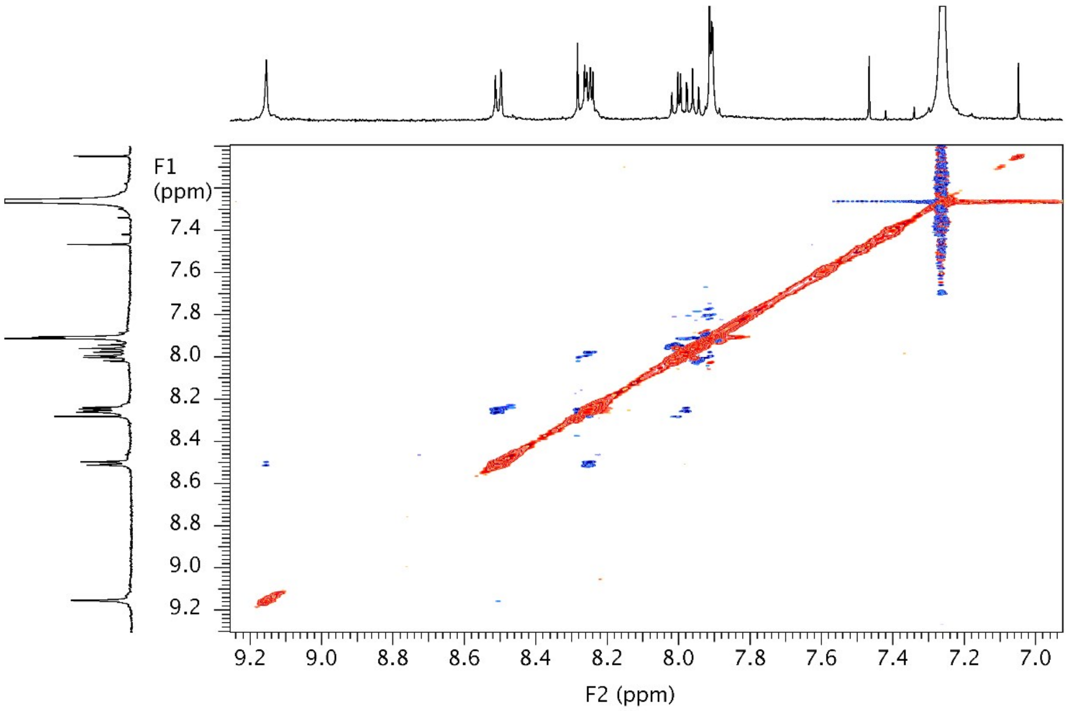

Figure S41: ^{1}H-^{1}H band selective ROESY ($CDCl_3$) of **2H-PTetraCor**.

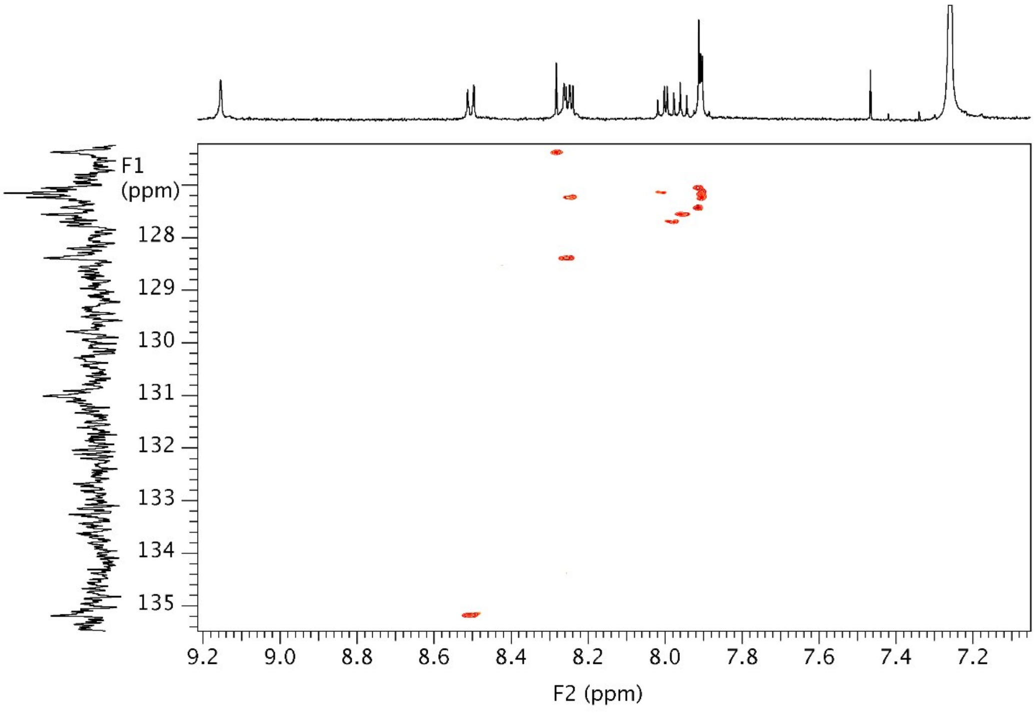

Figure S42: ^{1}H-^{13}C band selective HSQC ($CDCl_3$) of **2H-PTetraCor**.

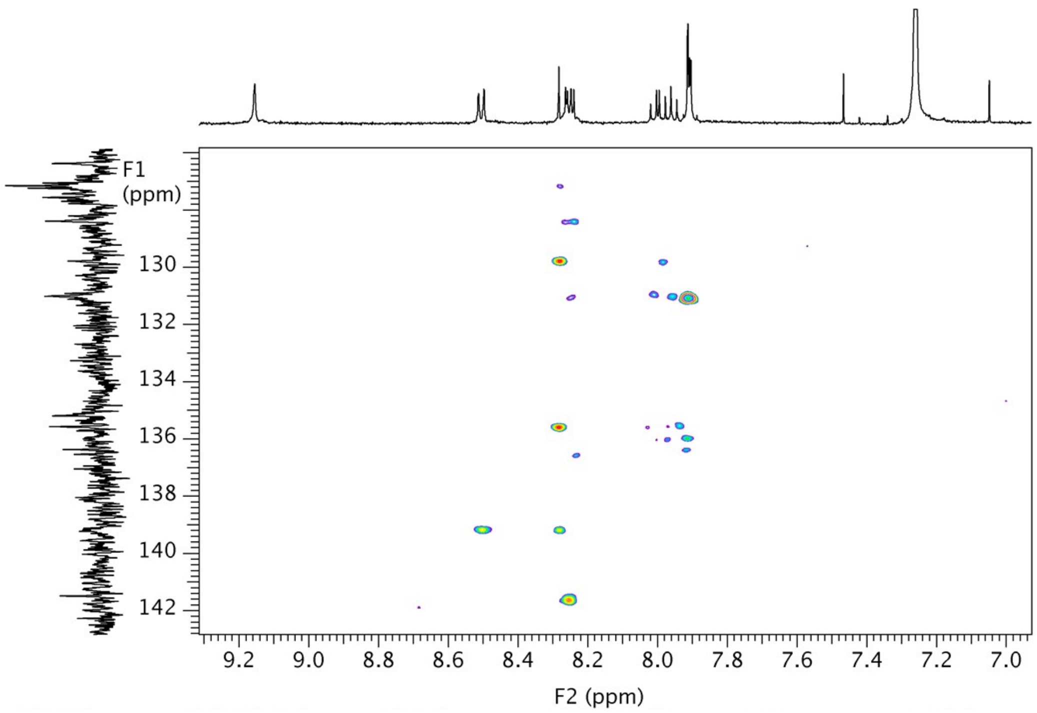

Figure S43: ^{1}H-^{13}C band selective HMBC ($CDCl_3$) of **2H-PTetraCor**.

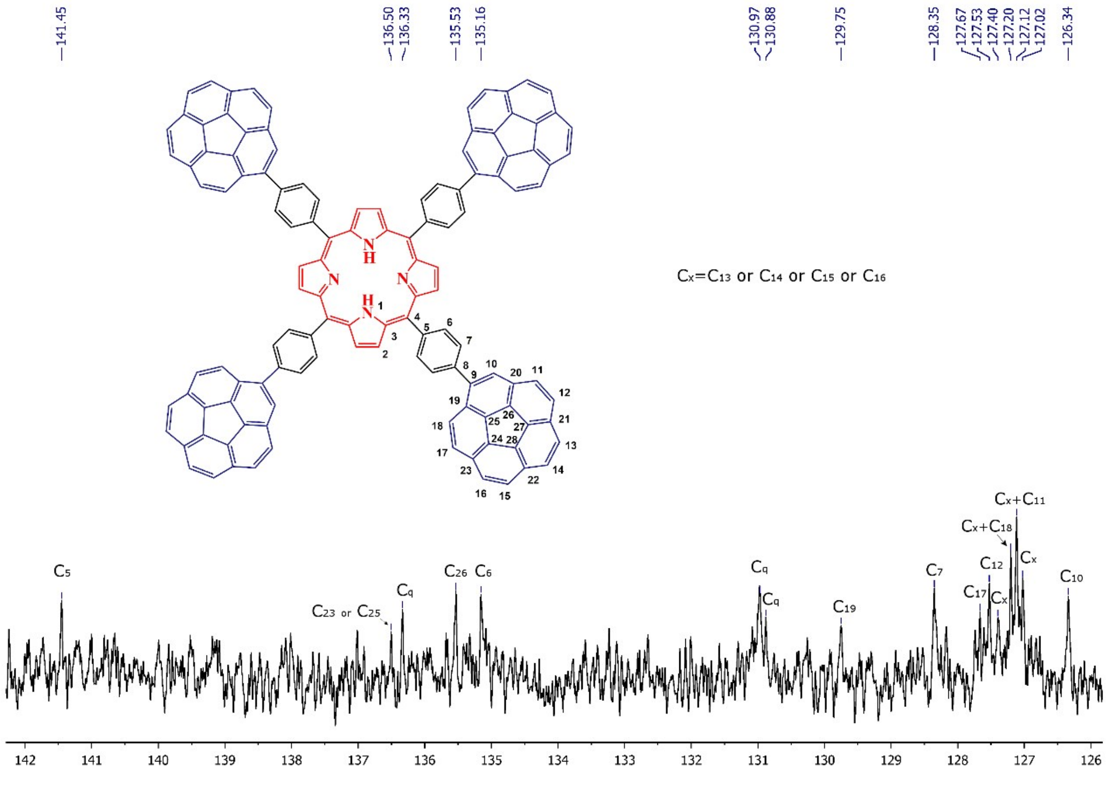

Figure S44: ^{13}C {^{1}H} NMR (126 MHz, $CDCl_3$) spectrum of **2H-PTetraCor**.

UV/Vis spectra

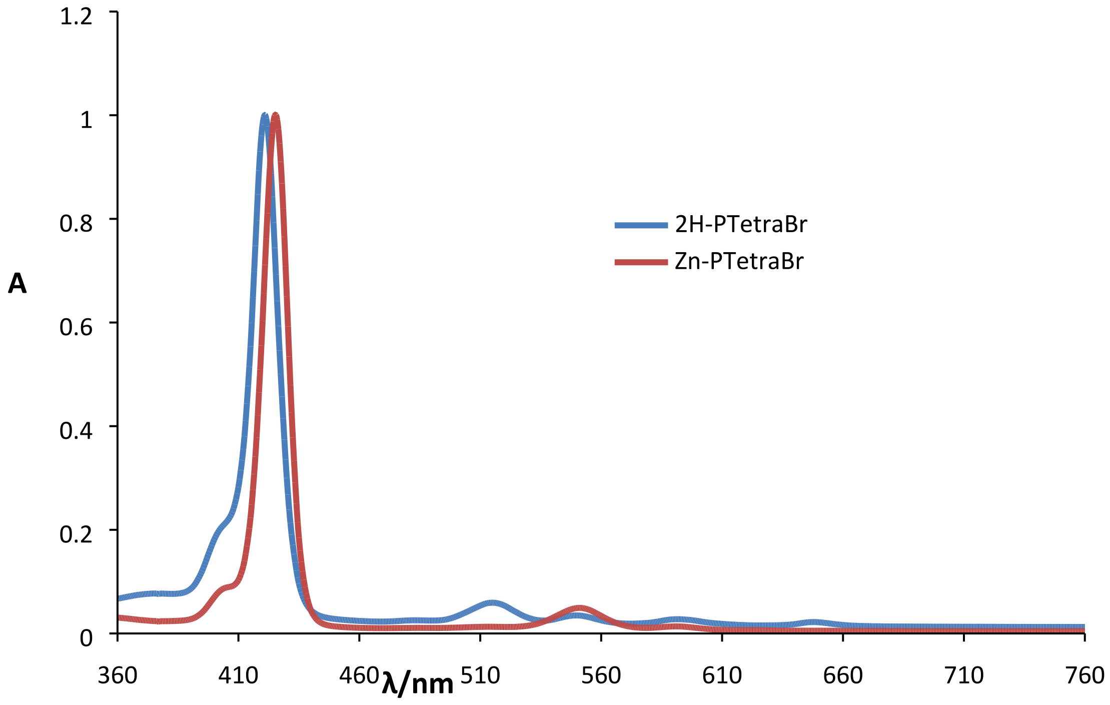

Figure S45: Normalized UV-Vis absorption spectra of **2H-PTetraBr** and **Zn-PTetraBr** in toluene.

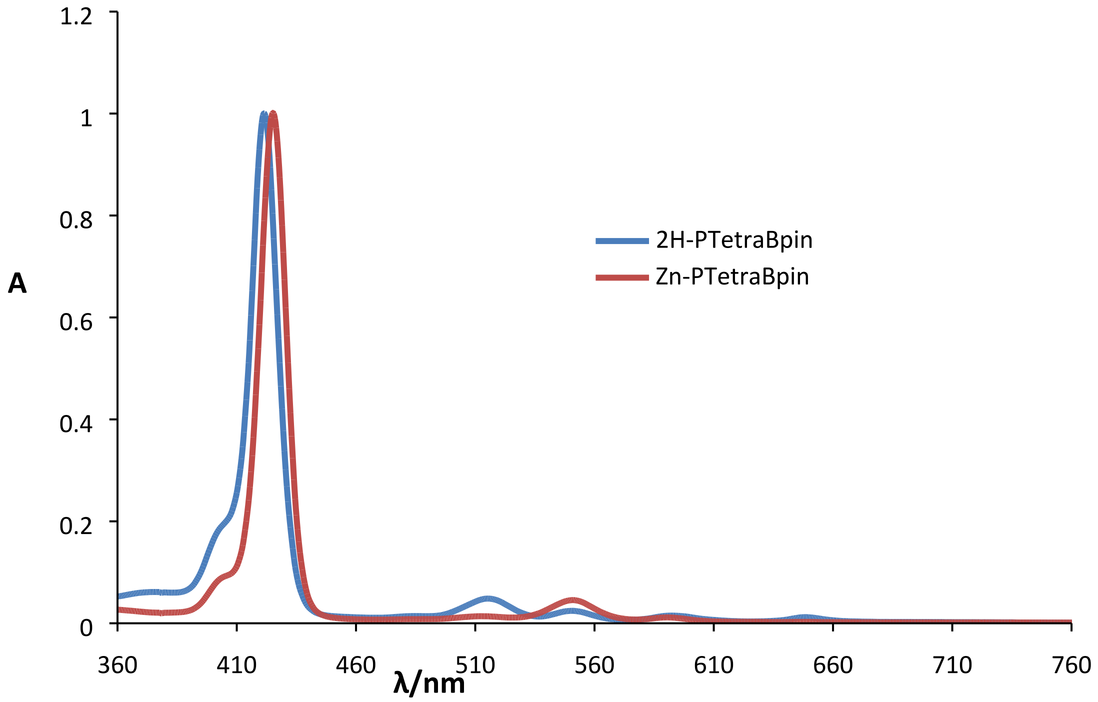

Figure S46: Normalized UV-Vis absorption spectra of **2H-PTetraBpin** and **Zn-PTetraBpin** in toluene.

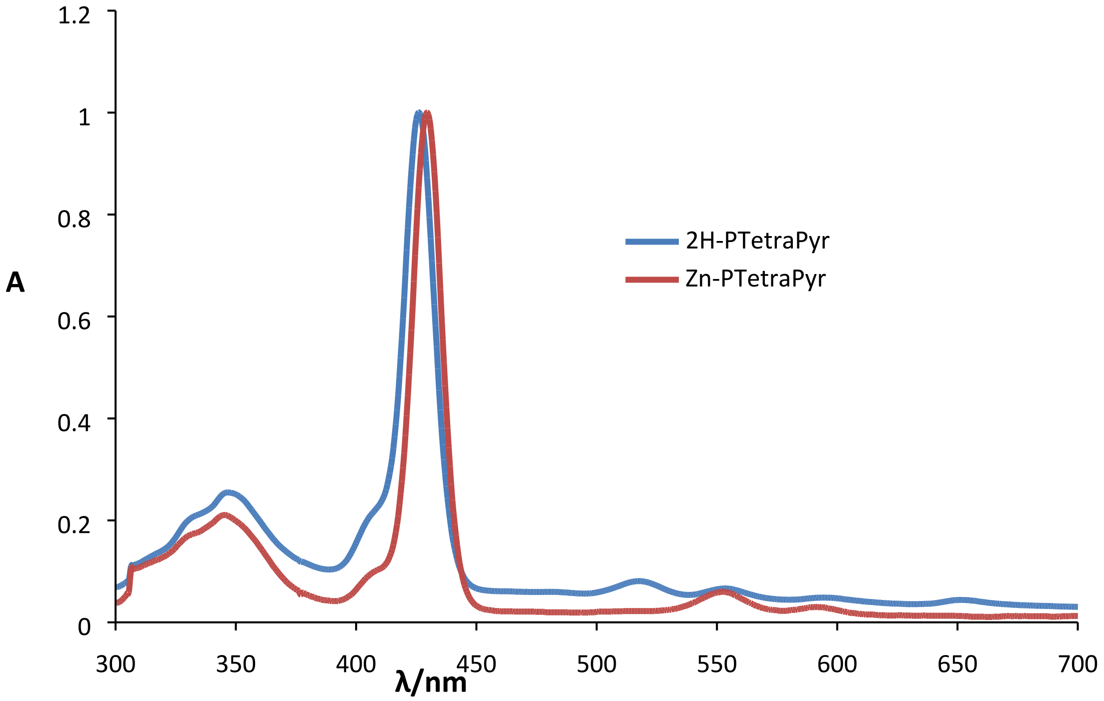

Figure S47: Normalized UV-Vis absorption spectra of **2H-PTetraPyr** and **Zn-PTetraPyr** in toluene.

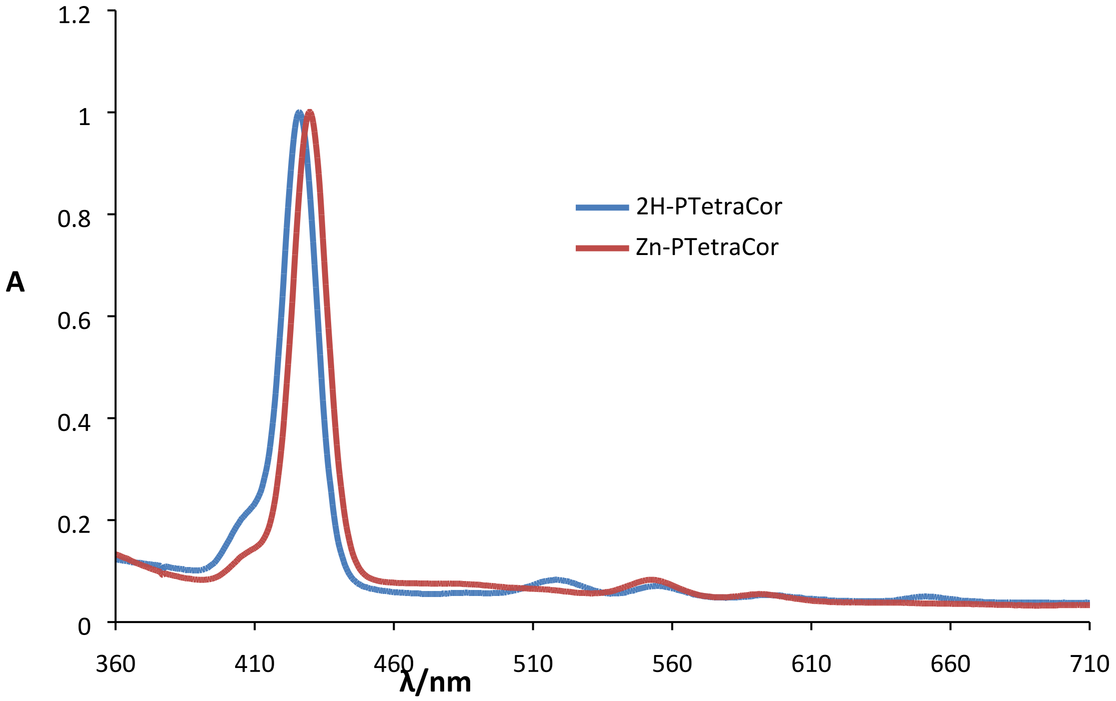

Figure S48: Normalized UV-Vis absorption spectra of **Zn-PTetraCor** and **2H-PTetraCor** in toluene.

MS spectra

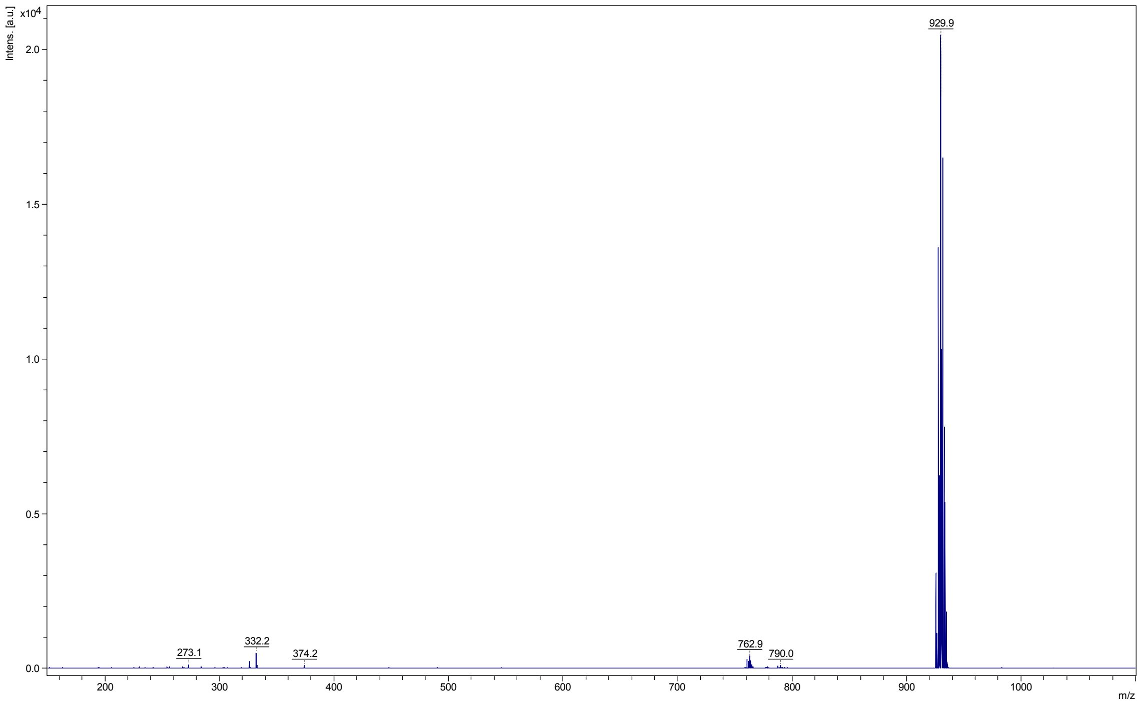

Figure S49: Full MS (MALDI-TOF) in DCTB of **2H-PTetraBr** $[M]^+$.

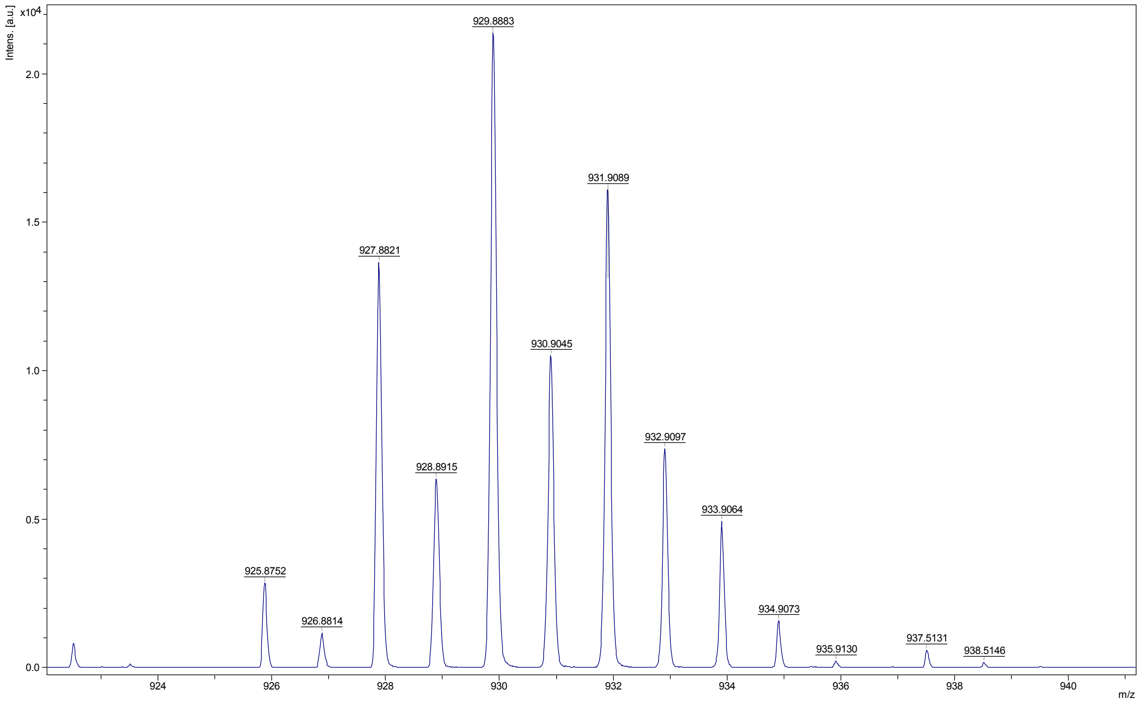

Figure S50: Expanded region of MS (MALDI-TOF) in DCTB of **2H-PTetraBr** $[M]^+$.

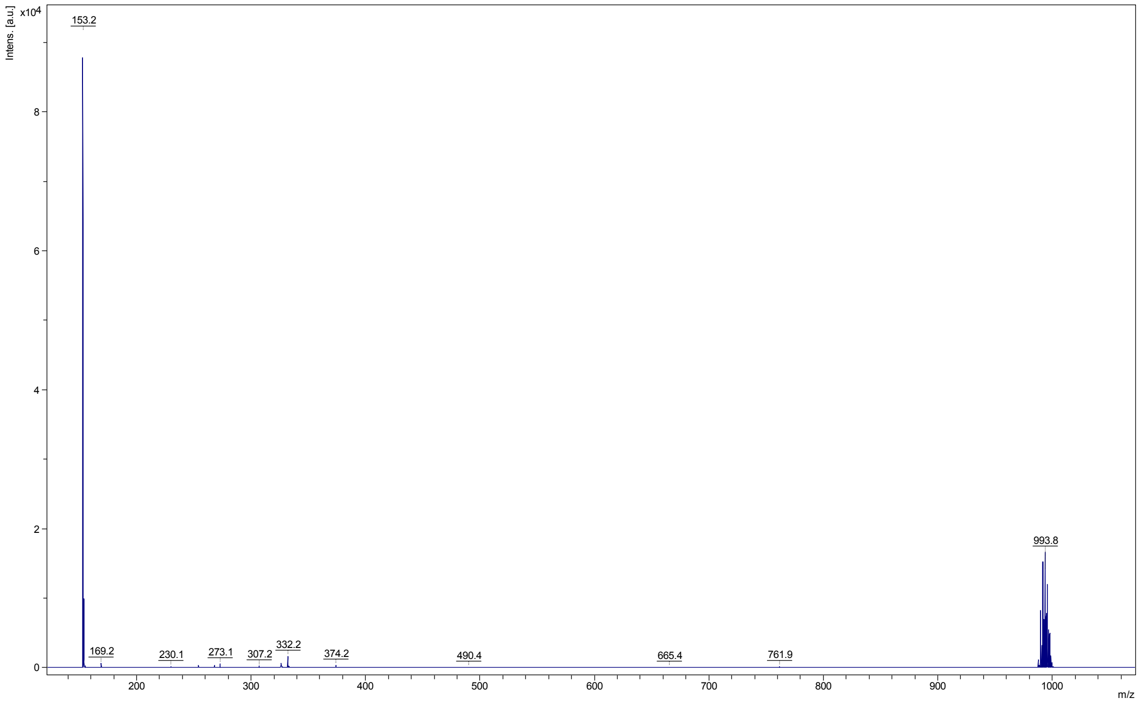

Figure S51: Full MS (MALDI-TOF) in DCTB of **Zn-PTetraBr** $[M]^+$.

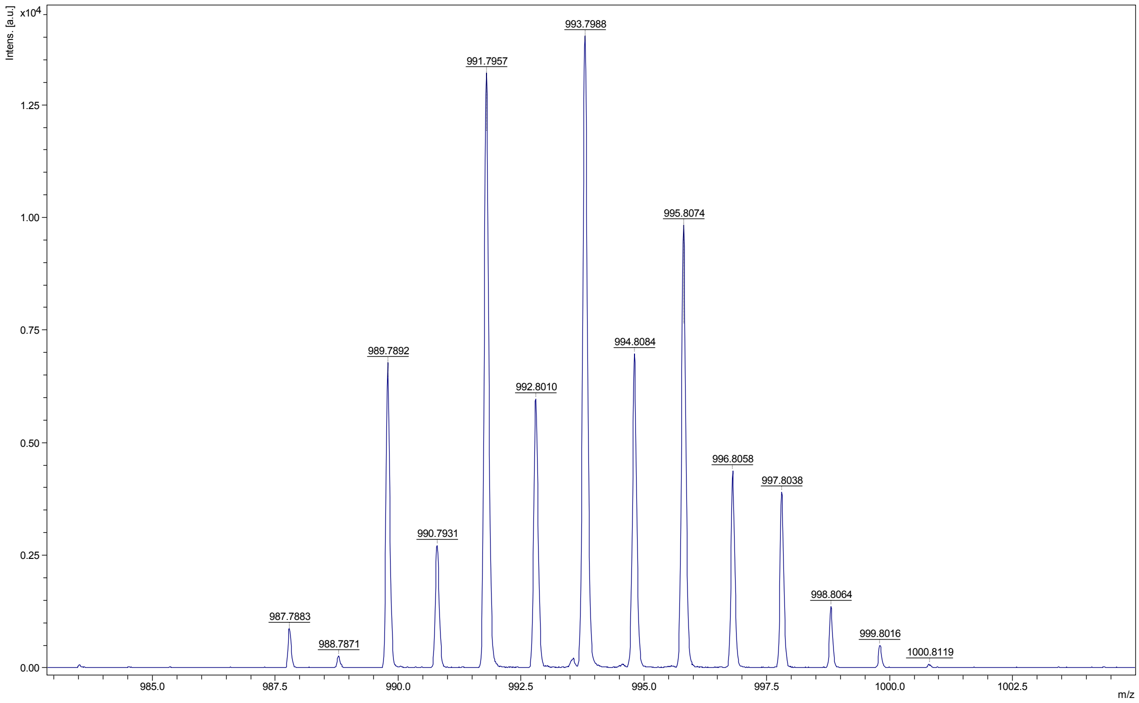

Figure S52: Expanded region of MS (MALDI-TOF) in DCTB of **Zn-PTetraBr** $[M]^+$.

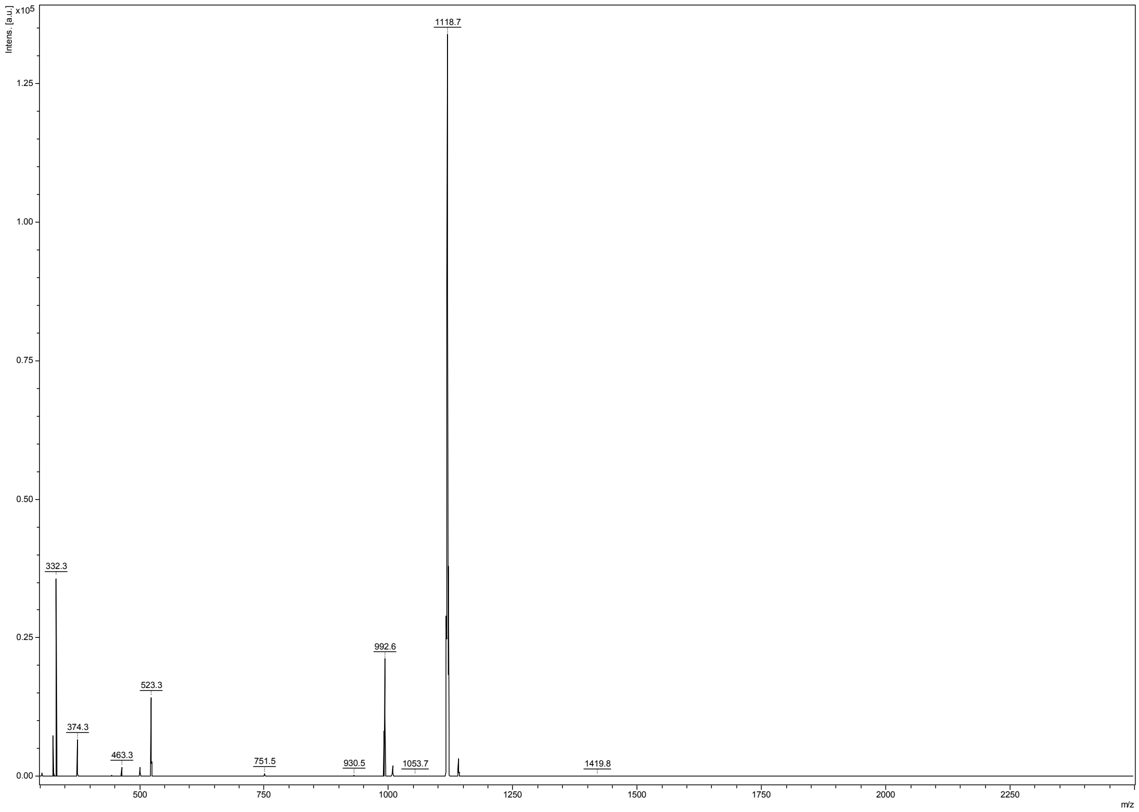

Figure S53: Full MS (MALDI-TOF) in DCTB of **2H-PTetraBpin** $[M]^+$.

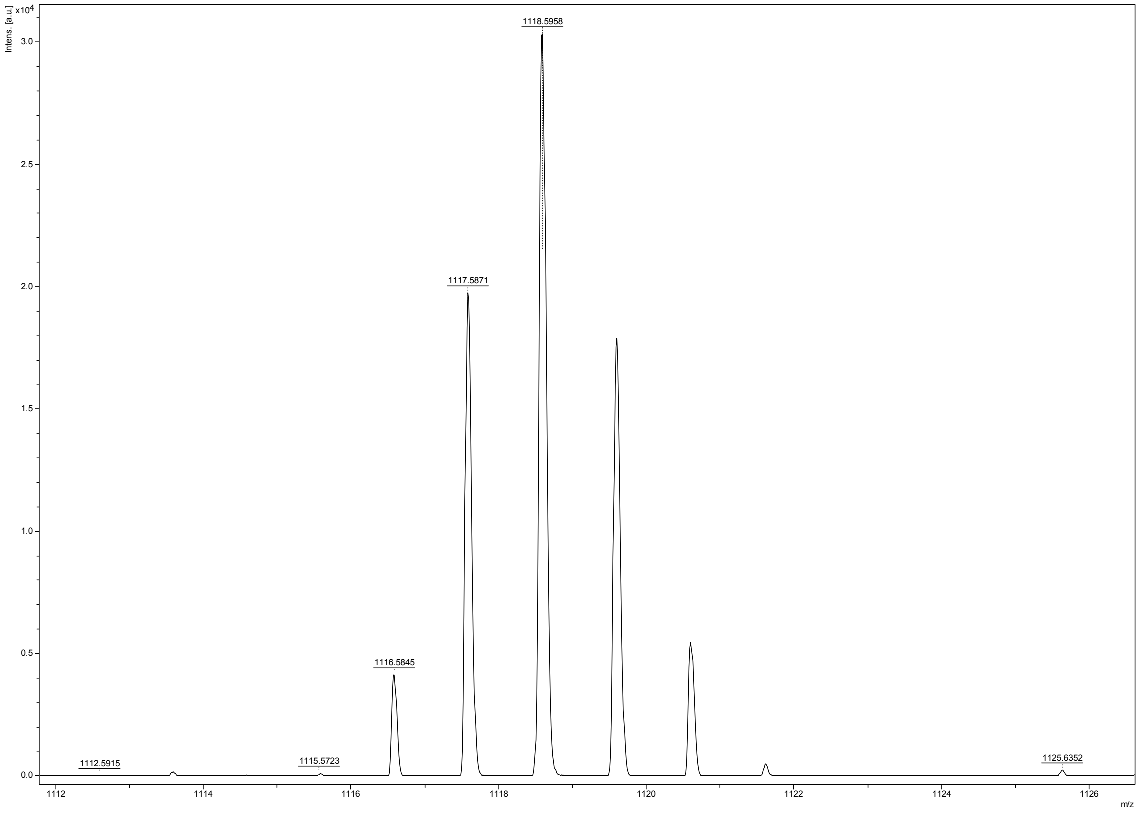

Figure S54: Expanded region of MS (MALDI-TOF) in DCTB of **2H-PTetraBpin** $[M]^+$.

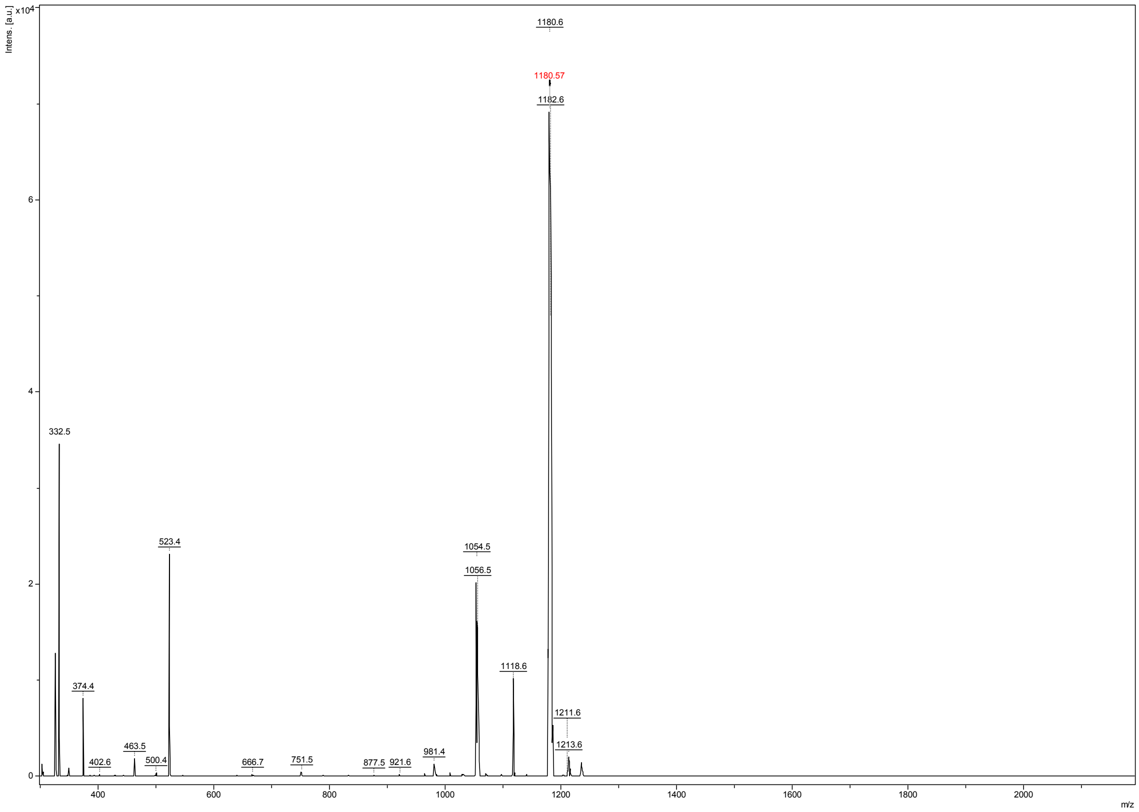

Figure S55: Full MS (MALDI-TOF) in DCTB of **Zn-PTetraBpin** [M]$^+$.

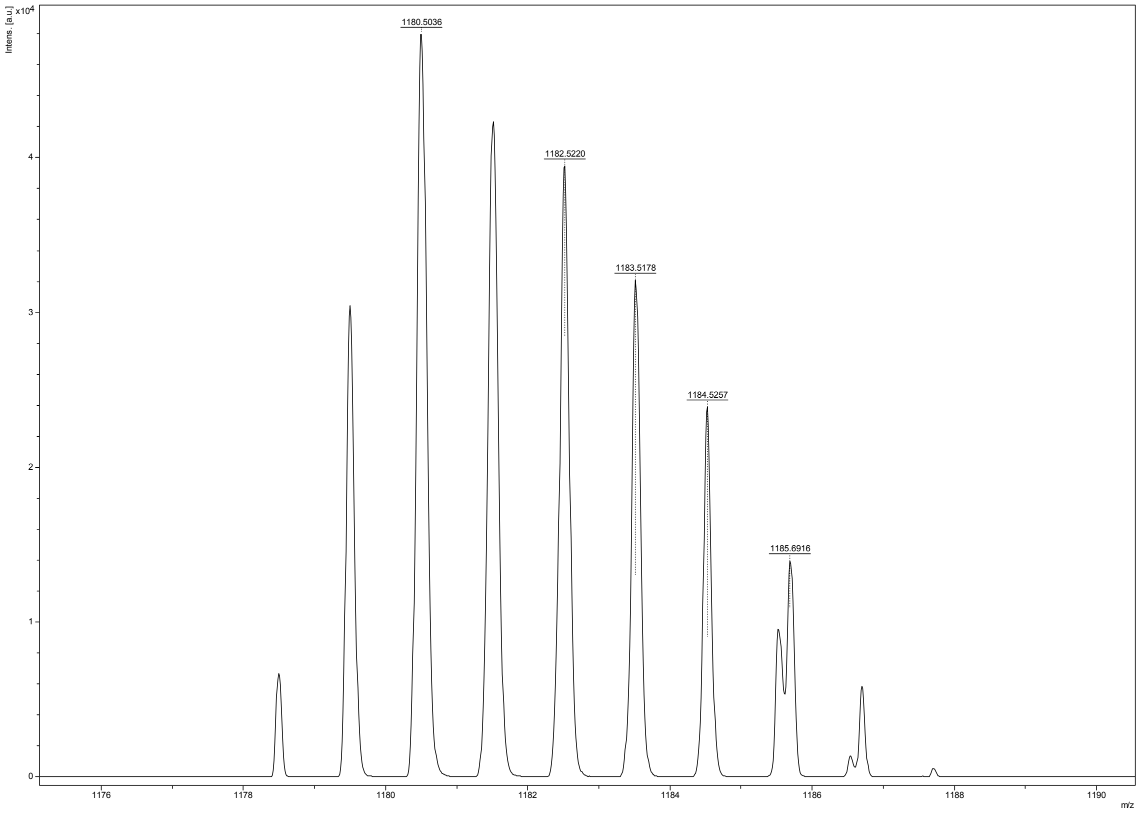

Figure S56: Expanded region of MS (MALDI-TOF) in DCTB of **Zn-PTetraBpin** [M]$^+$.

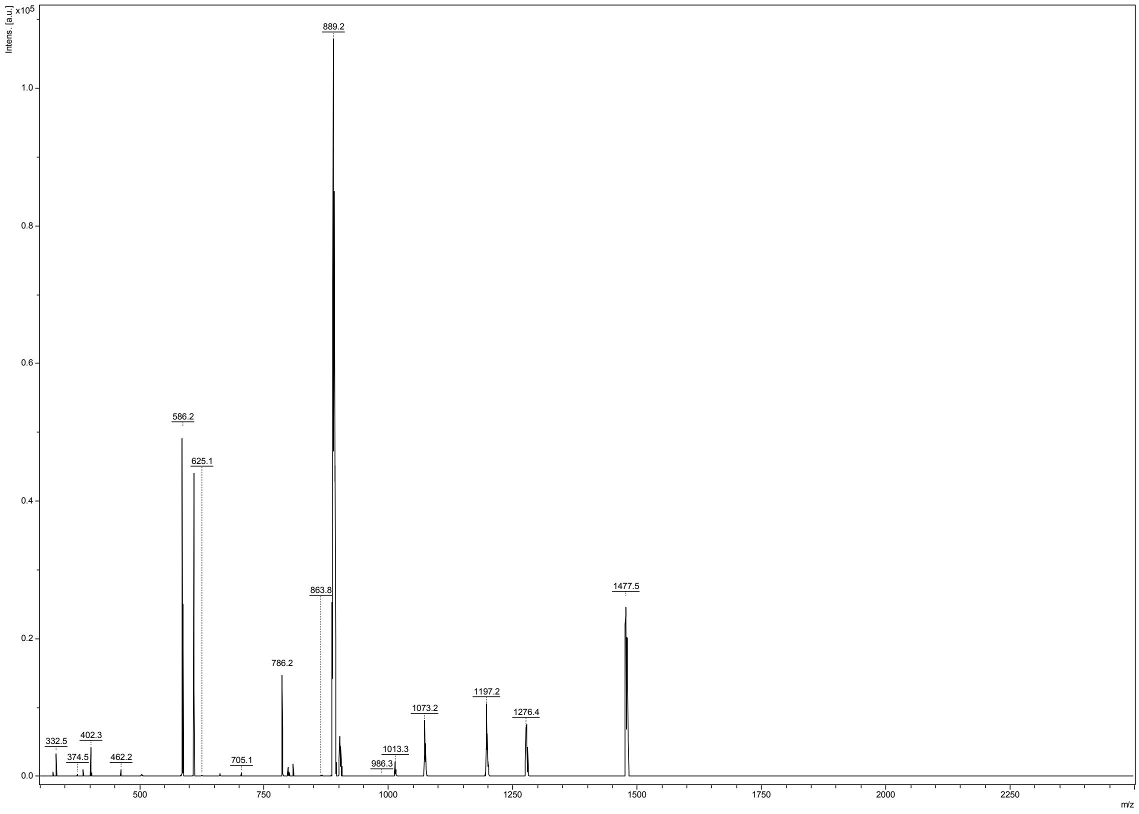

Figure S57: Full MS (MALDI-TOF) in DCTB of **Zn-PTetraPyr** $[M]^+$.

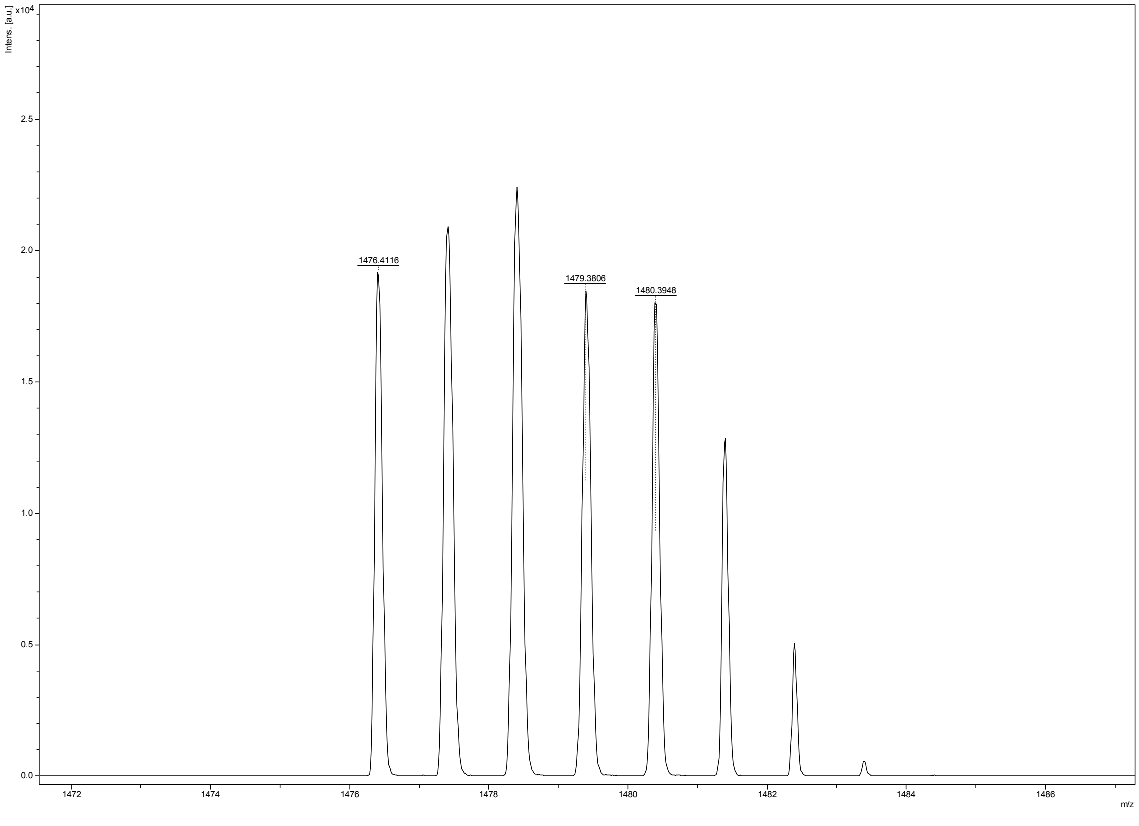

Figure S58: Expanded region of MS (MALDI-TOF) in DCTB of **Zn-PTetraPyr** $[M]^+$.

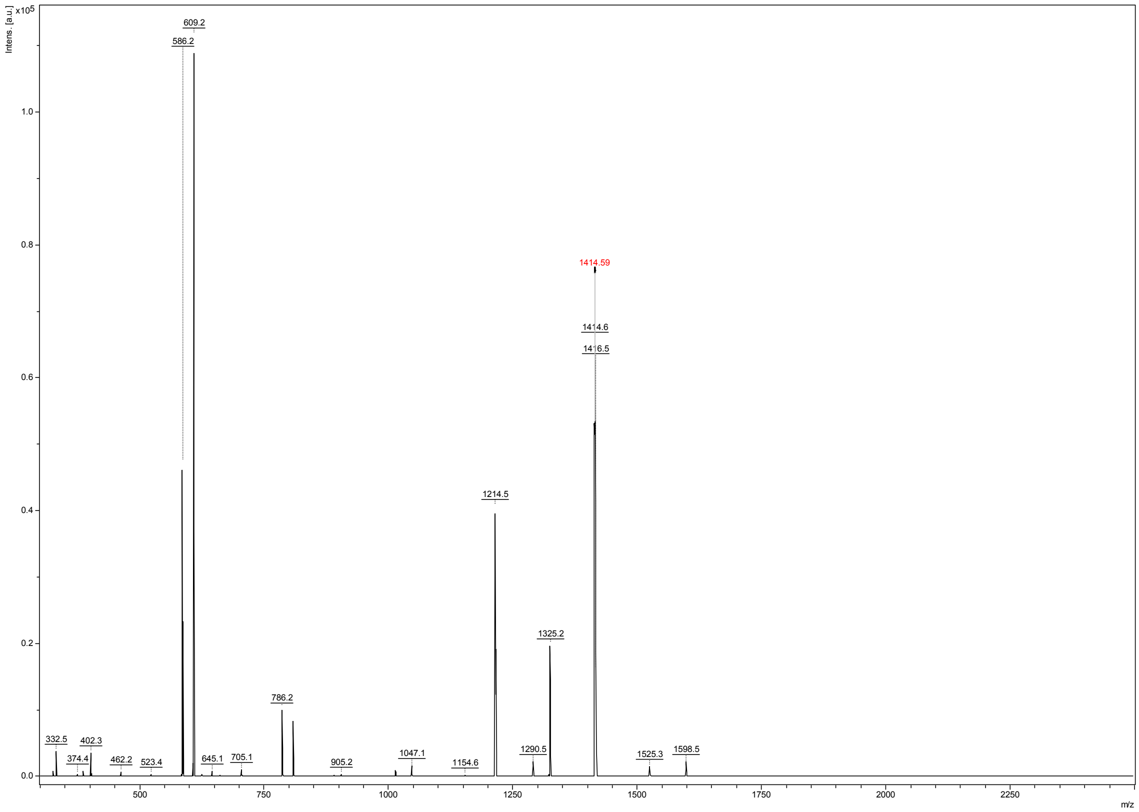

Figure S59: Full MS (MALDI-TOF) in DCTB of **2H-PTetraPyr** $[M]^+$.

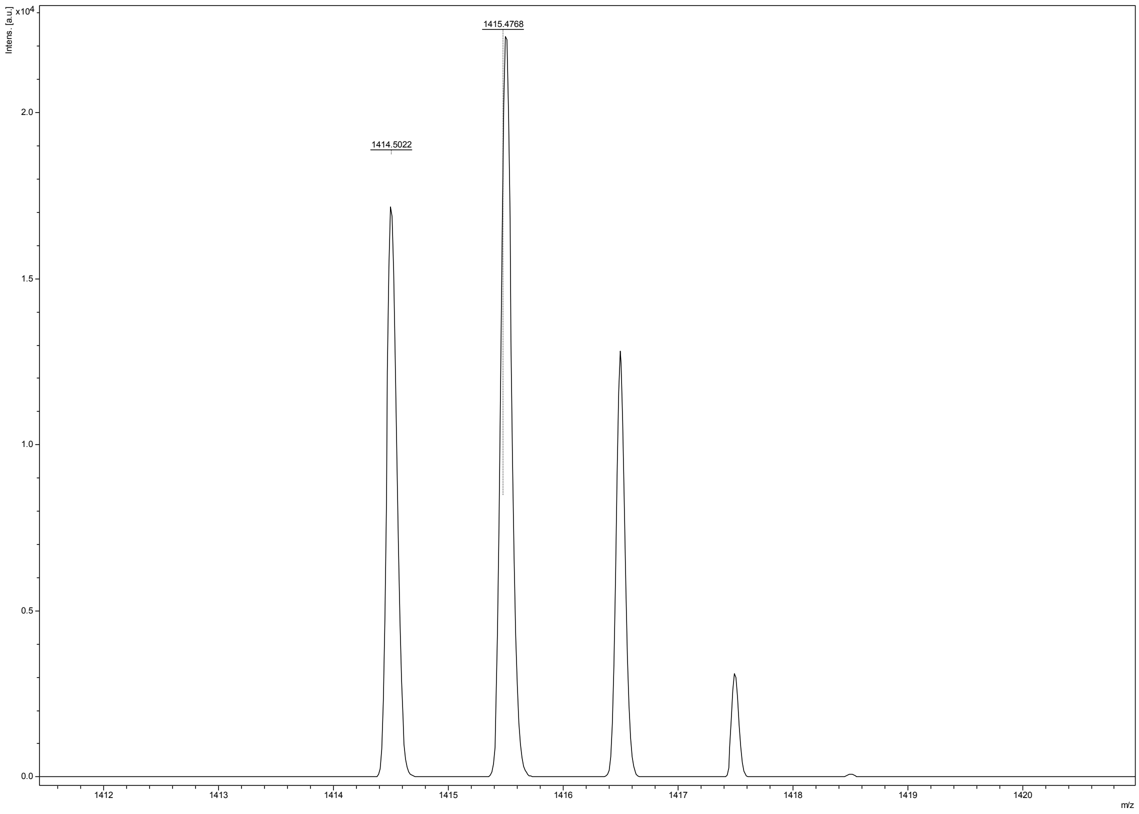

Figure S60: Expanded region of MS (MALDI-TOF) in DCTB of **2H-PTetraPyr** $[M]^+$.

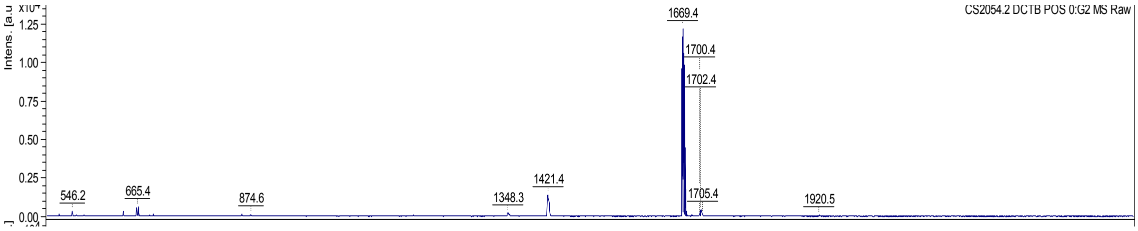

Figure S61: Full MS (M ALDI-TOF) in DCTB of **Zn-PTetraCor** [M]$^+$.

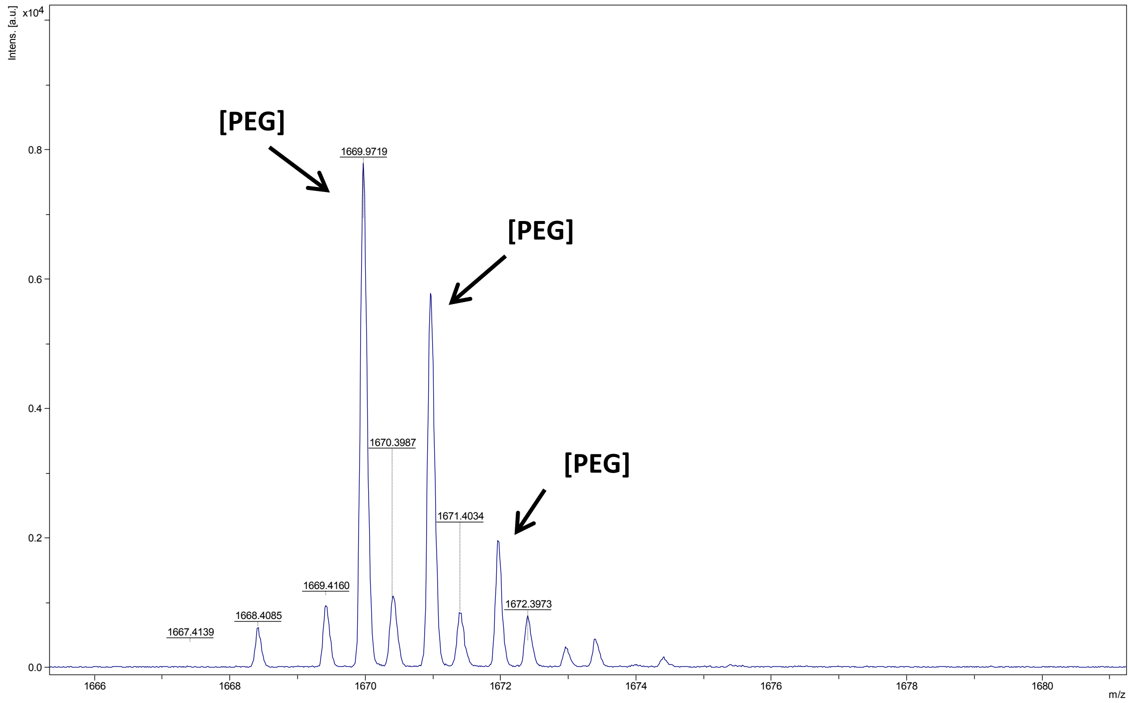

Figure S62: Expanded region of MS (MALDI-TOF) in DCTB of **Zn-PTetraCor** [M]$^+$.

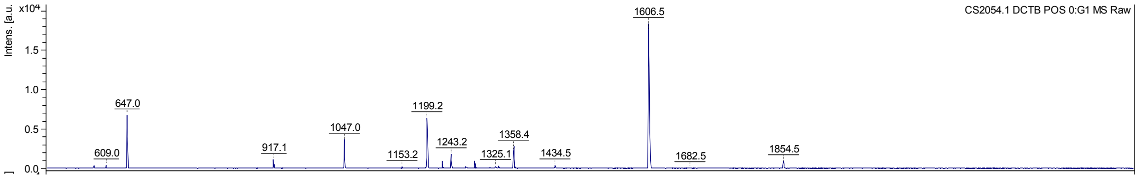

Figure S63: Full MS (MALDI-TOF) in: : 1-Dithranol-negative, 2-Dithranol-positive, 3-DCTB-positive, 4-DCTB-negative of **2H-PTetraCor** [M]$^+$.

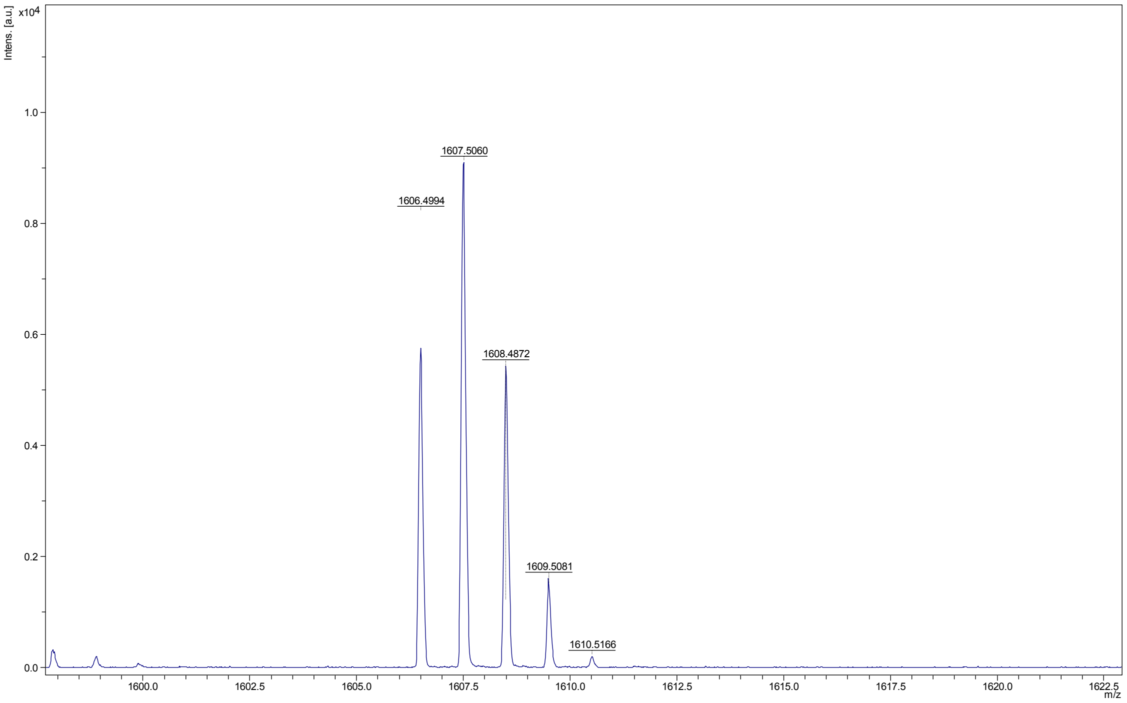

Figure S64: Expanded region of MS (MALDI-TOF) in DCTB of **2H-PTetraCor** $[M]^+$.

Complexation measurements

In order to estimate the association constants (K_a) of the compounds **Zn-PTetraCor** and **2H-PTetraCor** with fullerenes, the dilution method was applied. A 10^{-4} M deuterated toluene solution of each compound was prepared, and a known volume was transferred to an NMR tube (500 µL). The titration was carried out by adding known portions of a stock solution of C_{60} or C_{70} (10^{-3} M) in deuterated toluene to cover a wide range of equivalents. A ^1H NMR spectrum was recorded at room temperature after each addition. Once all data had been obtained, the changes in the chemical shifts (Δδ) of selected protons were plotted as a function of the molar fraction of the guest, and the resulting curve was fitted by a nonlinear method using the global analysis approach according to the following equations, depending on the type of equilibrium:[1]

<u>1:1 Equilibria</u>

General expression for the equilibrium constant:

$$K_a = \frac{[HG]}{[H][G]} \qquad \text{eq.1}$$

Changes upon NMR titration:

$$\Delta\delta = \Delta\delta_{\Delta HG}\left(\frac{[HG]}{[H]_0}\right) \qquad \text{eq.2}$$

Where:

[HG] is the concentration of the guest of the complex, and is calculated using the following equation:

$$[HG] = \frac{1}{2}\left([G_0] + [H_0] + \frac{1}{K_a}\right) - \sqrt{\left([G_0] + [H_0] + \frac{1}{K_a}\right)^2 + 4[G_0][H_0]}$$

eq.3

Where:

$[G_0]$ is the total concentration of the guest

$[H_0]$ is the total concentration of the host

$\Delta\delta_{\Delta HG}$ is Δδ at maximum complexation (100% supramolecular complex formation)

K_a is the estimated association constant for 1:1 equilibrium

$\Delta\delta_{\Delta HG}$ and K_a for a 1:1 equilibrium were extracted using the non-linear curve fitting tool at the open access web portal *http://supramolecular.org* (accessed since 2016). Links to all the fittings of the data are provided below for every case.

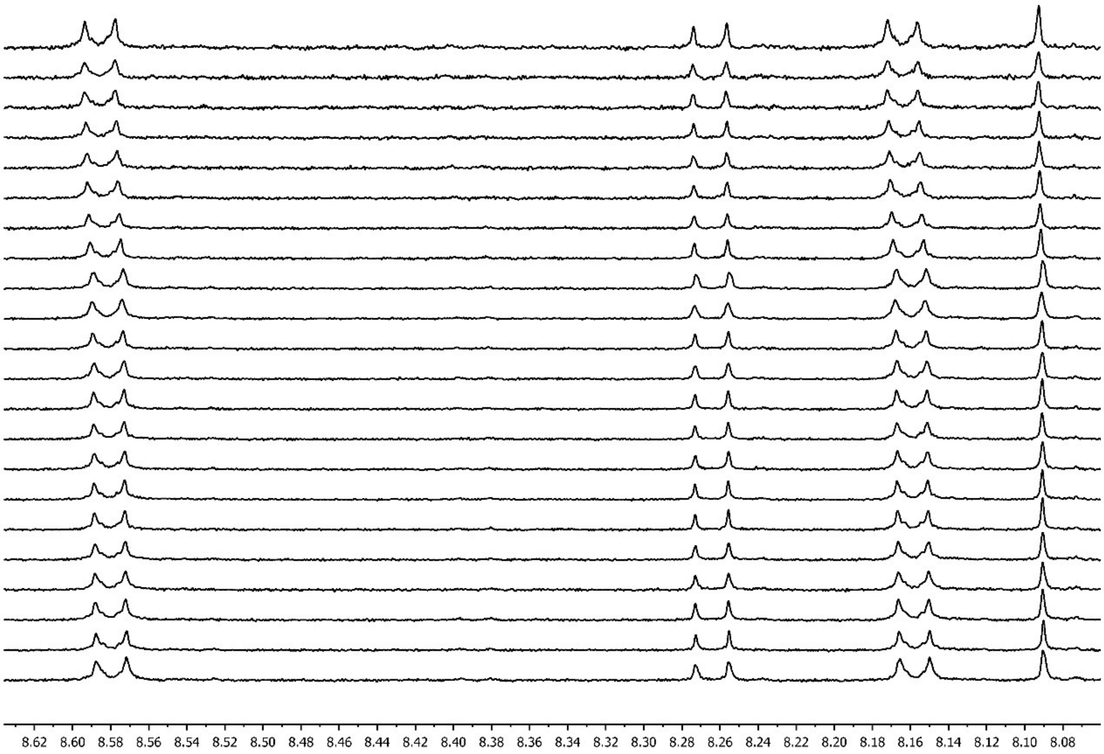

Figure S65: ^{1}H-NMR spectra of the titration of **Zn-PTetraCor** (10^{-4} M) with variable concentrations of C_{60} (10^{-3} M) in toluene-d_8.

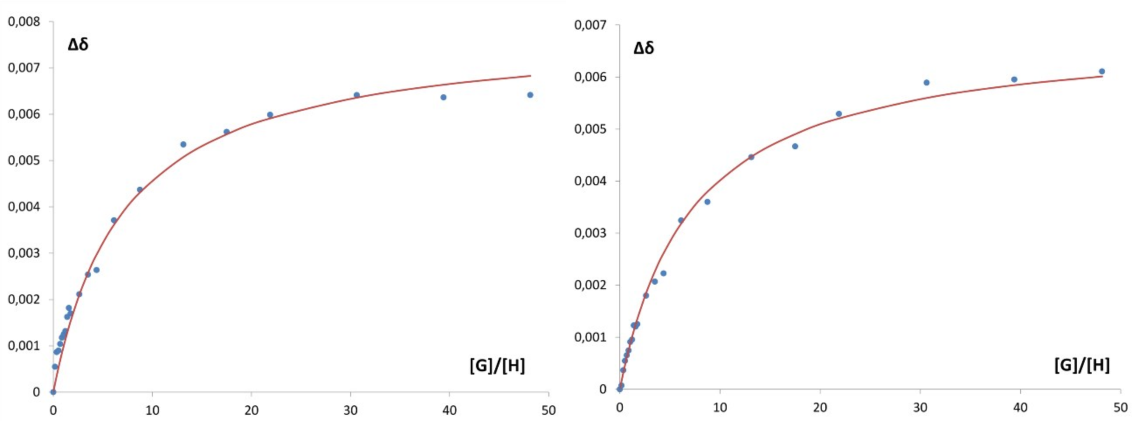

Figure S66: Nonlinear regressions for the results of the titration of **Zn-PTetraCor** (10^{-4} M) with C_{60} (10^{-3} M) for selected protons (right plot: H_6 proton, left plot: H_7, 1:1 binding model).

For additional information see:

http://app.supramolecular.org/bindfit/view/c40ba3c1-1658-43b7-965e-17b2e510871b

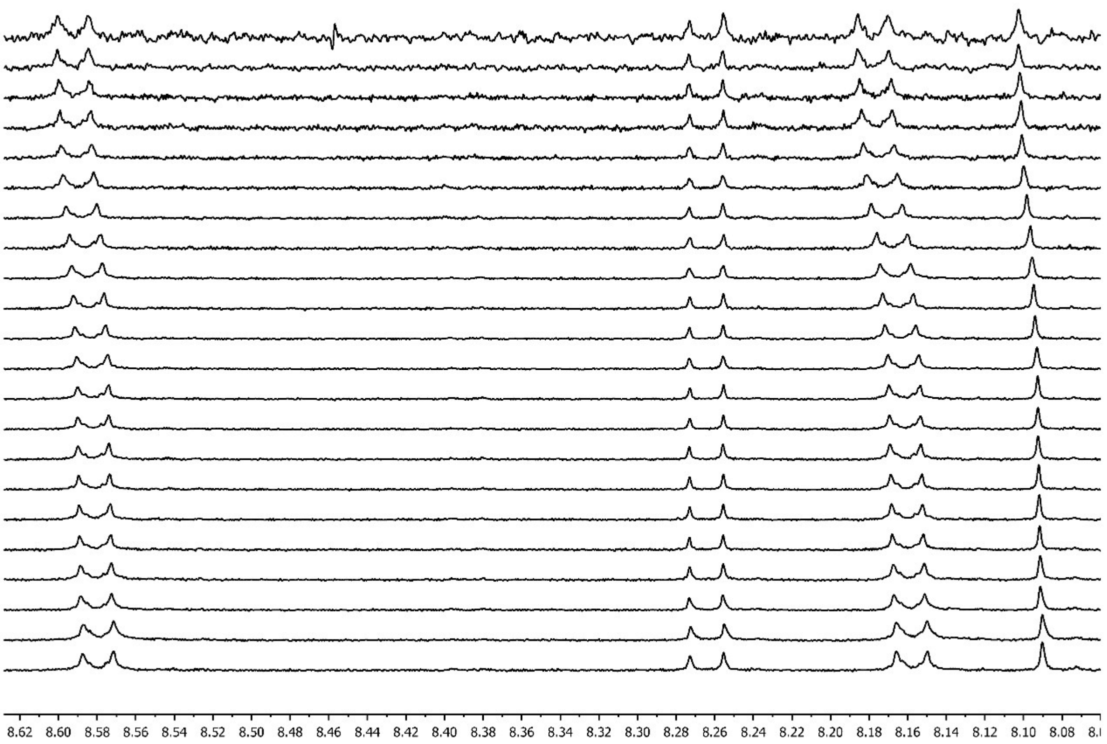

Figure S67: ^{1}H-NMR spectra of the titration of **Zn-PTetraCor** (10^{-4} M) with variable concentrations of C_{70} (10^{-3} M) in toluene-d_8.

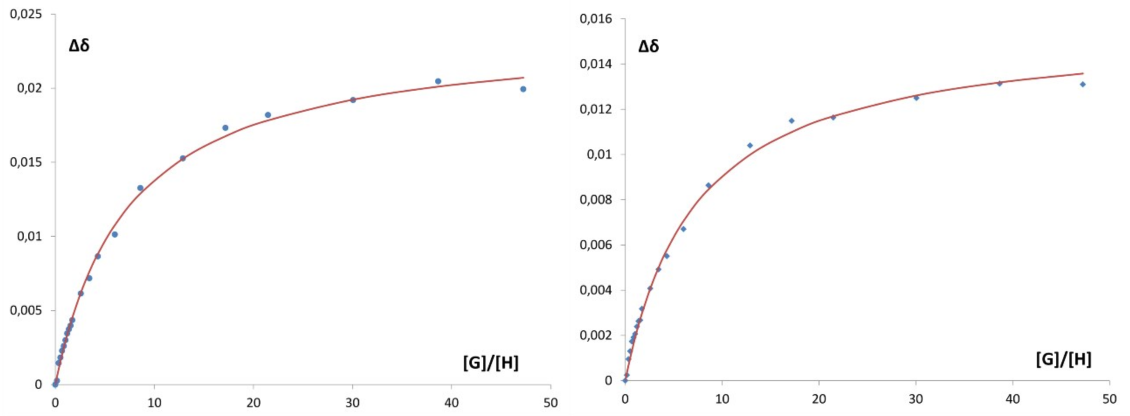

Figure S68: Nonlinear regressions for the results of the titration of **Zn-PTetraCor** (10^{-4} M) with C_{70} (10^{-3} M) for selected protons (right plot: H_6 proton, left plot: H_7, 1:1 binding model).

For additional information see:

http://app.supramolecular.org/bindfit/view/1d6856af-192c-4e5a-9d53-f609627ac179

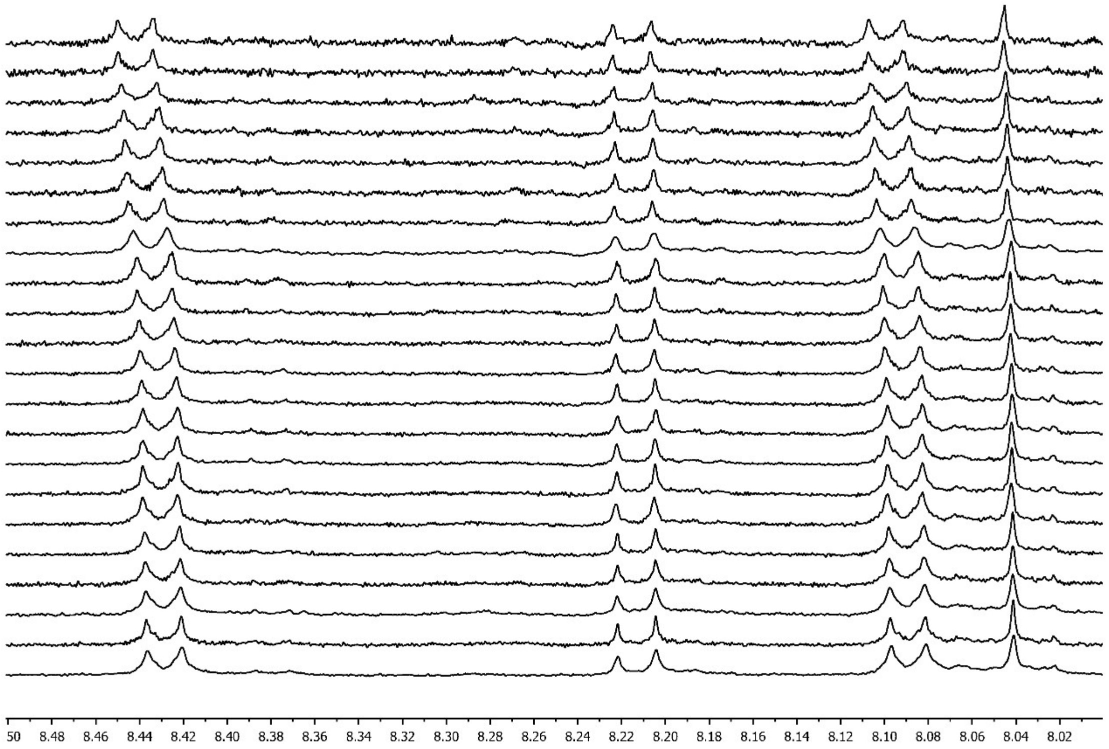

Figure S69: ^{1}H-NMR spectra of the titration of **2H-PTetraCor** (10^{-4} M) with variable concentrations of C_{60} (10^{-3} M) in toluene-d_8.

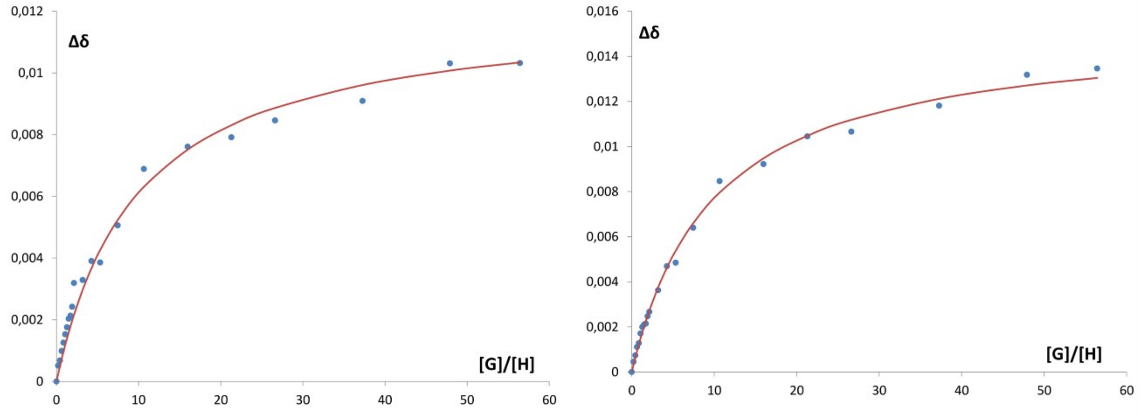

Figure S70: Nonlinear regressions for the results of the titration of **2H-PTetraCor** (10^{-4} M) with C_{60} (10^{-3} M) for selected protons (right plot: H_6 proton, left plot: H_7, 1:1 binding model).

For additional information see:

http://app.supramolecular.org/bindfit/view/41c6686e-e06a-41fd-bc7c-ee6955080966

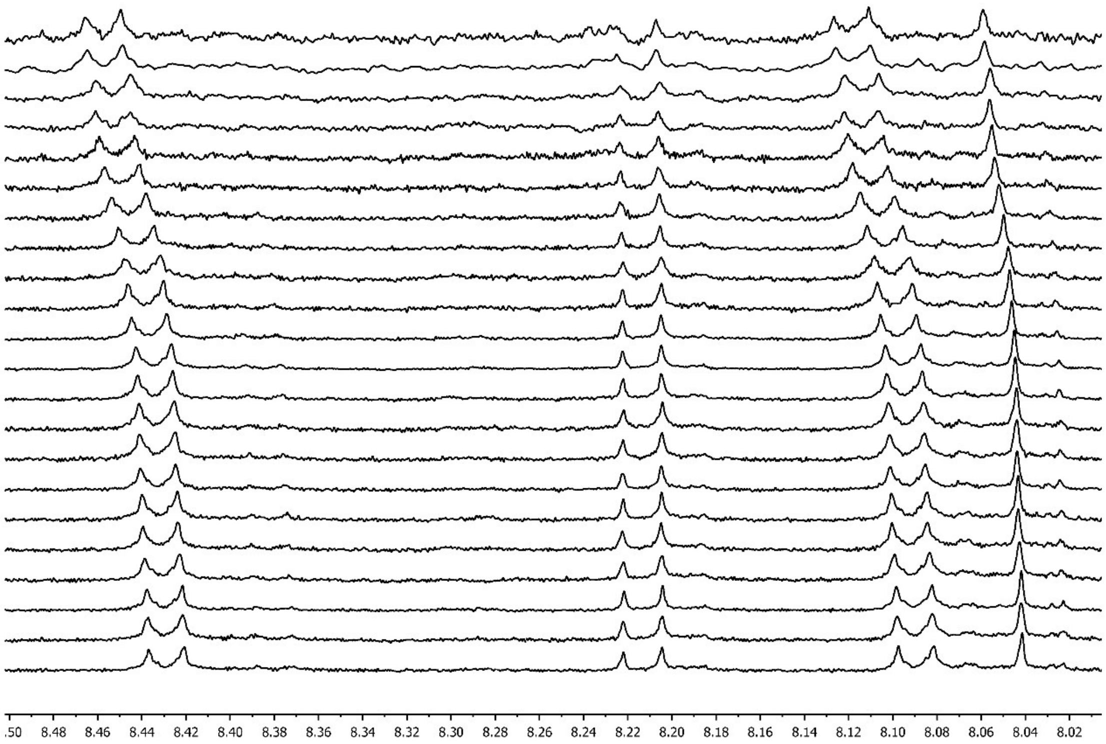

Figure S71: ^{1}H-NMR spectra of the titration of **2H-PTetraCor** (10^{-4} M) with variable concentrations of C_{70} (10^{-3} M) in toluene-d_8.

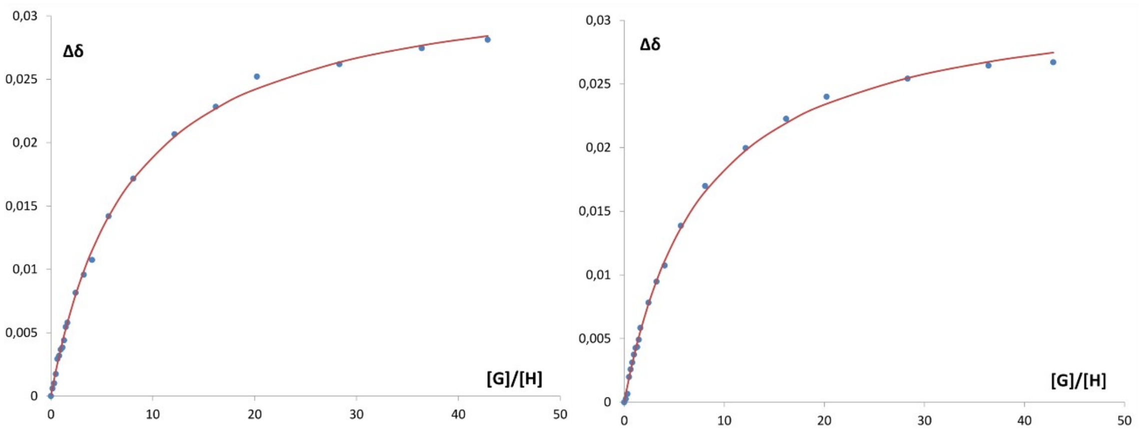

Figure S72: Nonlinear regressions for the results of the titration of **2H-PTetraCor** (10^{-4} M) with C_{70} (10^{-3} M) for selected protons (right plot: H_6 proton, left plot: H_7, 1:1 binding model).

For additional information see:

http://app.supramolecular.org/bindfit/view/f3cbc3e0-aacf-4516-852f-a773e3b87783

Computational methods

Optimized geometries of porphyrin **2H-PTetraCor** and the supramolecular assemblies C_{60}@**2H-PTetraCor**, C_{70}@**2H-PTetraCor**, $(C_{60})_2$@**2H-PTetraCor** and $(C_{70})_2$@**2H-PTetraCor** were obtained by DFT methods with the B97D3 functional, which contains the Becke-Johnson damping empirical dispersion correction and was provided by Grimme and collaborators.[2] Pople's split valence set 6-31G(d,p) was chosen as the basis set.[3] Solvent corrections applied using the polarizable continuum model (PCM) using toluene (ε=2.3741).[4]

The strategy to obtain the inclusion complexes C_{60}@**2H-PTetraCor** and C_{70}@**2H-PTetraCor** consisted of using the optimized structure of compound **2H-PTetraCor** and manually placing the corresponding fullerene molecule halfway between the two corannulenes, rotating the single C-C bonds at the same time so that both PAH fragments matched with the fullerene surface. For the assembly C_{70}@**2H-PTetraCor**, several attempts were carried out by imposing different orientations on the fullerene C_{70}; only the most stable one was considered and is reported here. Once both 1:1 adducts were optimized, their structures were used as starting geometries for a second round of optimizations to obtain the complexes $(C_{60})_2$@**2H-PTetraCor** and $(C_{70})_2$@**2H-PTetraCor** by placing a second fullerene molecule following the same protocol described above.

All minima were confirmed by vibrational analysis to show no imaginary frequencies. The electronic energies of the optimized geometries were further evaluated using a more extended 6-31+G(d,p) basis set that includes diffuse functions.[5]

Deformation energies were estimated by subtracting the electronic energy of the optimized porphyrin **2H-PTetraCor** (H) from the electronic energy of the porphyrin in the optimized structure of the adduct (HG, i.e., C_{60}@**2H-PTetraCor** and C_{70}@**2H-PTetraCor**) according to eq. 4. For the supramolecular assemblies $(C_{60})_2$@**2H-PTetraCor** and $(C_{70})_2$@**2H-PTetraCor**) (HG_2), the subtraction was carried out from the porphyrin structure shown in the parent 1:1 inclusion complexes (HG) according to eq. 5:

$$E_{def} = E_{HG}(H) - E_{H}(H) \quad \text{eq.4}$$

$$E_{def} = E_{HG_2}(H) - E_{HG}(H) \quad \text{eq.5}$$

Where the subscripts denote the geometry used and the letter between parentheses corresponds to the molecular entity studied (porphyrin in all cases).

Interaction energies were calculated taking into account basis set superposition error (BSSE) with the Boys–Bernardi functional counterpoise scheme[6] as follows (eq. 6):

$$E_{int}(HG) = E_{HG}^{HG}(HG) - E_{HG}^{HG}(H) - E_{HG}^{HG}(G) \quad \text{eq. 6}$$

Where the subscripts denote the geometry used (inclusion complex in all cases) and the superscripts refer to the basis set (from the supramolecular assembly in all cases); H and G correspond to the host and guest molecular entities, respectively, and HG to the supramolecular adduct.

All the above-described computational methods were performed using the Gaussian 16 package.[7]

Non-covalent interactions were obtained from the location critical points at which the reduced density gradient decreases to low electronic density values according to the scheme of Yang et al. with the help of the NCIPlot package.[8] Calculations were performed with promolecular densities, and gradient isosurfaces were plotted with an isovalue of 0.3 a.u. and coloured on a blue-green-red scale according to the values of the sign of λ_2 (the second eigenvalue of the electron-density Hessian). Red indicates repulsion, green indicates weak attraction, and blue represents strong attraction. Graphics were visualized in Chimera[9] with the help of Tangram NCIPlot GUI built by Insilichem Group.[10]

2H-PTetraCor (E= -4978.5409298 a.u.)

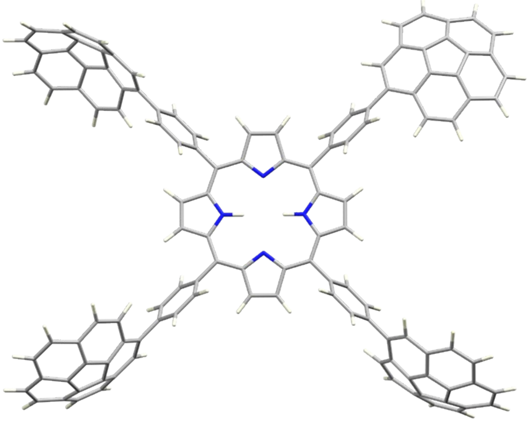

C	4.610866	5.415090	0.985592
C	3.612761	4.438911	0.956263
C	5.544501	5.532155	-0.066522
C	3.511902	3.542284	-0.125282
H	2.898444	4.364137	1.774962
C	5.441785	4.630954	-1.146897
C	4.442161	3.656242	-1.176985
H	6.165731	4.686134	-1.958979
H	4.375592	2.970519	-2.020618
C	2.444509	2.497847	-0.151124
C	2.876348	1.156575	-0.132273
C	4.233377	0.683475	-0.071625
C	4.208696	-0.694028	-0.096468
H	5.103272	1.325239	-0.019338
C	2.834503	-1.115998	-0.144418
H	5.055558	-1.367788	-0.081000
N	2.070214	0.034935	-0.168983
C	1.096284	2.927406	-0.202276
C	0.717578	4.334168	-0.348063
C	-0.644760	4.361517	-0.402656
H	1.404199	5.168640	-0.426918
C	-1.090015	2.971357	-0.289103
H	-1.288665	5.222732	-0.535309
N	-0.016255	2.117243	-0.176998
C	2.352203	-2.439896	-0.165396
C	0.988574	-2.816531	-0.223007
C	0.544176	-4.207726	-0.118573
C	-0.818323	-4.180362	-0.168581
H	1.188871	-5.069830	0.004426
C	-1.197809	-2.772646	-0.303474
H	-1.504558	-5.015646	-0.095108
N	-0.085508	-1.962001	-0.327242
C	-2.545925	-2.342806	-0.347038
C	-2.977715	-1.001738	-0.363655
C	-4.335200	-0.529354	-0.416448
C	-4.310740	0.848196	-0.395797
H	-5.204875	-1.172013	-0.460917
C	-2.936332	1.270821	-0.357491
H	-5.157750	1.521799	-0.407129
N	-2.171688	0.120231	-0.333914
C	-2.453981	2.594996	-0.343787
C	3.378063	-3.524136	-0.112935
C	3.531438	-4.419524	-1.189290
C	4.216805	-3.676845	1.008458
C	4.492751	-5.431520	-1.146572
H	2.887509	-4.315083	-2.061388
C	5.179799	-4.687369	1.050495
H	4.108507	-2.992592	1.848972
C	5.335689	-5.586583	-0.025106
H	5.834868	-4.772461	1.916664
C	-3.613975	-3.387539	-0.364928
C	-4.526449	-3.508586	0.700786
C	-3.730920	-4.276473	-1.450609
C	-5.519518	-4.491632	0.686498
H	-4.451144	-2.823780	1.544476
C	-4.723170	-5.260162	-1.465342
H	-3.034167	-4.190749	-2.283329
C	-5.630963	-5.390358	-0.394816
H	-4.795541	-5.948271	-2.307000
C	-3.479674	3.678595	-0.401334
C	-4.330466	3.814636	-1.515716
C	-3.622050	4.590742	0.663039
C	-5.292487	4.825778	-1.562593
H	-4.218699	3.130479	-2.355746
C	-4.584874	5.600804	0.617143
H	-2.981681	4.487089	1.537841
C	-5.438630	5.740309	-0.497419
H	-4.703301	6.271470	1.467268
H	4.586964	-6.123479	-1.982417
H	-6.231550	-4.549723	1.507765
H	4.664059	6.109221	1.823260
H	-5.922455	4.931971	-2.444705
C	-6.691170	-6.431387	-0.435640
C	-6.878598	-7.360300	0.680038
C	-7.574237	-6.441076	-1.529792
C	-7.844452	-8.343375	0.465673
C	-6.391617	-7.291922	2.046274
C	-8.710998	-7.328978	-1.613183
H	-7.454521	-5.656424	-2.277727
C	-8.736545	-8.331799	-0.645355
C	-8.486055	-9.037830	1.534873
H	-5.557295	-6.634949	2.285932
C	-7.023531	-7.967041	3.092233
C	-9.949950	-7.169158	-2.351300
C	-9.933766	-9.001595	-0.260406
C	-8.196109	-8.791163	2.876074
C	-9.778891	-9.438154	1.087633
H	-6.667511	-7.796784	4.109172
H	-9.995635	-6.445555	-3.166208
C	-11.124421	-7.820059	-1.969853
C	-11.176759	-8.697900	-0.816080
C	-9.244037	-9.172817	3.802295
C	-10.858990	-9.597396	1.956228
H	-12.047249	-7.581602	-2.500414
C	-12.312254	-9.071677	0.004462
C	-10.513647	-9.554573	3.363903
H	-9.073350	-9.066860	4.874435
C	-12.160961	-9.500688	1.324812
H	-13.321425	-8.921916	-0.381634
H	-11.290216	-9.731404	4.109270
H	-13.057479	-9.671344	1.922389
C	6.346543	-6.669926	0.010433
C	7.181975	-6.926599	-1.165988
C	6.557924	-7.388494	1.199451
C	7.996972	-8.054952	-1.077969
C	7.507730	-6.031979	-2.260426
C	7.577514	-8.403448	1.332310
H	5.962495	-7.116054	2.071383
C	8.186525	-8.780718	0.135860
C	9.170398	-8.223059	-1.873331
H	6.897705	-5.144315	-2.421035
C	8.655873	-6.196419	-3.037458
C	8.232807	-8.894467	2.529248
C	9.470414	-9.397052	0.090285
C	9.600065	-7.266575	-2.793878
C	10.079132	-9.053220	-1.152509
H	8.895445	-5.431281	-3.777104
H	7.782553	-8.697384	3.503005
C	9.489420	-9.499878	2.484375
C	10.212247	-9.674822	1.239606
C	11.008756	-7.336204	-3.130995
C	11.462931	-8.972020	-1.313308
H	9.978585	-9.755325	3.425327
C	11.638162	-9.813770	1.021474
C	11.896719	-8.148818	-2.425207
H	11.408912	-6.661704	-3.889161
C	12.234396	-9.479267	-0.195884
H	12.281220	-10.090567	1.858030
H	12.960814	-8.081959	-2.655787
H	13.322482	-9.506677	-0.268234
C	6.590974	6.580940	-0.027957
C	7.336603	6.822926	1.210399
C	6.920196	7.281269	-1.201012
C	8.190277	7.925463	1.180855
C	7.545347	5.929579	2.334112
C	7.978420	8.263288	-1.257988
H	6.388228	7.019436	-2.116159
C	8.499682	8.633617	-0.018709
C	9.299816	8.066415	2.067842
H	6.898073	5.061792	2.451387
C	8.631297	6.067298	3.200691
C	8.743345	8.723090	-2.401073
C	9.793754	9.211917	0.126753
C	9.624165	7.106558	3.027189
C	10.288747	8.862408	1.417509
H	8.786700	5.302471	3.962825
H	8.367772	8.530126	-3.406771
C	10.010097	9.291168	-2.258472
C	10.634527	9.456554	-0.960288
C	11.002252	7.137845	3.477481
C	11.651805	8.742130	1.691253
H	10.581411	9.522835	-3.158404
C	12.041548	9.555552	-0.627603

C	11.968716	7.917243	2.840717
H	11.318979	6.458980	4.270367
C	12.526477	9.215628	0.636782
H	12.758659	9.805289	-1.410740
H	13.008003	7.821344	3.157935
H	13.605406	9.211672	0.797390
C	-6.457856	6.813974	-0.565029
C	-7.811011	6.506730	-1.036990
C	-6.146522	8.102408	-0.098445
C	-8.645142	7.612272	-1.201917
C	-8.494272	5.227416	-1.065432
C	-7.109624	9.178805	-0.064022
H	-5.149287	8.268453	0.310157
C	-8.303503	8.916767	-0.735320
C	-10.069034	7.510922	-1.206597
H	-7.911861	4.311527	-0.977097
C	-9.886907	5.128786	-1.068925
C	-7.126582	10.368996	0.764382
C	-9.510744	9.619813	-0.455179
C	-10.738740	6.298862	-1.035418
C	-10.603163	8.751266	-0.747488
H	-10.336995	4.138552	-0.986338
H	-6.212137	10.678754	1.271913
C	-8.308746	11.058706	1.037279
C	-9.587849	10.627311	0.507853
C	-12.106429	6.419876	-0.568856
C	-11.832773	8.846028	-0.094496
H	-8.277622	11.884527	1.749320
C	-10.920546	10.874471	1.020682
C	-12.627864	7.633874	-0.120225
H	-12.716319	5.521093	-0.468468
C	-11.991047	10.025626	0.732909
H	-11.076108	11.686509	1.732241
H	-13.627856	7.642921	0.315526
H	-12.945771	10.204000	1.229465
H	1.053761	0.052817	-0.209021
H	-1.155077	0.102556	-0.298362

C_{60}@**2H-PTetraCor** (E= -7263.3537726 a.u.)

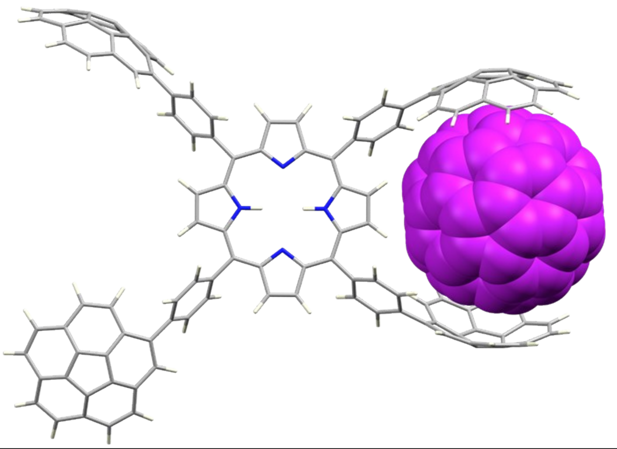

C	1.268664	5.224657	0.962646
C	0.113317	4.462660	0.772810
C	2.369997	5.111863	0.085754
C	0.007235	3.574044	-0.315271
H	-0.695257	4.509711	1.501021
C	2.214618	4.302879	-1.060206
C	1.050319	3.562407	-1.264121
H	3.041922	4.194671	-1.758844
H	0.973020	2.907052	-2.130172
C	-1.084696	2.556667	-0.361515
C	-0.644298	1.217387	-0.295391
C	0.718838	0.762927	-0.252001
C	0.710377	-0.610818	-0.233686
H	1.585944	1.405563	-0.240659
C	-0.655584	-1.053991	-0.203504
H	1.570117	-1.261863	-0.236174
N	-1.437913	0.084579	-0.262848
C	-2.436839	2.965763	-0.414365
C	-2.843668	4.356946	-0.605161
C	-4.207217	4.352841	-0.663159
H	-2.171454	5.200987	-0.710742
C	-4.623113	2.957254	-0.499912
H	-4.869361	5.194981	-0.825638
N	-3.531118	2.132871	-0.357429
C	-1.095077	-2.391735	-0.102546
C	-2.446716	-2.805610	-0.102435
C	-2.862697	-4.190750	0.112300
C	-4.227130	-4.190651	0.077292
H	-2.198123	-5.028459	0.290856
C	-4.633547	-2.802731	-0.158507
H	-4.897141	-5.029974	0.221449
N	-3.536342	-1.979129	-0.255885
C	-5.987639	-2.392961	-0.224291
C	-6.437237	-1.060940	-0.334625
C	-7.801560	-0.613558	-0.418677
C	-7.798946	0.762595	-0.488340
H	-8.661543	-1.270611	-0.421087
C	-6.431875	1.210138	-0.476615
H	-8.656952	1.419932	-0.543220
N	-5.649251	0.074461	-0.380217
C	-5.977238	2.543958	-0.537112
C	0.003804	-3.398989	-0.013574
C	0.186405	-4.374522	-1.013196
C	0.983568	-3.297094	0.996689
C	1.350785	-5.146832	-1.054192
H	-0.568698	-4.488321	-1.789845
C	2.158345	-4.044861	0.938324
H	0.848411	-2.568496	1.794278
C	2.387096	-4.958470	-0.113183
H	2.936748	-3.868710	1.678234
C	-7.041035	-3.450392	-0.162611
C	-7.957534	-3.500534	0.905553
C	-7.138658	-4.425510	-1.173897
C	-8.935575	-4.496753	0.965588
H	-7.897181	-2.749818	1.692425
C	-8.115422	-5.422741	-1.113926
H	-6.439044	-4.395664	-2.008068
C	-9.027522	-5.480948	-0.040612
H	-8.172335	-6.177090	-1.898053
C	-7.027762	3.599331	-0.653070
C	-7.872078	3.662605	-1.778721
C	-7.199843	4.557200	0.365641
C	-8.855837	4.648548	-1.881170
H	-7.738600	2.941183	-2.583692
C	-8.183911	5.542452	0.264035
H	-6.565249	4.509434	1.249458
C	-9.030473	5.609907	-0.862582
H	-8.324466	6.250035	1.080203
H	1.476771	-5.884798	-1.844870
H	-9.651360	-4.499637	1.785596
H	1.338454	5.888496	1.822906
H	-9.480887	4.697991	-2.771779
C	-10.072588	-6.537231	-0.004090
C	-10.261176	-7.372099	1.183631
C	-10.942600	-6.651148	-1.103000
C	-11.211692	-8.383016	1.042100
C	-9.791491	-7.183464	2.544705
C	-12.066140	-7.559088	-1.124127
H	-10.824916	-5.929942	-1.912573
C	-12.090216	-8.477446	-0.075781
C	-11.857321	-8.994286	2.158692
H	-8.967923	-6.498406	2.738188
C	-10.427589	-7.777052	3.636575
C	-13.297885	-7.479518	-1.886946
C	-13.283198	-9.129407	0.350761
C	-11.587041	-8.632129	3.477584
C	-13.139223	-9.448853	1.732396
H	-10.085633	-7.517739	4.639426
H	-13.343045	-6.827698	-2.760365
C	-14.468203	-8.112791	-1.465286
C	-14.523149	-8.891207	-0.242359
C	-12.641214	-8.949187	4.420940
C	-14.227736	-9.549609	2.599232
H	-15.387510	-7.932899	-2.024244
C	-15.663661	-9.210621	0.593988
C	-13.900366	-9.383873	4.002059
H	-12.485083	-8.751139	5.482201
C	-15.522992	-9.524602	1.947459
H	-16.669873	-9.108208	0.185300
H	-14.683792	-9.508168	4.750870
H	-16.424442	-9.656954	2.547307
C	3.696162	-5.635306	-0.260757
C	4.300955	-5.768223	-1.591544
C	4.428046	-5.996466	0.884931
C	5.509267	-6.466715	-1.626249
C	4.000615	-5.036453	-2.808922
C	5.780603	-6.496245	0.830190
H	3.975617	-5.810013	1.858522
C	6.231585	-6.822655	-0.448073
C	6.455442	-6.325144	-2.687228
H	3.073094	-4.472603	-2.877400
C	4.922068	-4.905695	-3.848355
C	6.811200	-6.460796	1.848781
C	7.615447	-6.899429	-0.777192
C	6.250181	-5.472267	-3.772317
C	7.754783	-6.592973	-2.162690
H	4.669904	-4.249186	-4.681555
H	6.531576	-6.260994	2.883570
C	8.167172	-6.531006	1.525051
C	8.623660	-6.649522	0.155067
C	7.456950	-5.057033	-4.460542
C	8.913235	-6.020797	-2.690408
H	8.899534	-6.381882	2.319063
C	9.897321	-6.265487	-0.418790
C	8.726318	-5.317151	-3.944232
H	7.373387	-4.427337	-5.346795
C	10.035405	-5.968445	-1.775612
H	10.750030	-6.098732	0.240068
H	9.591824	-4.882536	-4.445187
H	10.990251	-5.577023	-2.127685
C	3.670071	5.746381	0.403410
C	4.162316	5.725270	1.787305
C	4.497268	6.215425	-0.633157
C	5.372218	6.389861	1.993755
C	3.753142	4.874091	2.890092
C	5.844965	6.682511	-0.414706
H	4.125276	6.140901	-1.654766
C	6.193334	6.857075	0.923652
C	6.225381	6.113945	3.105399
H	2.817905	4.323072	2.819855
C	4.583525	4.613007	3.980636
C	6.957222	6.733862	-1.344364
C	7.546603	6.865648	1.369686
C	5.919973	5.159063	4.075158
C	7.567317	6.408948	2.719911
H	4.254911	3.877679	4.715562
H	6.760965	6.656854	-2.414182
C	8.282367	6.736857	-0.907314
C	8.625287	6.691841	0.500668
C	7.059576	4.659118	4.818304
C	8.670658	5.763267	3.279276
H	9.075760	6.658216	-1.651392
C	9.840654	6.216908	1.131467
C	8.371023	4.947200	4.438919
H	6.896078	3.947666	5.628207
C	9.862786	5.775373	2.455364
H	10.740673	6.095707	0.528040
H	9.186049	4.450115	4.965624
H	10.779633	5.326763	2.838797
C	-10.071153	6.657371	-0.989197
C	-11.414630	6.299273	-1.452576
C	-9.789252	7.973220	-0.584556
C	-12.270785	7.377681	-1.674663
C	-12.070668	5.005831	-1.422544
C	-10.774684	9.029519	-0.606987
H	-8.798004	8.179552	-0.179740
C	-11.959122	8.710087	-1.270161
C	-13.692207	7.246023	-1.680768
H	-11.469501	4.107874	-1.287796
C	-13.460873	4.877654	-1.427505
C	-10.821131	10.257703	0.162750
C	-13.182337	9.400131	-1.029776
C	-14.337213	6.029701	-1.454024
C	-14.254742	8.495483	-1.284500
H	-13.890568	3.883187	-1.299638
H	-9.916254	10.610803	0.659027
C	-12.018933	10.934634	0.396579
C	-13.285795	10.451172	-0.117068
C	-15.709502	6.143999	-0.999461
C	-15.489513	8.595532	-0.642099
H	-12.009003	11.794269	1.068049
C	-14.626169	10.694593	0.377219
C	-16.258691	7.366869	-0.612258

H	-16.300825	5.238345	-0.858029
C	-15.676992	9.810220	0.126361
H	-14.802694	11.536770	1.047684
H	-17.260880	7.375584	-0.181556
H	-16.637932	9.992244	0.609420
C	6.890882	-0.769406	3.364469
C	7.625077	-1.938241	2.889975
C	9.020890	-1.550159	2.704405
C	9.149317	-0.141634	3.062378
C	7.832612	0.341115	3.469865
C	9.626537	-2.703069	-0.644803
C	10.567569	-1.592704	-0.539422
C	10.646403	-1.200806	0.864765
C	9.754430	-2.070050	1.626092
C	9.124703	-2.999735	0.693216
C	10.818961	1.172205	0.163520
C	10.087396	2.342762	0.637198
C	9.585241	2.048405	1.977093
C	10.006632	0.695039	2.329630
C	10.769500	0.154566	1.209284
C	7.505491	3.286382	1.434882
C	6.113481	2.895501	1.619407
C	6.062265	1.880606	2.665959
C	7.426319	1.639591	3.126763
C	8.318173	2.507372	2.363753
C	4.260709	0.722897	1.418491
C	4.135592	-0.686859	1.059936
C	4.947464	-1.466264	1.990255
C	5.577814	-0.537229	2.923581
C	5.153869	0.815013	2.570334
C	5.576544	-2.983179	0.128518
C	6.891395	-3.469622	-0.279457
C	7.783944	-3.371734	0.871933
C	7.019812	-2.832071	1.992247
C	5.654272	-2.591236	1.532465
C	6.563804	-2.400848	-2.492299
C	7.454260	-1.534703	-3.256828
C	8.819343	-1.775999	-2.797078
C	8.768520	-2.791508	-1.750671
C	7.374787	-3.179469	-1.562947
C	9.724545	-0.707564	-2.696905
C	9.301099	0.644004	-3.050614
C	9.931318	1.572506	-2.117025
C	10.744195	0.794988	-1.186887
C	10.616356	-0.614136	-1.545120
C	9.225909	2.697729	-1.661114
C	7.860300	2.938724	-2.120852
C	7.096489	3.478788	-1.000770
C	7.987957	3.569471	0.150599
C	9.304422	3.089195	-0.257277
C	5.755768	3.106993	-0.821572
C	5.125276	2.178884	-1.755839
C	4.234779	1.308570	-0.992575
C	4.311489	1.701985	0.412388
C	5.253878	2.810996	0.516211
C	4.113851	-0.046123	-1.336657
C	4.875635	-0.587642	-2.457504
C	5.298686	-1.938551	-2.102598
C	4.795583	-2.235017	-0.764879
C	4.068216	-1.063469	-0.290327
C	5.732258	0.248517	-3.191702
C	5.859800	1.658124	-2.833697
C	7.254998	2.045920	-3.019729
C	7.989120	0.876023	-3.492975
C	7.047789	-0.234762	-3.599458
H	-2.454404	0.093004	-0.267322
H	-4.633113	0.072993	-0.347640

C_{70}@**2H-PTetraCor** (E= -7644.24904729 a.u.)

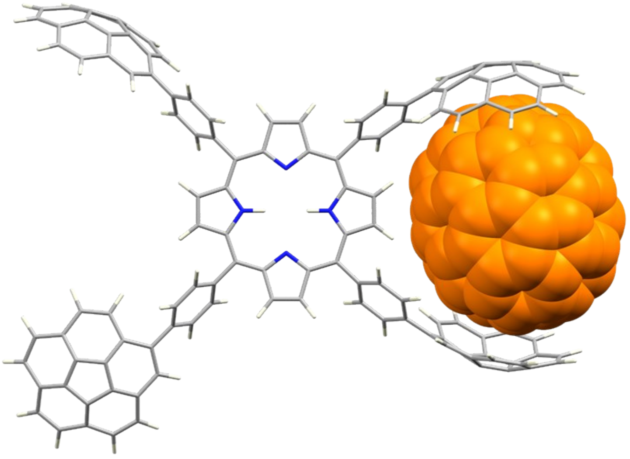

C	-0.937466	-5.228977	0.681288
C	0.185422	-4.401377	0.599610
C	-2.011425	-5.096805	-0.226322
C	0.290483	-3.422233	-0.408282
H	0.978217	-4.487126	1.341321
C	-1.870799	-4.159425	-1.272584
C	-0.738834	-3.352361	-1.370165
H	-2.680363	-4.024310	-1.986088
H	-0.665452	-2.622328	-2.174471
C	1.398550	-2.422820	-0.403233
C	0.994569	-1.072796	-0.323797
C	-0.355087	-0.583375	-0.253152
C	-0.314729	0.790917	-0.264854
H	-1.235952	-1.205170	-0.202963
C	1.062881	1.199677	-0.287252
H	-1.157746	1.464561	-0.254972
N	1.815701	0.040261	-0.327371
C	2.740506	-2.867787	-0.469838
C	3.109583	-4.265404	-0.693601
C	4.472956	-4.300002	-0.736539
H	2.416725	-5.087682	-0.829637
C	4.927195	-2.921931	-0.536977
H	5.111822	-5.157043	-0.913553
N	3.857987	-2.068561	-0.388346
C	1.545863	2.525539	-0.265225
C	2.909935	2.897873	-0.280546
C	3.361448	4.280795	-0.128088
C	4.725374	4.241253	-0.131101
H	2.717839	5.144358	-0.005365
C	5.096658	2.833170	-0.290935
H	5.416151	5.067651	-0.012697
N	3.978414	2.035716	-0.373808
C	6.439576	2.385060	-0.303642
C	6.854322	1.039072	-0.364409
C	8.207082	0.552102	-0.399150
C	8.166524	-0.824303	-0.450874
H	9.085072	1.184763	-0.384499
C	6.787182	-1.231826	-0.475224
H	9.006065	-1.506980	-0.471495
N	6.035327	-0.073490	-0.414441
C	6.294104	-2.551153	-0.545689
C	0.490673	3.582366	-0.247801
C	0.352137	4.484557	-1.321188
C	-0.471855	3.624175	0.781140
C	-0.754744	5.333570	-1.404110
H	1.098473	4.484645	-2.114434
C	-1.586343	4.456916	0.688159
H	-0.371014	2.951006	1.630951
C	-1.772934	5.302077	-0.426395
H	-2.354418	4.402682	1.456577
C	7.521811	3.413298	-0.239714
C	8.394821	3.476940	0.863405
C	7.692486	4.343474	-1.282846
C	9.401092	4.444474	0.926870
H	8.278387	2.759297	1.674502
C	8.697846	5.311875	-1.219738
H	7.027293	4.301919	-2.144266
C	9.565893	5.384720	-0.111459
H	8.811860	6.032188	-2.029238
C	7.313262	-3.638033	-0.642780
C	8.191775	-3.714299	-1.741381
C	7.420809	-4.614769	0.367095
C	9.146140	-4.730227	-1.825775
H	8.107665	-2.979054	-2.540455
C	8.375837	-5.629778	0.283749
H	6.759266	-4.558096	1.230345
C	9.256636	-5.709763	-0.815520
H	8.466739	-6.352156	1.093969
H	-0.849047	6.012281	-2.250491
H	10.082194	4.458144	1.775844
H	-0.994095	-5.978392	1.469088
H	9.797857	-4.788556	-2.696492
C	10.641761	6.409567	-0.069949
C	10.801856	7.288361	1.089816
C	11.564781	6.449860	-1.129968
C	11.789956	8.263099	0.952180
C	10.263644	7.172025	2.433452
C	12.717166	7.321326	-1.134715
H	11.461032	5.698803	-1.913908
C	12.722255	8.282532	-0.125223
C	12.402212	8.901855	2.072152
H	9.410728	6.520509	2.615331
C	10.866786	7.792581	3.529012
C	13.980034	7.171387	-1.833413
C	13.914604	8.915500	0.330741
C	12.059197	8.604816	3.390435
C	13.716682	9.298405	1.689405
H	10.470658	7.586646	4.524271
H	14.044860	6.481495	-2.675805
C	15.149411	7.786036	-1.382518
C	15.172484	8.614311	-0.191765
C	13.077984	8.929719	4.369346
C	14.766272	9.403088	2.602546
H	16.087515	7.553997	-1.888554
C	16.282475	8.934742	0.684325
C	14.368705	9.307635	3.993715
H	12.866255	8.781940	5.429160
C	16.089006	9.310868	2.015238
H	17.302817	8.784561	0.328947
H	15.120072	9.439851	4.773390
H	16.965218	9.441632	2.651703
C	-3.032888	6.062611	-0.596837
C	-3.685803	6.099181	-1.910191
C	-3.687376	6.587477	0.532224
C	-4.857322	6.856667	-1.969519
C	-3.478209	5.230771	-3.055204
C	-5.010234	7.161097	0.479184
H	-3.203858	6.468726	1.501603
C	-5.504768	7.373118	-0.807230
C	-5.858645	6.654422	-2.968891
H	-2.583120	4.614653	-3.101928
C	-4.452019	5.043137	-4.036564
C	-5.991252	7.290569	1.538505
C	-6.898365	7.485122	-1.083720
C	-5.745871	5.684191	-3.965256
C	-7.117687	7.041690	-2.421001
H	-4.270881	4.292058	-4.805962
H	-5.671747	7.190689	2.576223
C	-7.356246	7.394544	1.266903
C	-7.871023	7.385501	-0.087576
C	-7.002223	5.263254	-4.554566
C	-8.324664	6.474399	-2.833581
H	-8.056387	7.371155	2.102473
C	-9.185591	7.001671	-0.559953
C	-8.231865	5.638968	-4.014395
H	-6.990387	4.543029	-5.373120
C	-9.401954	6.570295	-1.869401
H	-10.010705	6.944210	0.150530
H	-9.139559	5.198556	-4.428038
H	-10.388214	6.188854	-2.135053
C	-3.261608	-5.873863	-0.060985
C	-3.808534	-6.105626	1.282708
C	-3.999292	-6.254531	-1.196150
C	-4.950208	-6.909035	1.325795
C	-3.532101	-5.397423	2.518937
C	-5.303897	-6.866741	-1.120051
H	-3.595879	-5.999918	-2.175683
C	-5.680790	-7.278257	0.156943
C	-5.865513	-6.896703	2.422182
H	-2.660776	-4.749293	2.578939
C	-4.423474	-5.392508	3.593267
C	-6.369017	-6.882473	-2.103405
C	-7.041359	-7.481707	0.525835
C	-5.695317	-6.080469	3.540297
C	-7.156063	-7.250810	1.927316
H	-4.200553	-4.749467	4.445094
H	-6.142782	-6.622887	-3.137881
C	-7.702721	-7.076708	-1.741055
C	-8.100417	-7.281727	-0.361849
C	-6.906223	-5.812908	4.290288
C	-8.339503	-6.809625	2.519996
H	-8.471469	-6.964650	-2.506235
C	-9.380162	-7.036967	0.270164
C	-8.167241	-6.157291	3.803009
H	-6.845121	-5.222182	5.204690
C	-9.493616	-6.812217	1.644350
H	-10.267683	-6.915104	-0.351601
H	-9.047279	-5.827372	4.355768
H	-10.465679	-6.523005	2.044930
C	10.267471	-6.788086	-0.923094
C	11.635836	-6.466811	-1.338255
C	9.931534	-8.099570	-0.546464

C	12.464391	-7.568859	-1.548649
C	12.330919	-5.195486	-1.268591
C	10.883552	-9.186296	-0.552874
H	8.921847	-8.279811	-0.176236
C	12.098119	-8.896008	-1.173573
C	13.888714	-7.482540	-1.507787
H	11.754463	-4.280631	-1.139821
C	13.723907	-5.111609	-1.227649
C	10.866564	-10.425500	0.200227
C	13.290687	-9.627823	-0.904345
C	14.564007	-6.290527	-1.243188
C	14.398615	-8.754497	-1.111854
H	14.180265	-4.133163	-1.071616
H	9.935482	-10.756137	0.662308
C	12.034332	-11.143354	0.462230
C	13.331621	-10.693576	-0.003816
C	15.916707	-6.454588	-0.747080
C	15.608318	-8.902369	-0.431997
H	11.975875	-12.011260	1.120456
C	14.646944	-10.985963	0.529575
C	16.414424	-7.699390	-0.360227
H	16.531352	-5.570204	-0.574076
C	15.732620	-10.132311	0.324902
H	14.774971	-11.841902	1.193571
H	17.401449	-7.745816	0.101898
H	16.671221	-10.351022	0.835877
C	-10.446052	-2.344391	-0.006633
C	-10.519985	-1.716875	1.304572
C	-9.516955	-3.371202	-0.246768
C	-9.669696	-2.140829	2.330639
C	-9.056278	-1.159208	3.210418
C	-9.365679	0.230294	3.110416
C	-8.376530	1.136716	3.596944
C	-8.962433	-2.373269	-2.447024
C	-9.918768	-1.306593	-2.187906
C	-8.758991	-3.386680	-1.494245

C	-10.644892	-1.292530	-0.993030
C	-10.857396	-0.036202	-0.293719
C	-10.417884	1.206532	-0.840368
C	-10.177825	2.259945	0.092345
C	-10.260154	0.677132	2.027886
C	-10.780165	-0.298169	1.125053
C	-10.099901	1.997830	1.511441
C	-6.311927	-3.258240	-1.858619
C	-6.528021	-2.202179	-2.837563
C	-7.405013	-3.837533	-1.192406
C	-7.826050	-1.769108	-3.126888
C	-8.090794	-0.350368	-3.299903
C	-7.039828	0.615124	-3.270768
C	-7.410470	1.946091	-2.912754
C	-9.653138	1.192000	-2.100097
C	-9.383546	-0.064783	-2.720860
C	-8.704764	2.232589	-2.334388
C	-6.159285	-3.772470	0.944365
C	-5.033490	-3.163458	0.253437
C	-7.325990	-4.104236	0.239290
C	-5.108270	-2.911760	-1.119085
C	-4.577289	-1.668425	-1.653426
C	-3.899137	-0.728234	-0.821276
C	-3.898911	0.627369	-1.265928
C	-5.671568	0.158496	-2.967297
C	-5.454440	-1.229583	-2.715232
C	-4.775195	1.066851	-2.328346
C	-8.711901	-3.209793	2.089173
C	-7.497690	-2.864123	2.812202
C	-8.630986	-3.812791	0.823694
C	-6.248386	-3.138654	2.251175
C	-5.175351	-2.167106	2.369915
C	-5.335022	-0.965756	3.122466
C	-4.492749	0.127656	2.758581
C	-3.819045	-0.995583	0.626787
C	-4.422093	-2.184081	1.135302

C	-3.742276	0.112850	1.523515
C	-6.654026	-0.673997	3.712644
C	-7.714558	-1.606699	3.507713
C	-7.035073	0.689020	3.894783
C	-4.343141	2.352252	1.978204
C	-4.870807	1.503639	3.036925
C	-6.122608	1.780127	3.595828
C	-5.094169	3.443783	1.507590
C	-4.500551	2.869119	-0.826241
C	-3.736885	1.732865	-0.335736
C	-3.656817	1.479216	1.037125
C	-5.173495	3.707953	0.075796
C	-7.148542	3.751160	-1.412734
C	-6.451146	2.874978	-2.341019
C	-5.153899	2.443179	-2.054394
C	-6.525711	4.164154	-0.225519
C	-8.633423	3.785313	1.023742
C	-9.271001	3.354122	-0.211661
C	-8.543709	3.338247	-1.405217
C	-7.284241	4.175738	1.021034
C	-6.898984	2.916634	3.121171
C	-8.295240	2.505161	3.114049
C	-9.143231	2.929165	2.085895
C	-6.399247	3.733785	2.092772
H	2.831486	0.008958	-0.359155
H	5.018978	-0.042268	-0.406968

$(C_{60})_2$@**2H-PTetraCor** (E= -9548.16104096 a.u.)

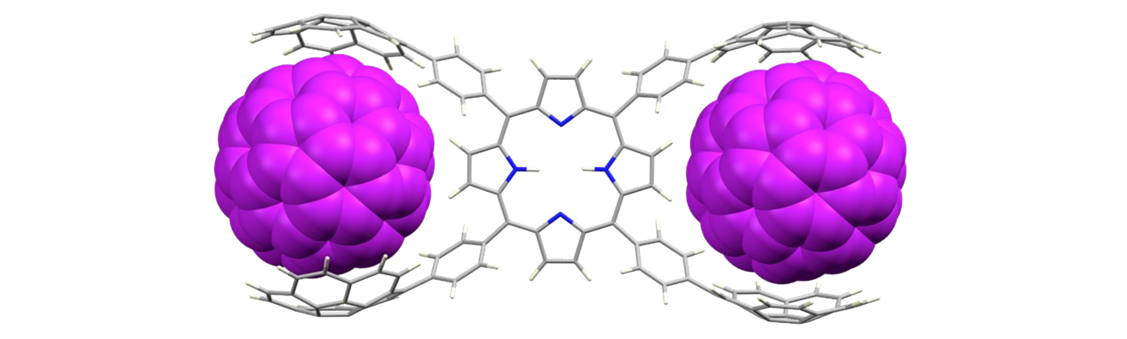

C	-4.849145	-5.298497	0.514237
C	-3.690199	-4.519881	0.468008
C	-5.916636	-5.072089	-0.382166
C	-3.547943	-3.496384	-0.489632
H	-2.908517	-4.662551	1.212953
C	-5.722356	-4.120890	-1.406267
C	-4.555264	-3.359394	-1.466803
H	-6.523694	-3.923138	-2.115361
H	-4.449356	-2.595020	-2.234957
C	-2.457032	-2.484826	-0.362934
C	-2.901874	-1.163607	-0.136516
C	-4.267540	-0.719204	-0.076622
C	-4.266417	0.642724	0.104399
H	-5.132566	-1.358626	-0.166541
C	-2.904594	1.084143	0.222851
H	-5.129964	1.286661	0.152254
N	-2.115469	-0.040092	0.055526
C	-1.103750	-2.890147	-0.424323
C	-0.682756	-4.242271	-0.791221
C	0.683286	-4.230691	-0.794268
H	-1.346196	-5.063363	-1.038923
C	1.083252	-2.874381	-0.417649
H	1.357796	-5.041172	-1.046000
N	-0.016834	-2.076114	-0.208037
C	-2.479747	2.409040	0.471293
C	-1.133287	2.835987	0.542407
C	-0.733771	4.203550	0.876047
C	0.632029	4.218213	0.862609
H	-1.408620	5.018971	1.110336
C	1.054087	2.857155	0.531660
H	1.294852	5.047257	1.083734
N	-0.032586	2.034705	0.346696
C	2.408697	2.452935	0.478849
C	2.851161	1.123215	0.296811
C	4.214040	0.668027	0.276335
C	4.206656	-0.696387	0.120971
H	5.081966	1.301260	0.373962
C	2.846527	-1.129648	-0.032644
H	5.065508	-1.347652	0.109637
N	2.060886	0.000728	0.110473
C	2.428762	-2.449944	-0.317854
C	-3.590040	3.395855	0.625633
C	-3.746269	4.469159	-0.273396
C	-4.602618	3.187549	1.586173
C	-4.912033	5.240157	-0.271688
H	-2.967292	4.664696	-1.009041
C	-5.780420	3.932478	1.564239
H	-4.489124	2.381539	2.309151
C	-5.978891	4.951741	0.607764
H	-6.582671	3.676019	2.253528
C	3.496702	3.473193	0.547948
C	4.542214	3.369566	1.486833

C	3.598339	4.475016	-0.438613
C	5.703987	4.133750	1.362377
H	4.474017	2.626272	2.279447
C	4.745367	5.264321	-0.541895
H	2.791206	4.593108	-1.160367
C	5.851703	5.069341	0.315901
H	4.809375	6.016395	-1.327340
C	3.545923	-3.422819	-0.508509
C	4.568413	-3.150124	-1.440954
C	3.702848	-4.550458	0.321995
C	5.748807	-3.891939	-1.461919
H	4.463300	-2.296669	-2.108341
C	4.868119	-5.320753	0.271585
H	2.923519	-4.793623	1.043028
C	5.940160	-4.981863	-0.585382
H	4.969047	-6.181448	0.931737
H	-5.014251	6.057977	-0.983176
H	6.532595	3.934444	2.034481
H	-4.949332	-6.067638	1.278490
H	6.555258	-3.567354	-2.111549
C	7.151185	5.723749	0.017293
C	8.073486	6.186279	1.060965
C	7.558832	5.723595	-1.334076
C	9.252109	6.762820	0.584125
C	8.082523	5.947947	2.496481
C	8.862702	6.149553	-1.774210
H	6.903054	5.231703	-2.051651
C	9.637072	6.755664	-0.787779
C	10.443692	6.846806	1.366996
H	7.168133	5.642099	2.999064
C	9.250834	6.015153	3.256751
C	9.591382	5.781235	-2.975139
C	11.058695	6.820179	-0.855434
C	10.530048	6.357561	2.668888
C	11.557584	6.874429	0.478280
H	9.202018	5.723038	4.306335
H	9.049171	5.344400	-3.814246
C	10.983700	5.841776	-3.040070
C	11.787581	6.273849	-1.911586
C	11.875984	6.054485	3.114077
C	12.815673	6.388008	0.833002
H	11.480076	5.447760	-3.927370
C	13.170010	5.969446	-1.595691
C	12.964072	6.066692	2.239134
H	12.033829	5.706679	4.135347
C	13.659597	6.024471	-0.289641
H	13.821742	5.580450	-2.378385
H	13.931255	5.724363	2.608070
H	14.675920	5.677836	-0.101081
C	-7.284767	5.639836	0.487726
C	-7.838523	5.926474	-0.841419
C	-8.061923	5.866939	1.638124

C	-9.046488	6.626505	-0.841587
C	-7.489213	5.342079	-2.123646
C	-9.413341	6.370187	1.589006
H	-7.646590	5.570580	2.600879
C	-9.815359	6.843701	0.340836
C	-9.949898	6.612674	-1.948161
H	-6.559183	4.786821	-2.221397
C	-8.368124	5.337410	-3.207174
C	-10.482758	6.217272	2.555323
C	-11.185487	6.960456	-0.030876
C	-9.699722	5.893605	-3.117544
C	-11.269628	6.818777	-1.447132
H	-8.081604	4.782768	-4.101087
H	-10.243183	5.897833	3.569949
C	-11.825329	6.326352	2.189190
C	-12.228513	6.604576	0.825606
C	-10.877274	5.563957	-3.896640
C	-12.404649	6.314007	-2.083276
H	-12.587460	6.086648	2.931300
C	-13.477442	6.291863	0.161178
C	-12.166648	5.762282	-3.402820
H	-10.757119	5.042536	-4.846698
C	-13.561460	6.156201	-1.225530
H	-14.354718	6.049954	0.761939
H	-13.010568	5.390300	-3.984552
H	-14.500582	5.810056	-1.658056
C	-7.225725	-5.739923	-0.198155
C	-7.774273	-5.883358	1.156816
C	-8.007916	-6.079766	-1.316725
C	-8.989391	-6.565821	1.232444
C	-7.413233	-5.170312	2.369043
C	-9.361709	-6.568575	-1.211406
H	-7.594174	-5.883029	-2.305634
C	-9.764131	-6.900671	0.081237
C	-9.888409	-6.423169	2.333010
H	-6.477211	-4.617192	2.403776
C	-8.288895	-5.039198	3.447421
C	-10.434971	-6.508073	-2.185334
C	-11.134548	-6.960832	0.467030
C	-9.626468	-5.590431	3.420871
C	-11.212273	-6.668160	1.859998
H	-7.993142	-4.397352	4.277712
H	-10.195920	-6.304488	-3.229522
C	-11.776918	-6.561896	-1.806587
C	-12.177552	-6.684236	-0.418620
C	-10.797524	-5.180290	4.170181
C	-12.340118	-6.092551	2.446305
H	-12.539409	-6.394614	-2.568102
C	-13.419671	-6.286766	0.213688
C	-12.091236	-5.419692	3.705653
H	-10.669991	-4.569847	5.064550
C	-13.497652	-6.005908	1.578688

H	-14.294822	-6.094108	-0.407519	C	-8.608411	1.212632	2.202631	C	10.344008	-0.701452	3.686954
H	-12.929154	-4.987407	4.253221	C	-9.287577	0.184153	2.985193	C	7.685791	1.205835	1.163772
H	-14.431130	-5.605504	1.975092	C	-8.850411	-1.119887	2.493474	C	14.131800	-1.207831	-0.696302
C	7.247462	-5.677392	-0.470422	C	-9.139209	2.947349	0.507943	C	10.742984	-1.960371	3.210097
C	8.104156	-5.949182	-1.631081	C	-10.430563	3.490790	0.097005	C	12.587727	-1.297757	-2.635832
C	7.728676	-5.921788	0.834463	C	-11.379881	3.264017	1.182846	C	14.311806	1.059174	0.294377
C	9.298058	-6.608202	-1.334204	C	-10.674322	2.587247	2.266709	C	10.578582	3.294611	1.561063
C	8.043554	-5.450589	-2.997280	C	-9.288043	2.390900	1.849251	C	13.051061	-1.123460	2.855011
C	9.045961	-6.433899	1.115400	C	-9.995538	2.691622	-2.209100	C	12.570848	-2.976366	-0.810798
H	7.119213	-5.566547	1.664828	C	-10.848925	1.931701	-3.115398	C	14.167350	0.525284	1.584874
C	9.755094	-6.853670	-0.007965	C	-12.234619	2.128324	-2.698029	C	7.648811	-0.525623	-1.117486
C	10.446046	-6.555858	-2.183636	C	-12.233459	3.010891	-1.536096	C	7.512120	-1.059420	0.173336
H	7.109206	-5.056918	-3.389116	C	-10.849821	3.360992	-1.234401	C	10.289138	-3.507950	-0.002515
C	9.171491	-5.377346	-3.816242	C	-13.146782	1.063923	-2.772099	C	14.294420	0.175648	-0.868124
C	9.843924	-6.316401	2.322692	C	-12.710078	-0.239306	-3.264311	C	12.756253	0.141426	-2.813511
C	11.176422	-6.944951	-0.032444	C	-13.389100	-1.267262	-2.481352	C	11.469874	0.700025	-3.218280
C	10.473853	-5.830872	-3.372856	C	-14.245010	-0.599625	-1.505676	C	13.015023	-2.854870	0.574086
C	11.603962	-6.758042	-1.378680	C	-14.095314	0.841002	-1.685194	C	11.070348	1.959250	-2.742045
H	9.072097	-4.894658	-4.789081	C	-12.710753	-2.445418	-2.129871	C	8.634928	-2.177033	1.922121
H	9.356781	-6.042207	3.258787	C	-11.324493	-2.641943	-2.547363	C	12.810236	2.942520	0.875094
C	11.236634	-6.406787	2.298293	C	-10.619288	-3.318940	-1.463767	C	7.834353	1.763076	-0.178435
C	11.971790	-6.622078	1.066116	C	-11.567542	-3.538815	-0.377069	C	13.979863	-1.762944	0.645823
C	11.799273	-5.455011	-3.824148	C	-12.860593	-3.001628	-0.788721	C	11.308390	0.392317	3.758244
C	12.851941	-6.227504	-1.704744	C	-9.288140	-2.983284	-1.174860	C	9.057746	-0.143141	3.282451
H	11.785817	-6.199642	3.217319	C	-8.608923	-1.955508	-1.958416	C	12.105201	-3.056542	1.622749
C	13.341886	-6.282940	0.736339	C	-7.754928	-1.191104	-1.053052	C	9.226073	1.296388	3.104077
C	12.931599	-5.642355	-3.028959	C	-7.903149	-1.749477	0.288841	C	10.505472	-0.393735	-3.290708
H	11.908728	-4.919171	-4.767492	C	-8.852863	-2.854635	0.211991	C	11.940329	2.707150	-1.839817
C	13.760737	-6.095278	-0.582753	C	-7.612805	0.193941	-1.225528	C	11.523534	3.500646	0.470895
H	14.044221	-6.060255	1.540026	C	-8.316553	0.872492	-2.309193	C	8.797796	2.856034	-0.105925
H	13.884036	-5.242018	-3.378058	C	-8.753077	2.174590	-1.815782	C	11.279408	2.606993	2.641551
H	14.774257	-5.733224	-0.758732	C	-8.316619	2.304533	-0.429212	C	10.534740	-2.607511	-2.172423
C	-10.620418	0.373568	3.385231	C	-7.617190	1.078175	-0.063534	C	9.004795	-2.945742	-0.406420
C	-11.326441	1.597422	3.019216	C	-9.137492	0.138215	-3.180203	C	9.241498	2.978729	1.278674
C	-12.712286	1.246801	2.719187	C	-9.287007	-1.302864	-3.000827	C	13.997073	-0.913137	1.763614
C	-12.862659	-0.193238	2.898553	C	-10.672252	-1.653121	-3.301253	C	7.815334	0.913544	-1.296551
C	-11.569469	-0.733082	3.309473	C	-11.378244	-0.428376	-3.665932	C	11.196545	-1.629365	-2.930558
C	-13.146242	2.798569	-0.492708	C	-10.429524	0.678629	-3.591170	C	13.325292	1.204009	2.565023
C	-14.094494	1.691673	-0.568138	H	-1.100057	-0.049028	0.083227	C	8.552923	1.958938	2.065208
C	-14.244940	1.135060	0.773069	H	1.046765	0.018585	0.054444	C	13.623152	2.294085	-0.066606
C	-13.389515	1.898769	1.676449	C	9.689495	2.175193	-2.317508	C	10.715469	-3.386028	1.327199
C	-12.711085	2.927922	0.894383	C	10.617367	1.627690	3.398448	C	9.872868	-2.711980	2.309896
C	-14.388883	-1.135389	-0.215891	C	12.123432	-2.175016	2.785243	C	9.177997	-0.185608	-2.884211
C	-13.685183	-2.360675	0.149240	C	11.097552	3.382861	-0.857801	C	8.193671	-2.295508	0.534704
C	-13.249837	-2.233259	1.537770	C	12.635437	0.184967	3.350727	C	11.235098	-3.294486	-1.092669
C	-13.684523	-0.928290	2.029150	C	13.261619	-1.959805	-1.596883	C	8.221468	-0.866514	2.417131
C	-14.389264	-0.250793	0.945746	C	8.488507	-1.205397	-2.098098				
C	-11.149222	-3.415596	0.954057	C	9.708875	3.054831	-1.153125				
C	-9.767106	-3.062876	1.253157	C	9.154000	-2.392100	-1.748569				
C	-9.765560	-2.180984	2.415218	C	13.593117	0.864027	-1.947621				
C	-11.150285	-1.984635	2.833542	C	8.761629	1.123939	-2.388746				
C	-12.005249	-2.746680	1.928654	C	13.178572	2.173368	-1.453299				
C	-7.900543	-0.898304	1.406470	C	7.525517	-0.176958	1.336044				
C	-7.753536	0.543088	1.226562	C	12.660782	2.390754	2.217491				

$(C_{70})_2$@**2H-PTetraCor** (E= -10309.9502828 a.u.)

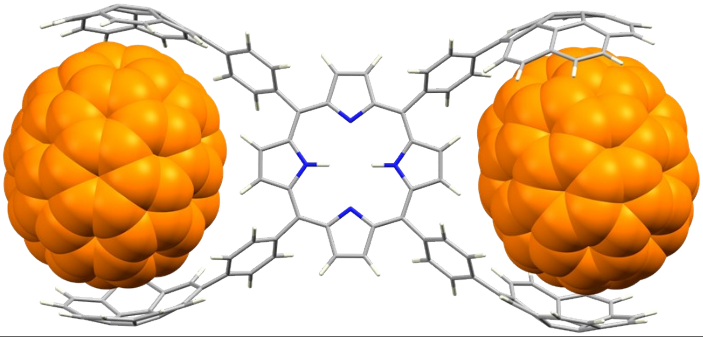

C	-4.870274	-5.216258	0.602796	C	4.229413	-0.613018	0.092918	H	-4.972563	-6.015275	1.335200
C	-3.756879	-4.373454	0.657644	H	5.103501	1.403281	0.149984	H	6.394606	-4.214312	-1.548820
C	-5.875825	-5.038670	-0.372879	C	2.859442	-1.050907	0.083696	C	7.141978	5.994896	0.659367
C	-3.588628	-3.334348	-0.279221	H	5.089379	-1.265303	0.042039	C	7.971556	6.273829	1.840470
H	-3.021813	-4.497421	1.451539	N	2.082178	0.090771	0.179028	C	7.627733	6.308314	-0.628888
C	-5.672295	-4.033703	-1.343553	C	2.397732	-2.382316	-0.002773	C	9.139704	6.992526	1.578629
C	-4.546301	-3.213660	-1.307972	C	-3.506160	3.642519	0.389770	C	7.913855	5.744401	3.196274
H	-6.429313	-3.859170	-2.104579	C	-3.590419	4.635860	-0.606165	C	8.920850	6.894335	-0.876094
H	-4.423441	-2.435111	-2.058945	C	-4.532622	3.582770	1.354124	H	7.053580	5.945609	-1.480546
C	-2.494742	-2.328958	-0.139543	C	-4.703491	5.476653	-0.686189	C	9.599839	7.306822	0.267811
C	-2.920413	-0.987670	-0.016564	H	-2.794831	4.714388	-1.345796	C	10.280590	6.954408	2.439046
C	-4.278676	-0.517497	-0.002212	C	-5.651834	4.409527	1.263184	H	6.987652	5.320641	3.574753
C	-4.258011	0.854459	0.086627	H	-4.474989	2.838270	2.146511	C	9.034215	5.687799	4.025233
H	-5.151379	-1.150837	-0.052619	C	-5.781131	5.347526	0.216905	C	9.737489	6.840604	-2.076758
C	-2.888465	1.278788	0.183678	H	-6.465833	4.280827	1.973222	C	11.017525	7.451706	0.311413
H	-5.109853	1.516979	0.090548	C	3.557218	3.548905	0.609764	C	10.324726	6.195929	3.606540
N	-2.117413	0.132289	0.107850	C	4.559516	3.344968	1.579291	C	11.437872	7.227097	1.654506
C	-1.145829	-2.758763	-0.128517	C	3.696130	4.659699	-0.247691	H	8.939660	5.183914	4.987763
C	-0.739046	-4.147152	-0.350376	C	5.698328	4.147367	1.625529	H	9.271603	6.571692	-3.025039
C	0.624896	-4.161152	-0.294858	H	4.466854	2.510455	2.271907	C	11.124059	6.984621	-2.033826
H	-1.406526	-4.977873	-0.547741	C	4.818351	5.488454	-0.173664	C	11.836771	7.195643	-0.786914
C	1.041066	-2.779483	-0.053309	H	2.931231	4.851004	-0.998947	C	11.658131	5.859134	4.063129
H	1.291323	-5.004381	-0.436286	C	5.871850	5.227977	0.733235	C	12.700816	6.730476	1.977227
N	-0.048877	-1.945283	0.041153	H	4.896422	6.339911	-0.848483	H	11.691205	6.823674	-2.951097
C	-2.432743	2.605378	0.344878	C	3.464971	-3.427089	0.012016	C	13.215694	6.894354	-0.452652
C	-1.078021	3.002030	0.433758	C	4.477711	-3.457580	-0.965446	C	12.789625	6.110775	3.285181
C	-0.662701	4.368794	0.749549	C	3.555664	-4.334292	1.086585	H	11.778559	5.302824	4.993265
C	0.701968	4.354584	0.792505	C	5.590193	-4.289329	-0.824351	C	13.626850	6.670636	0.862553
H	-1.329033	5.202042	0.941675	H	4.418894	-2.779120	-1.815167	H	13.932820	6.724695	-1.256276
C	1.108513	2.981358	0.490647	C	4.660521	-5.177561	1.219449	H	13.753401	5.737207	3.632557
H	1.369733	5.174756	1.029184	H	2.771140	-4.341567	1.842098	H	14.650657	6.338459	1.037439
N	0.011752	2.175380	0.290856	C	5.726045	-5.139996	0.292896	C	-7.040455	6.105080	0.030093
C	2.457559	2.554635	0.446404	H	4.720919	-5.856109	2.069585	C	-7.607309	6.246095	-1.315726
C	2.879352	1.218105	0.260877	H	-4.755310	6.222263	-1.474437	C	-7.775696	6.523886	1.153647
C	4.237628	0.760051	0.164443	H	6.488495	3.879919	2.317217	C	-8.784744	6.993104	-1.388603

C	-7.311769	5.479359	-2.512774
C	-9.101512	7.085684	1.061510
H	-7.354539	6.327612	2.139429
C	-9.514749	7.401709	-0.232303
C	-9.715674	6.865105	-2.465379
H	-6.405651	4.879027	-2.551322
C	-8.216628	5.364563	-3.568444
C	-10.151378	7.113856	2.060636
C	-10.889138	7.521694	-0.590079
C	-9.522624	5.983311	-3.529344
C	-11.013818	7.190871	-1.971430
H	-7.974011	4.682957	-4.384190
H	-9.898638	6.928553	3.104979
C	-11.497302	7.226501	1.709046
C	-11.922830	7.327652	0.327643
C	-10.730887	5.599285	-4.233111
C	-12.182006	6.647432	-2.508927
H	-12.249567	7.124246	2.491697
C	-13.197633	6.971712	-0.261294
C	-11.999011	5.914954	-3.746213
H	-10.653841	4.949973	-5.105671
C	-13.321189	6.650242	-1.613686
H	-14.066598	6.846071	0.385279
H	-12.870551	5.500688	-4.253954
H	-14.281808	6.282351	-1.975151
C	-7.120756	-5.841382	-0.356098
C	-7.761539	-6.179591	0.922207
C	-7.765760	-6.146178	-1.568269
C	-8.889849	-6.996816	0.819891
C	-7.589357	-5.564403	2.225298
C	-9.062181	-6.776115	-1.635799
H	-7.295281	-5.815763	-2.493667
C	-9.525799	-7.285571	-0.424469
C	-9.883899	-7.077486	1.842400
H	-6.736484	-4.912396	2.399159
C	-8.557436	-5.651276	3.227443
C	-10.051125	-6.729825	-2.694972
C	-10.906289	-7.531161	-0.174857
C	-9.810323	-6.347185	3.028387
C	-11.128247	-7.407532	1.227218
H	-8.408256	-5.072360	4.139274
H	-9.753124	-6.390924	-3.687506
C	-11.404632	-6.965037	-2.449010
C	-11.899780	-7.277052	-1.122424
C	-11.077809	-6.151001	3.703380
C	-12.359534	-7.025328	1.760493
H	-12.116602	-6.803948	-3.259112
C	-13.226578	-7.094267	-0.571925
C	-12.293425	-6.470611	3.098050
H	-11.094827	-5.631536	4.661889
C	-13.445375	-6.974584	0.802705
H	-14.067356	-6.936010	-1.248026
H	-13.217196	-6.193404	3.606661
H	-14.449170	-6.726976	1.149258
C	6.986263	-5.873123	0.576306
C	7.767871	-6.543756	-0.467951
C	7.504162	-5.757848	1.883311
C	8.921656	-7.183274	-0.011888
C	7.675521	-6.469026	-1.917669
C	8.797410	-6.260044	2.274013
H	6.955169	-5.127236	2.582092
C	9.422798	-7.054612	1.316000
C	10.026798	-7.483225	-0.864618
H	6.757292	-6.116796	-2.381944
C	8.760923	-6.749360	-2.748927
C	9.659593	-5.830440	3.360374
C	10.832356	-7.258826	1.284673
C	10.045941	-7.161220	-2.220463
C	11.206100	-7.522668	-0.064956
H	8.651926	-6.572461	-3.819457
H	9.236565	-5.243663	4.176201
C	11.040695	-6.026705	3.327364
C	11.698947	-6.670333	2.205597
C	11.371156	-7.061682	-2.799949
C	12.469745	-7.218981	-0.571249
H	11.647270	-5.583450	4.117692
C	13.071248	-6.552265	1.751145
C	12.525206	-7.087135	-2.014350
H	11.472414	-6.857460	-3.866212
C	13.438503	-6.814622	0.430132
H	13.822683	-6.143441	2.427173
H	13.485213	-6.898685	-2.495701
H	14.463226	-6.602426	0.124338
C	-14.353148	-2.388904	-0.554662
C	-14.518431	-1.868910	0.794739
C	-13.399095	-3.387710	-0.814608
C	-13.731276	-2.368762	1.837019
C	-13.186667	-1.457248	2.830182
C	-13.504216	-0.066066	2.821118
C	-12.558661	0.804149	3.441732
C	-12.714643	-2.213195	-2.887468
C	-13.697260	-1.176338	-2.607071
C	-12.561968	-3.298459	-2.007184
C	-14.499359	-1.262484	-1.465065
C	-14.770842	-0.067469	-0.683376
C	-14.310361	1.217612	-1.099409
C	-14.142716	2.194326	-0.072104
C	-14.332065	0.460846	1.721725
C	-14.782155	-0.441945	0.712148
C	-14.153548	1.819638	1.323680
C	-10.098153	-3.127129	-2.200067
C	-10.262173	-1.997392	-3.103969
C	-11.225437	-3.764259	-1.654680
C	-11.543560	-1.550005	-3.441820
C	-11.812203	-0.123487	-3.518022
C	-10.776057	0.842699	-3.343981
C	-11.183716	2.138524	-2.906043
C	-13.465727	1.308634	-2.303678
C	-13.142647	0.107223	-3.003473
C	-12.515714	2.370200	-2.392116
C	-10.120838	-3.863619	0.557247
C	-8.959739	-3.194420	-0.008377
C	-11.235963	-4.144048	-0.246604
C	-8.948431	-2.834241	-1.358545
C	-8.397915	-1.548984	-1.756340
C	-7.785177	-0.674614	-0.809554
C	-7.771442	0.712275	-1.143991
C	-9.425265	0.371104	-2.989318
C	-9.209618	-1.031443	-2.834727
C	-8.582139	1.230458	-2.222796
C	-12.748122	-3.409283	1.574179
C	-11.586968	-3.115536	2.400020
C	-12.579094	-3.908420	0.272689
C	-10.301175	-3.336578	1.901518
C	-9.248714	-2.371628	2.166853
C	-9.469729	-1.235676	3.000872
C	-8.617736	-0.111784	2.780840
C	-7.795374	-1.056572	0.614775
C	-8.417157	-2.285345	0.986551
C	-7.788922	-0.023163	1.600063
C	-10.827309	-0.999930	3.525157
C	-11.862135	-1.919322	3.177902
C	-11.234375	0.341841	3.789709
C	-8.442888	2.168940	2.191420
C	-9.028161	1.235196	3.142770
C	-10.316571	1.458667	3.638914
C	-9.174163	3.290092	1.761228
C	-8.424914	2.907950	-0.567283
C	-7.682122	1.740468	-0.120055
C	-7.687488	1.378261	1.230582
C	-9.163770	3.667925	0.352910
C	-11.038978	3.818872	-1.253002
C	-10.273546	3.024280	-2.200951
C	-8.992886	2.578161	-1.865497
C	-10.498654	4.139023	0.001397
C	-12.678365	3.648975	1.076417
C	-13.230092	3.314512	-0.228261
C	-12.427072	3.398515	-1.369262
C	-11.336078	4.045939	1.192727
C	-11.073243	2.624997	3.206683
C	-12.461583	2.207369	3.075767
C	-13.246102	2.707400	2.031513
C	-10.517269	3.524650	2.281271
H	-1.101563	0.111559	0.129629
H	1.066430	0.097409	0.210827
C	12.699975	2.159445	-2.984460
C	12.256108	-3.098415	1.646518
C	12.924925	3.014673	1.442763
C	9.428614	3.545489	0.833133
C	9.129857	-3.537111	-0.988237
C	7.859111	2.116094	-0.446005
C	7.638924	-0.227507	-1.329927
C	8.335848	1.526887	1.769623
C	8.438263	-2.559607	-1.816522
C	12.338281	3.729049	-1.259043
C	11.817316	3.215276	-2.516063
C	10.962354	-3.608063	-2.652555
C	7.512094	0.716820	-0.268642
C	12.203424	-0.872731	2.814177
C	11.603430	-2.132742	2.515182
C	8.367574	2.627641	0.817587
C	10.371974	-4.048925	-1.394004
C	8.232308	-0.964184	1.436239
C	12.700695	-3.850090	-1.075598
C	13.461316	-2.767354	1.020118
C	10.175418	-2.253145	2.319344
C	12.202199	1.039166	-3.714865
C	14.655352	0.363857	-0.368792
C	11.017195	-1.578207	-4.075370
C	9.889074	0.058904	2.998411
C	9.945983	-3.291918	1.329219
C	11.458168	4.099440	-0.229871
C	10.021513	3.978990	-0.426178
C	13.764690	2.008668	-2.018494
C	9.002049	-2.137546	-3.024623
C	14.051088	-0.302468	-2.695421
C	8.939125	-0.733290	-3.396538
C	14.345370	1.294391	0.668041
C	13.827957	2.619994	0.371484
C	11.675562	1.577986	3.020898
C	12.397706	-1.461200	-3.885878
C	13.541355	2.971306	-0.952124
C	8.233190	0.219262	-2.602142
C	10.285502	-2.673770	-3.454512
C	14.122733	-1.705407	-2.322614
C	9.318654	-1.116556	2.421358
C	9.895682	1.923622	-3.368713
C	13.692277	-3.154386	-0.365140
C	9.364651	1.383156	2.706307
C	10.466572	2.335870	2.731043
C	10.746952	0.916568	-3.914392
C	11.445193	-4.198169	-0.420641
C	14.354084	0.734566	-1.763043
C	11.231618	-3.828296	0.914344
C	13.471817	-0.521197	2.150812
C	10.183040	-0.388209	-4.045461
C	8.914592	-3.149481	0.397542
C	13.106025	-2.434155	-3.066714
C	7.790108	-1.592409	-0.945540
C	12.986425	-0.151805	-3.660682
C	10.435979	3.099100	-2.704922
C	12.879519	1.911259	2.390842
C	12.403330	-3.486190	-2.455815
C	8.082630	-1.958446	0.422636
C	14.408455	-2.057172	-0.999346
C	11.316051	0.179611	3.192711
C	8.421405	2.540944	-1.653625
C	9.521511	3.492783	-1.644588
C	11.758189	3.738984	1.149433
C	13.759492	0.856455	1.914487
C	7.799807	0.353199	1.099913
C	14.047324	-1.455095	1.237273
C	8.651107	1.578683	-2.719545
C	14.632688	-1.016842	-0.009283
C	10.503438	3.394871	1.808666

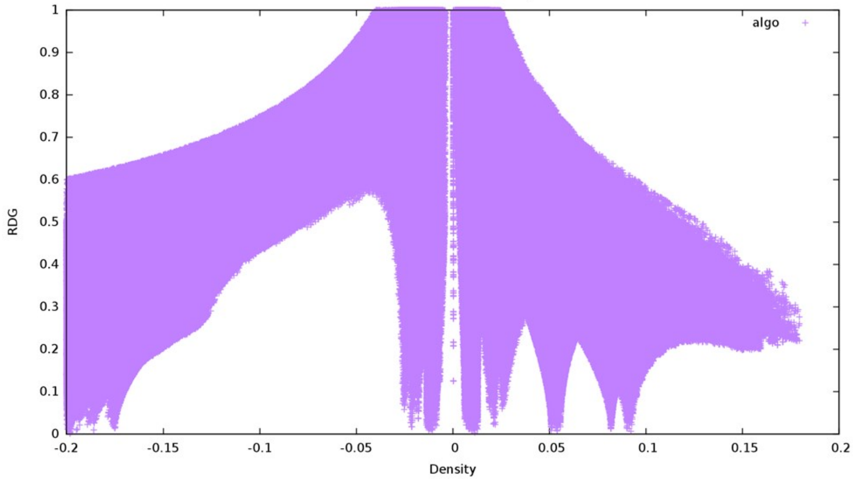

Figure S73: Plot of the reduced density gradient versus the electron density for the complex C_{60}@**2H-PTetraCor**.

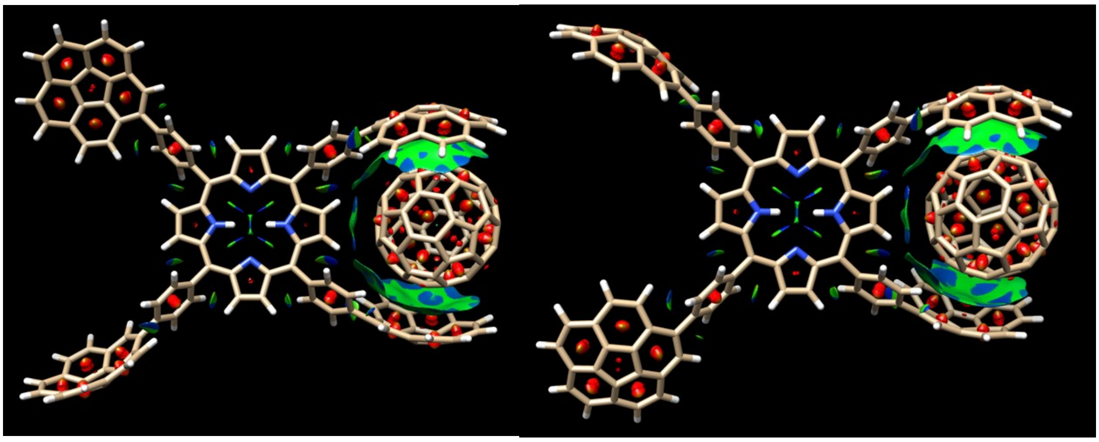

Figure S74: Non-covalent interaction isosurfaces for the assembly C_{60}@**2H-PTetraCor**.

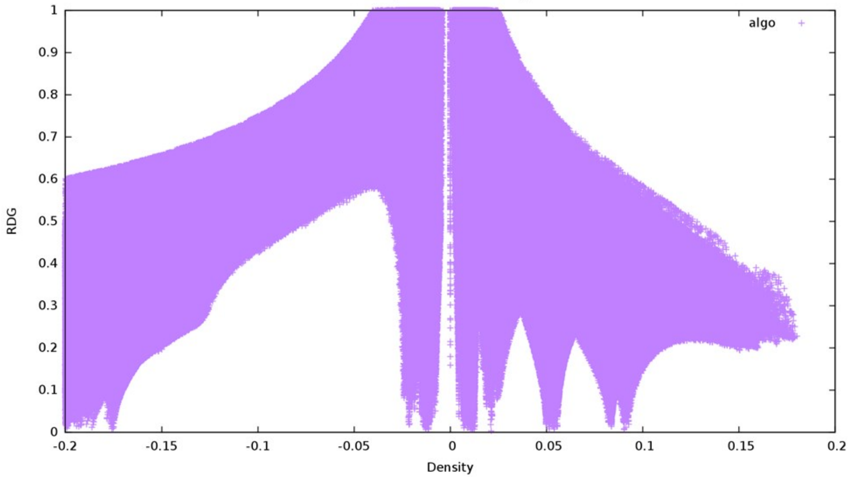

Figure S75: Plot of the reduced density gradient versus the electron density for the complex C_{70}@**2H-PTetraCor**.

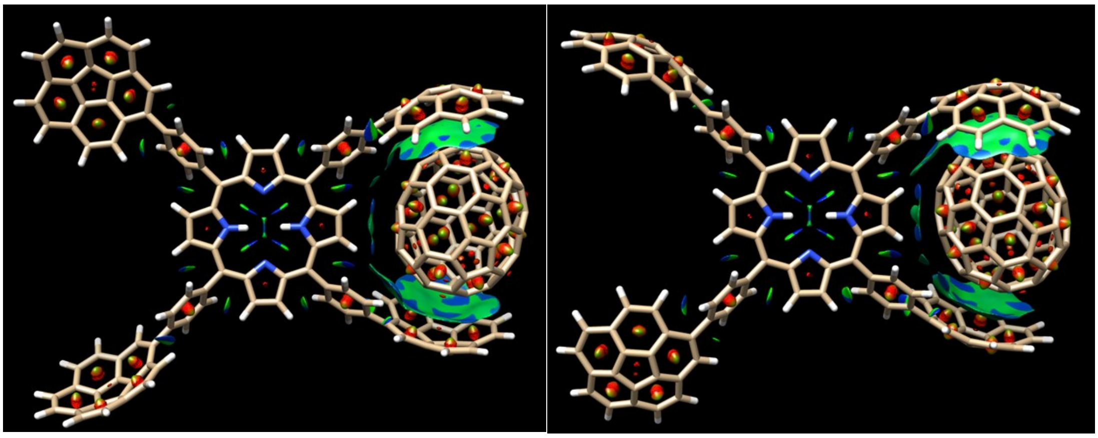

Figure S76: Non-covalent interaction isosurfaces for the assembly C_{70}@**2H-PTetraCor**

References

1. (a) L. Fielding, *Tetrahedron*, 2000, **56**, 6151-6170; (b) P. Thordarson, *Chem. Soc. Rev.*, 2011, **40**, 1305-1323; (c) P. Thordarson, in *Supramolecular Chemistry: From Molecules to Nanomaterials*, eds. P. A. Gale and J. W. Steed, John Wiley & Sons, Chichester, UK, 2012, vol. 2, pp. 239-274; (d) D. Brynn Hibbert and P. Thordarson, *Chem. Commun.*, 2016, **52**, 12792-12805.
2. (a) S. Grimme, *J. Comput. Chem.*, 2006, **27**, 1787-1799; (b) S. Grimme, S. Ehrlich and L. Goerigk, *J. Comput. Chem.*, 2011, **32**, 1456-1465.
3. (a) R. Ditchfield, W. J. Hehre and J. A. Pople, *J. Chem. Phys.*, 1971, **54**, 724-728; (b) W. J. Hehre, R. Ditchfield and J. A. Pople, *J. Chem. Phys.*, 1972, **56**, 2257-2261; (c) M. M. Francl, W. J. Pietro, W. J. Hehre, J. S. Binkley, M. S. Gordon, D. J. DeFrees and J. A. Pople, *J. Chem. Phys.*, 1982, **77**, 3654-3665.
4. G. Scalmani and M. J. Frisch, *J. Chem. Phys.*, 2010, **132**, 114110.
5. M. J. Frisch, J. A. Pople and J. S. Binkley, *J. Chem. Phys.*, 1984, **80**, 3265-3269.
6. (a) S. F. Boys and F. Bernardi, *Mol. Phys.*, 1970, **19**, 553-566; (b) S. Simon, M. Duran and J. J. Dannenberg, *J. Chem. Phys.*, 1996, **105**, 11024-11031; (c) T. Van Mourik, A. K. Wilson, K. A. Peterson, D. E. Woon and T. H. Dunning, in *Adv. Quantum Chem.*, eds. J. R. Sabin, M. C. Zerner, E. Brändas, S. Wilson, J. Maruani, Y. G. Smeyers, P. J. Grout and R. McWeeny, Academic Press, 1998, vol. 31, pp. 105-135; (d) K. N. Kirschner, J. B. Sorensen and J. P. Bowen, *J. Chem. Educ.*, 2007, **84**, 1225-1229.
7. M. J. Frisch, G. W. Trucks, H. B. Schlegel, G. E. Scuseria, M. A. Robb, J. R. Cheeseman, G. Scalmani, V. Barone, G. A. Petersson, H. Nakatsuji, X. Li, M. Caricato, A. V. Marenich, J. Bloino, B. G. Janesko, R. Gomperts, B. Mennucci, H. P. Hratchian, J. V. Ortiz, A. F. Izmaylov, J. L. Sonnenberg, Williams, F. Ding, F. Lipparini, F. Egidi, J. Goings, B. Peng, A. Petrone, T. Henderson, D. Ranasinghe, V. G. Zakrzewski, J. Gao, N. Rega, G. Zheng, W. Liang, M. Hada, M. Ehara, K. Toyota, R. Fukuda, J. Hasegawa, M. Ishida, T. Nakajima, Y. Honda, O. Kitao, H. Nakai, T. Vreven, K. Throssell, J. A. Montgomery Jr., J. E. Peralta, F. Ogliaro, M. J. Bearpark, J. J. Heyd, E. N. Brothers, K. N. Kudin, V. N. Staroverov, T. A. Keith, R. Kobayashi, J. Normand, K. Raghavachari, A. P. Rendell, J. C. Burant, S. S. Iyengar, J. Tomasi, M. Cossi, J. M. Millam, M. Klene, C. Adamo, R. Cammi, J. W. Ochterski, R. L. Martin, K. Morokuma, O. Farkas, J. B. Foresman and D. J. Fox, *Gaussian 16 Rev. C.01*, Wallingford, CT, 2016.
8. (a) E. R. Johnson, S. Keinan, P. Mori-Sánchez, J. Contreras-García, A. J. Cohen and W. Yang, *J. Am. Chem. Soc.*, 2010, **132**, 6498-6506; (b) J. Contreras-García, E. R. Johnson, S. Keinan, R. Chaudret, J.-P. Piquemal, D. N. Beratan and W. Yang, *J. Chem. Theory Comput.*, 2011, **7**, 625-632.
9. E. F. Pettersen, T. D. Goddard, C. C. Huang, G. S. Couch, D. M. Greenblatt, E. C. Meng and T. E. Ferrin, *J. Comput. Chem.*, 2004, **25**, 1605-1612.
10. Rodríguez-Guerra Pedregal, J. Development and Application of a Computational Platform for Complex Molecular Design, Universitat Autònoma de Barcelona, 2018. ISBN 9788449082382. https://ddd.uab.cat/record/9788449201498. (https://github.com/insilichem/tangram_nciplot).
